# Adaptive evolution of Agaricomycetes laccases follows wood lignin diversification in plants

**DOI:** 10.1128/aem.01971-25

**Published:** 2026-01-13

**Authors:** Shenglong Liu, Qinbiao Yu, Tian Yin, Xinlei Zhang, Rongrong Zhou, Chenkai Wang, Yazhong Xiao, Juanjuan Liu, Zemin Fang

**Affiliations:** 1 School of Life Sciences and Medical Engineering, Anhui University 428675 , Hefei, Anhui, China; 2 Anhui Key Laboratory of Biocatalysis and Modern Biomanufacturing, Hefei, Anhui, China; 3 Anhui Provincial Engineering Technology Research Center of Microorganisms and Biocatalysis, Hefei, Anhui, China; Royal Botanic Gardens Kew, Surrey, United Kingdom

**Keywords:** Agaricomycetes fungi, laccase, evolution, lignin, preference

## Abstract

Laccases are present as isozymes in white-rot fungi, yet their evolutionary history and functional role in lignin degradation remain controversial. *Trametes hirsuta*, a ubiquitous fungus in forest ecosystems, can completely break down cellulose, hemicellulose, and lignin in wood. Based on bioinformatic and biochemical characterization, we have shown that five laccase isozyme genes (*lacA–E*) in *Trametes hirsuta* AH28-2 were derived from a single ancestral laccase gene, *lacF*, with *lacA* and *lacB* originating from disparate evolutionary branches. The syringyl-type (S-type) lignin model compounds significantly induced the expression of *lacA–lacE* at both transcriptional and expression levels. Furthermore, *in vitro* and *in vivo* analyses demonstrated that the later-emerging laccases, LacA and LacB, primarily contribute to oxidizing S-type lignin present in hardwood, whereas laccase LacF plays a primary role in oxidizing guaiacyl-type (G-type) lignin found in softwood. Finally, evolutionary analysis of ancestral laccases from Agaricomycetes fungi also revealed a shift from better oxidation of G-type lignin in softwood by earlier ancestral laccases to better oxidation of S-type lignin in hardwood by later ancestral laccases. These findings indicate that laccase evolution in Agaricomycetes fungi is consistent with lignin synthesis. We have demonstrated the direct involvement of laccases at different evolutionary stages in preferentially oxidizing different types of lignin.

**IMPORTANCE:**

Laccases in white-rot fungi always exist in the form of isozymes. However, the evolutionary history and functional diversification of laccase isozymes remain controversial. Our study demonstrates that the six laccase isozymes in *Trametes hirsuta* AH28-2 belong to three distinct evolutionary branches. Among them, LacF represents an earlier-emerging branch and primarily contributes to oxidizing the G-type units of gymnosperm lignin. In contrast, LacA and LacB, which are later-emerging, primarily contribute to oxidizing the S-type units in angiosperm lignin. Interestingly, ancestral laccases reconstructed at different evolutionary nodes also display distinct lignin oxidation preferences. This suggests that the evolution of laccases in Agaricomycetes fungi is closely linked to the emergence of S-type lignin units in angiosperms. These findings reveal the co-evolutionary relationship between lignin structural changes and fungal laccase diversification, providing new insights into the evolutionary mechanisms and biological functions of laccase isozymes.

## INTRODUCTION

Plants are the primary reservoirs of organic carbon in terrestrial ecosystems and play a crucial role in global carbon cycling. Lignin, one of the main components of plant cells, is the second most abundant constituent in the cell wall of vascular plants, accounting for approximately 20% of the total carbon fixed by photosynthesis in land ecosystems (1). Lignin is formed through the free radical coupling of monolignol precursors derived from three *p*-hydroxycinnamyl alcohols with varying degrees of methoxylation, resulting in guaiacyl type (G-type), syringyl type (S-type), and *p*-hydroxyphenyl type (H-type) subunits (2, 3). Notably, the most distinctive variation in the composition of lignin in vascular land plants occurs between the two main groups of seed plants (4), with gymnosperm lignin mainly consisting of G-type units, while angiosperm lignin also includes S-type units. Minor amounts of H-type units are present in both types of lignin (5). Due to its heterogeneous and complex structure, lignin is difficult to degrade. While most bacteria and fungi cannot degrade lignin, white-rot fungi efficiently decompose and utilize the polymer.

The biological degradation of lignin by white-rot fungi has been described as an enzymatic combustion (6) and is typically carried out by fungi of the orders Polyporales and other Agaricales. The degradation process involves an array of extracellular lignin-modifying enzymes (LMEs), including manganese peroxidases (MnP, EC1.11.1.13), versatile peroxidases (VP, EC1.11.1.16), lignin peroxidases (LiP, EC1.11.1.14), and laccases (EC1.10.3.2) (7, 8). Although a definitive, universally accepted mechanism remains elusive, a substantial and compelling body of research has strongly implicated certain enzymes, particularly those produced by white-rot fungi and some bacteria, as central agents in the complex process of lignin degradation. The involvement of these enzymes is not always direct; rather, they function within a sophisticated synergistic system, both cleaving the recalcitrant structure of lignin itself and facilitating the activity of other enzymes (7, 9–12). For example, studies carried out by Nakazawa et al. (7) on *Pleurotus ostreatus* showed that LMEs, especially MnP and VP, play a crucial role in the degradation of natural lignin (7). In addition, based on the investigations on *Phanerodontia chrysosporium*, MnP and LiP were believed to be the only two lignin-depolymerizing enzymes, while laccase was considered to be less important for lignin degradation for a long time (13). However, it has been confirmed that the white-rot fungi *Dichomitus squalens* and *Ceriporiopsis subvermispora* retain good lignin degradation abilities without expressing detectable LiP (10, 14). Furthermore, the white-rot fungus *Pycnoporus cinnabarinus* demonstrated effective lignin degradation even when only laccase activity was detected, and both MnP and LiP were lacking; notably, its laccase-less mutants lost the delignification ability (15, 16). Hence, the role of laccase in lignin degradation by fungi was controversial.

Fungi generally produce several laccase isozymes that are encoded by complex multigene families (17–20). Although phylogenetic trees of many fungal laccases have been constructed for different purposes, their roles and functions within organisms remain poorly understood (21, 22). At the moment, it has been suggested that the multiple laccase isozyme genes in Polyporales during the early Cretaceous may be associated with the rapid radiation of angiosperms (18). The newly introduced S-type lignin in them was more resistant to degradation and led to more types of degraded phenolic derivatives that needed to be detoxified. The expansion of the laccase gene is deduced to be related to the emergence of S-type units in angiosperm lignin. In recent years, research using model compounds has elucidated the functional diversification of laccase within the multigene family (18, 23). Concurrently, considerable attention has been devoted to the application of laccases in natural lignin removal during biomass pretreatment (24–27). Nevertheless, despite these advances, it remains unclear whether laccases at different evolutionary stages exhibit functional preferences in the oxidation of lignin. More investigation should be carried out into the role of laccases during lignin degradation.

*Trametes* species are one of the typical white-rot fungi that are geographically found in almost all forest ecosystems from temperate to boreal zones, with temperate hardwood forests being the most widely distributed (28). Recent studies have shown that they are also found in tropical regions, further highlighting their worldwide distribution pattern and significant contribution to the global carbon cycle (29). More recently, several aspects of laccase evolution were addressed by ancestral sequence reconstruction and characterization of the resurrected ancestors using simple substrates (30). In this research, we employed the white-rot fungus *Trametes hirsuta* AH28-2, isolated from decayed wood, to investigate the function of laccase isozymes in lignin degradation. A previous study demonstrated that this strain can be induced by lignin model compounds to secrete high levels of extracellular laccase (31). We aimed to answer the following questions: (i) the function of each laccase isozyme; (ii) the driving force of evolution triggering laccase diversification. Distinct types of wood (larch, poplar, and bamboo) were used here, and the evolutionary relationship and functional characteristics of laccase isozymes derived from *T. hirsuta* AH28-2 were analyzed through both *in vivo* and *in vitro* analyses. In addition, to further validate the universality of laccase evolution, the study also explored how the ancestral laccases modified lignin throughout their evolutionary history. Together, these studies elucidate the potential mechanisms underlying the natural evolution of laccase isozymes.

## RESULTS

### Identification and evolutionary analysis of the laccase gene family in *T. hirsuta* AH28-2

**Fig 1:**
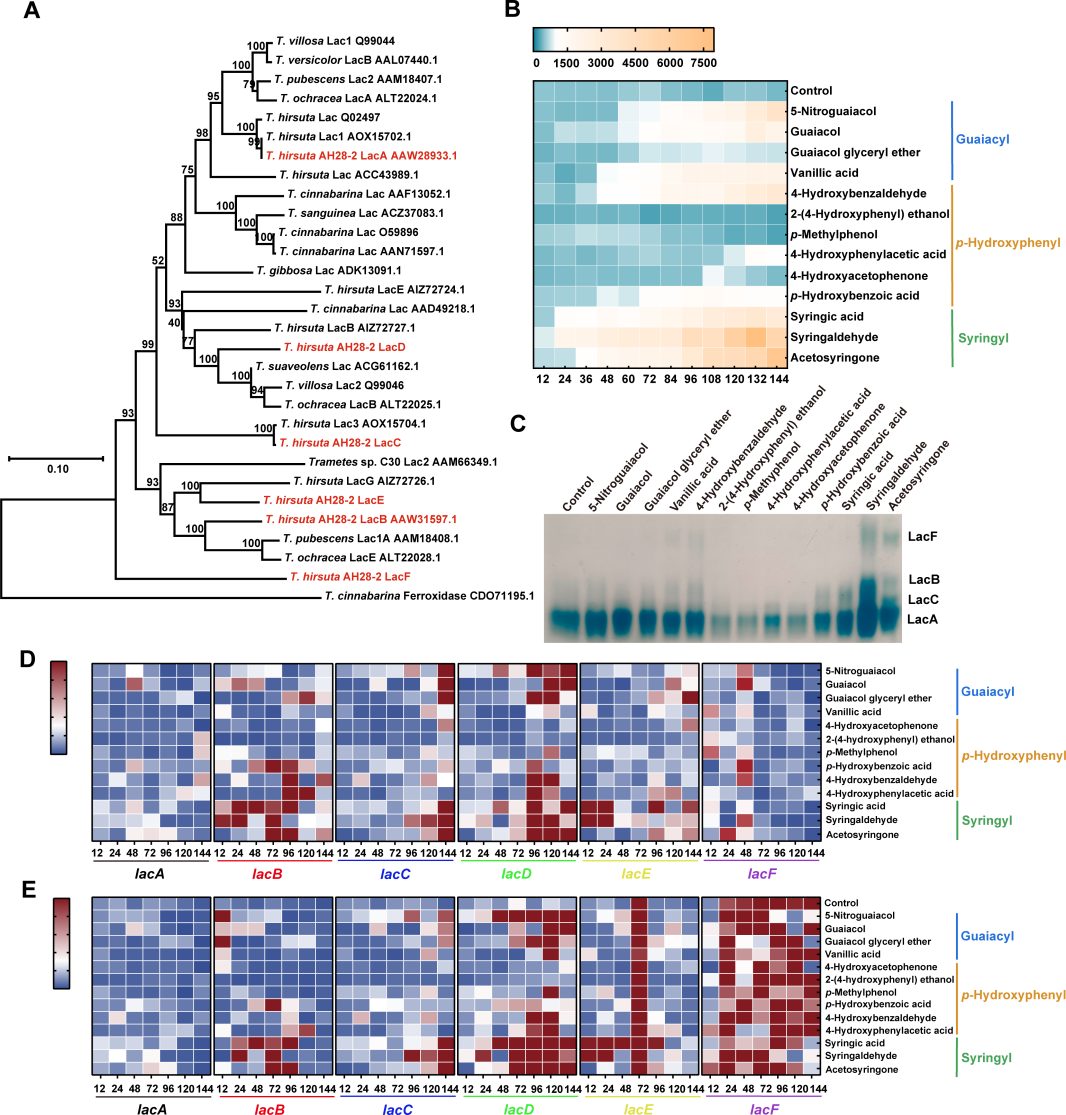
Analysis of evolution and expression levels of laccase isoenzymes from *T. hirsuta* AH28-2. (**A**) The phylogenetic tree of laccases in *Trametes*. (**B**) Changes in laccase activity of *T. hirsuta* AH28-2 exposed to lignin model compounds. (**C**) Laccase isoenzyme spectra of *T. hirsuta* AH28-2 exposed to lignin model compounds. (**D and E**) Transcription levels of *laccase* (*lacA*, *lacB*, *lacC*, *lacD*, *lacE*, and *lacF*) in *T. hirsuta* AH28-2 culture grown in XH medium after treatment with various lignin monomer compounds for 12, 24, 48, 72, 96, 120, and 144 h. The group without an aromatic compound added at 12 h was set as the control in panel **D**. The group without aromatic compounds added at the corresponding time point was set as the control in panel **E**.

Six laccase isozyme genes, *lacA–lacF*, were identified in the genome of *T. hirsuta* AH28-2 (Fig. S1), including four (*lacA*, *lacB*, *lacC*, and *lacF*) that have been reported previously (32, 33). All isozymes contained four typical Cu^2+^-binding motifs that are conserved among fungal laccases (Fig. S2). *lacA–lacF* were divided into three groups (I, II, and III), with group I containing *lacA*, *lacC*, and *lacD*, group II containing *lacB* and *lacE*, and group III containing only *lacF* (Fig. S3). The evolutionary analysis of laccase genes from *Trametes* species also supported this result (Fig. 1A). The close evolutionary relationship among the laccase sequences within the same species suggested that laccase sequences preserved a certain degree of conservation throughout evolution, potentially related to their essential functions *in vivo*. Moreover, the identification of 15 conserved motifs among *lacA–lacF* indicated that *lacA*, *lacC*, and *lacD* exhibited similarities in both composition and location. Similarly, *lacB* and *lacE* also demonstrated comparable characteristics (Fig. S4). This observation further revealed their functional similarity or a shared evolutionary origin.

### The activities and transcriptional levels of laccase isozymes in *T. hirsuta* AH28-2 exposed to lignin model compounds

Thirteen aromatic compounds that were all derived from three distinct lignin monomers were employed to induce laccase expression in *T. hirsuta* AH28-2 (Fig. 1B). Among the phenolics tested, syringyl derivatives exhibited the most effective promotion, demonstrating that syringyl compounds were excellent inducers of the laccase activity of *T. hirsuta* AH28-2. In contrast, differences were observed in the overall levels of laccase activity upon the addition of guaiacyl and *p*-hydroxyphenyl compounds. Notably, the addition of guaiacol glyceryl ether, 4-hydroxyacetophenone, *p*-methylphenol, and 2-(4-hydroxyphenyl) ethanol resulted in no substantial changes in laccase activity. Native polyacrylamide gel electrophoresis (PAGE) analysis revealed that LacA was the main laccase isozyme expressed in *T. hirsuta* AH28-2 (Fig. 1C). Among lignin monomers, syringic acid, syringaldehyde, and acetosyringone could induce LacA, LacB, and LacC expression (Fig. 1C).

The transcriptional patterns of the six laccase isozyme genes exhibited notable differences at different time points when three different types of aromatic compounds were added (Fig. 1D and E). In contrast to the control group, the expression of laccase *lacA* was consistently observed at a stable level throughout the entire culture period. Concurrently, an increase in *lacF* transcriptional levels was detected from 12 to 48 h, followed by a decrease after 72 h post-chemical addition (Fig. 1D). In comparison to *lacF*, transcriptional levels of the genes *lacB–lacE* exhibited an elevation approximately 48 h after the addition of guaiacyl and syringyl compounds. Interestingly, high transcription levels of *lacF* were observed throughout the entire culture process in the control sample without monolignolic compound addition at 12 h (Fig. 1E). In the case of syringyl substances, the transcription levels of laccase genes *lacA–lacE* were found to be relatively higher than those observed with the other two types of aromatic inducers. Thus, it appeared that *lacA–lacE* of *T. hirsuta* AH28-2 exhibited higher sensitivity to syringyl compounds compared to *lacF*.

### Analysis of the substrate spectrum of laccase isozymes from *T. hirsuta* AH28-2

The laccase isozymes from *T. hirsuta* AH28-2 were recombinantly expressed in *Pichia pastoris*, purified, and characterized using 13 lignin model compounds as the substrates (Fig. S5, Tables S1 and S2). Among the recombinant laccases tested, rLacF exhibited the broadest substrate spectrum, demonstrating activity toward 11 compounds. In comparison, rLacA showed activity toward 10 compounds. A notable difference was observed among the other laccases. Specifically, rLacC displayed activity toward nine compounds, while rLacB and rLacE demonstrated activity with seven and six compounds, respectively. Consistent with the substrate screening results, rLacF demonstrated a redox potential of 730 mV, which is higher than that of rLacA at 680 mV under the same testing conditions (Table S3). In contrast, the redox potentials of the other four laccases ranged from 500 to 620 mV and were significantly lower than that of rLacF.

### Preference for lignin oxidation by laccases in different evolutionary branches

**Fig 2:**
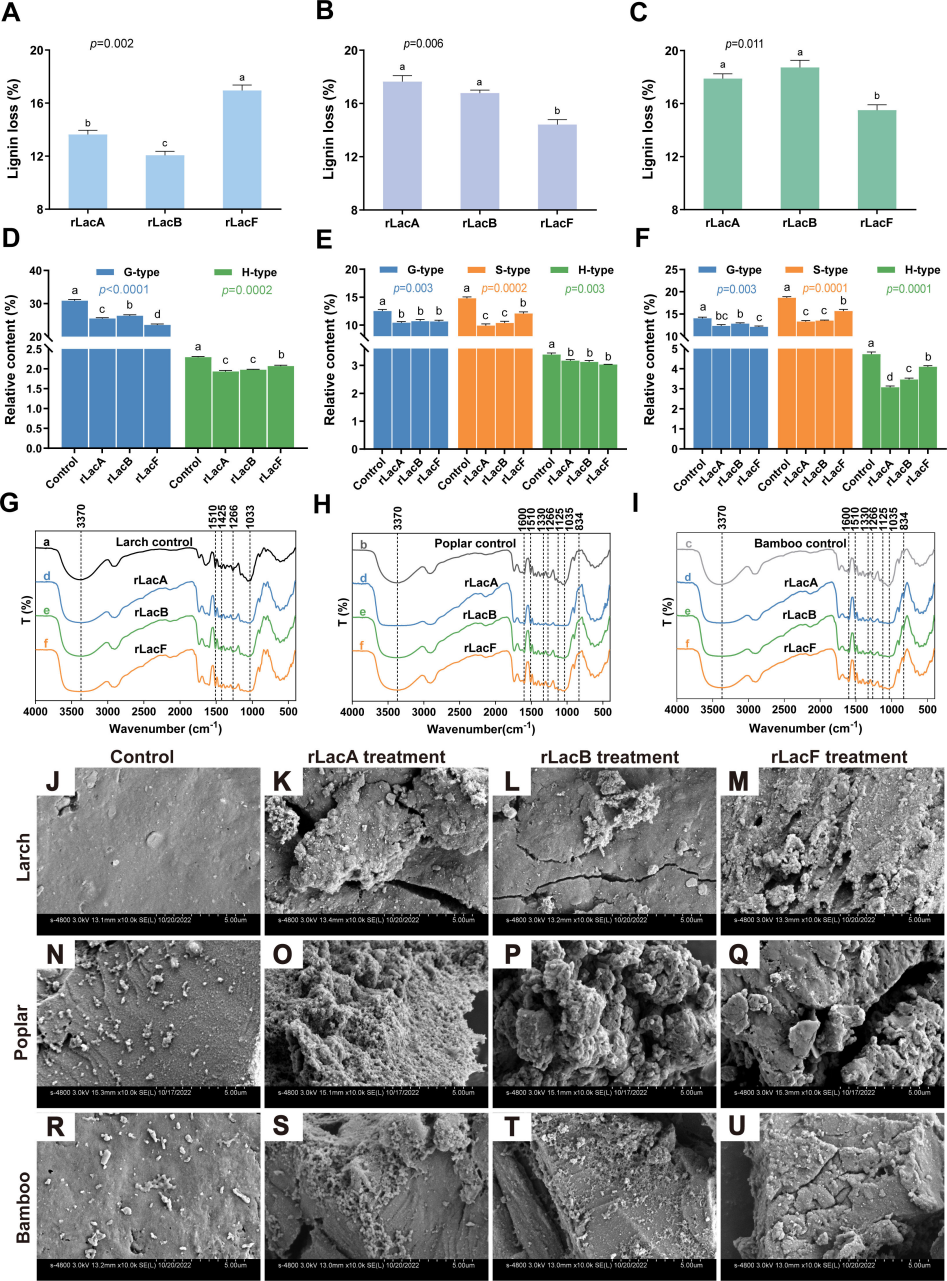
Analysis of lignin oxidation preference of laccases in different evolutionary branches. Efficiency of larch (**A**), poplar (**B**), and bamboo (**C**) lignin oxidation by recombinant laccases. Relative contents of pyrolysis products of larch (**D**), poplar (**E**), and bamboo (**F**) lignin after recombinant laccase treatment. Fourier transform infrared (FTIR) spectra of larch (**G**), poplar (**H**), and bamboo (**I**) oxidized by laccases. Scanning electron microscopy photomicrograph of the surface of larch (**J–M**), poplar (**N–Q**), and bamboo (**R–U**) samples. A representative sample, with weight loss equivalent to the group mean, was selected from the enzyme-treated specimens for FTIR and SEM analysis. The samples were dried under vacuum conditions to obtain a fine powder. SEM analysis was performed at a magnification of ×10,000, and micrographs from multiple regions were acquired to thoroughly evaluate the effect of laccases on the morphological and physical characteristics of wood samples. Representative images were shown. Data are presented as means ± standard deviation (*n* = 3). Different letters indicate a significant difference at *P* < 0.05 according to Duncan’s multiple comparison.

rLacA, rLacB, and rLacF from different branches were selected to investigate their preference for lignin oxidation using larch (gymnosperm), poplar (angiosperm), and bamboo (angiosperm) as the substrates. Incubation of rLacF led to a decrease in the lignin content of larch by 17.0%, higher than the losses observed with rLacA (13.6%) and rLacB (12.1%), respectively (*P* < 0.05) (Fig. 2A). In comparison, the lignin loss of poplar treated with rLacA and rLacB was 17.6% and 16.8%, respectively, both of which were higher than that of rLacF (14.4%) (*P* < 0.05) (Fig. 2B). Similar degradation patterns were observed in bamboo, with laccase-mediated lignin loss rates of 17.9%, 18.7%, and 15.5% corresponding to rLacA, rLacB, and rLacF, respectively (Fig. 2C). Compared with the control group, a significant decrease in the relative abundance of poplar lignin pyrolysis products was observed following distinct laccase treatments (*P* < 0.05) (Fig. 2D through F). The finding indicated that laccase treatments could partially oxidize lignin from different woods, which was consistent with the trends observed in lignin loss rates.

Similar pyrolysis products were obtained after different laccase treatments for each biomass sample; however, the relative abundance of each constituent varied significantly. Specifically, the relative contents of the G-type unit of larch lignin after rLacA and rLacB treatment were 25.48% and 26.31%, but decreased to 23.55% for rLacF (*P* < 0.05) (Fig. 2D; Table S4), indicating rLacF exhibited a more effective oxidation on the G-type units of larch lignin. Conversely, when poplar was utilized as a substrate, the relative contents of S-type units in the groups treated with rLacA and rLacB were measured at 9.91% or 10.37%, respectively, which were significantly lower than those observed in the group treated with rLacF (12.06%, *P* < 0.05) (Fig. 2E). Furthermore, it was evident that the S-type unit/G-type unit (S/G) ratios of the poplar in both the rLacA and rLacB treatment groups were lower than those in the rLacF treatment group (Table S5), suggesting that the laccase rLacA and rLacB preferentially degrade S-type lignin within the poplar. Similarly, when bamboo served as a substrate, treatments with both rLacA and rLacB demonstrated an analogous pattern of oxidation preference (Fig. 2F; Table S6).

Fourier transform infrared spectroscopy (FTIR) analysis of untreated wood samples and those subjected to various laccase treatments showed that the larch sample displayed typical characteristics associated with coniferous woods, where the intensity of the absorption peak at approximately 1,266 cm⁻¹ was significantly greater than that at 1,220 cm⁻¹ (Fig. 2G). In contrast, the spectra for poplar and bamboo samples exhibited a more pronounced band at 1,220 cm⁻¹, which was indicative of features characteristic of deciduous species (Fig. 2H and I). Although the wood spectra across different laccase treatment groups were overall similar, differences were observed at 1,600–1,000 cm^−1^. In the case of larch, lignin treated with laccases exhibited diminished aromatic C-H bending and C=O stretching in the guaiacyl unit at 1,033 and 1,266 cm^−1^, respectively, when compared to the control group. The broad peak observed at 3,370 cm^−1^ corresponded to the O-H stretching vibration. Additionally, an aromatic skeletal vibration was noted at 1,510 cm^−1^, while a peak at 1,425 cm^−1^ represented the aromatic skeletal vibration combined with the C-H in-plane deformation in -OCH_3_. Notably, the laccase-modified larch lignin demonstrated higher relative transmittances than that of the control group in O-H stretching vibration, C=O stretching vibration, and C-H in-plane deformation in -OCH_3_ (Table 1). This observation suggested that the G-type units in larch lignin were oxidized to varying degrees, with laccase performance ranking as rLacF > rLacB > rLacA. In contrast, significant weakening of absorption peaks at 1,125 and 1,035 cm^−1^ was observed in the spectra of laccase-modified poplar and bamboo lignins (Fig. 2H and I), suggesting demethylation of methoxyl groups surrounding syringyl and guaiacyl rings.

**TABLE 1:** Infrared spectral relative transmittance^*a*^ of lignin in control and laccase treatment groups

Wood	Wavenumber (cm^−1^)	Relative transmittance			
		Control	rLacA treatment	rLacB treatment	rLacF treatment
Larch	3,370	0.09	0.19	0.18	0.22
	1,510	1.00	1.00	1.00	1.00
	1,425	0.70	0.76	0.79	0.82
	1,266	0.52	0.61	0.63	0.70
	1,033	0.11	0.18	0.19	0.21
Poplar	1,600	0.89	0.96	0.95	0.94
	1,510	1.00	1.00	1.00	1.00
	1,330	0.46	0.85	0.77	0.67
	1,266	0.33	0.78	0.71	0.67
	1,125	0.12	0.52	0.29	0.25
	834	2.11	15.85	4.93	3.60
Bamboo	1,600	0.74	0.89	0.85	0.84
	1,510	1.00	1.00	1.00	1.00
	1,330	0.60	0.65	0.65	0.61
	1,266	0.45	0.54	0.53	0.48
	1,125	0.20	0.25	0.26	0.22
	834	2.87	4.80	4.68	4.37

^*a*^ The relative transmittance of each peak was calculated with the transmittance of the aromatic skeletal vibration at 1,510 cm^−1^ as the standard.

As illustrated in Table S7, a peak appearing at 1,600 cm⁻¹ can be attributed to C=O vibrations. The spectra from hardwoods displayed a band around 1,330 cm⁻¹, characteristic of syringyl ring breathing coupled with C-O stretching vibrations. Furthermore, a vibration detected at 834 cm⁻¹ was attributed to the C-H out-of-plane in positions 2 and 6 of S units. The poplar and bamboo lignins treated with laccases showed higher relative transmittance than untreated wood samples in the peaks at 1,600, 1,330, 1,125, and 834 cm^−1^ (Table 1). This enhancement may result from exposure (modification/degradation) of functional groups associated with syringyl units during laccase action. Thus, the oxidation of syringyl units within deciduous wood by laccases rLacA and rLacB was superior to that observed using rLacF. In accordance with this conclusion, cell walls of non-treated woods showed an intact morphology (Fig. 2J, N, and R). In contrast, the larch sample treated with rLacF displayed a slightly rougher and more uneven surface compared to those treated with rLacA or rLacB (Fig. 2K through M). Conversely, in angiosperms such as poplar or bamboo, samples treated with rLacA and rLacB exhibited increased cracks and pore formation on their surfaces compared with rLacF treatment (Fig. 2O through Q and S through U), which may be attributed to varying degrees of lignin removal.

### Analysis of lignin-derived products degraded by laccase isozymes

**Fig 3:**
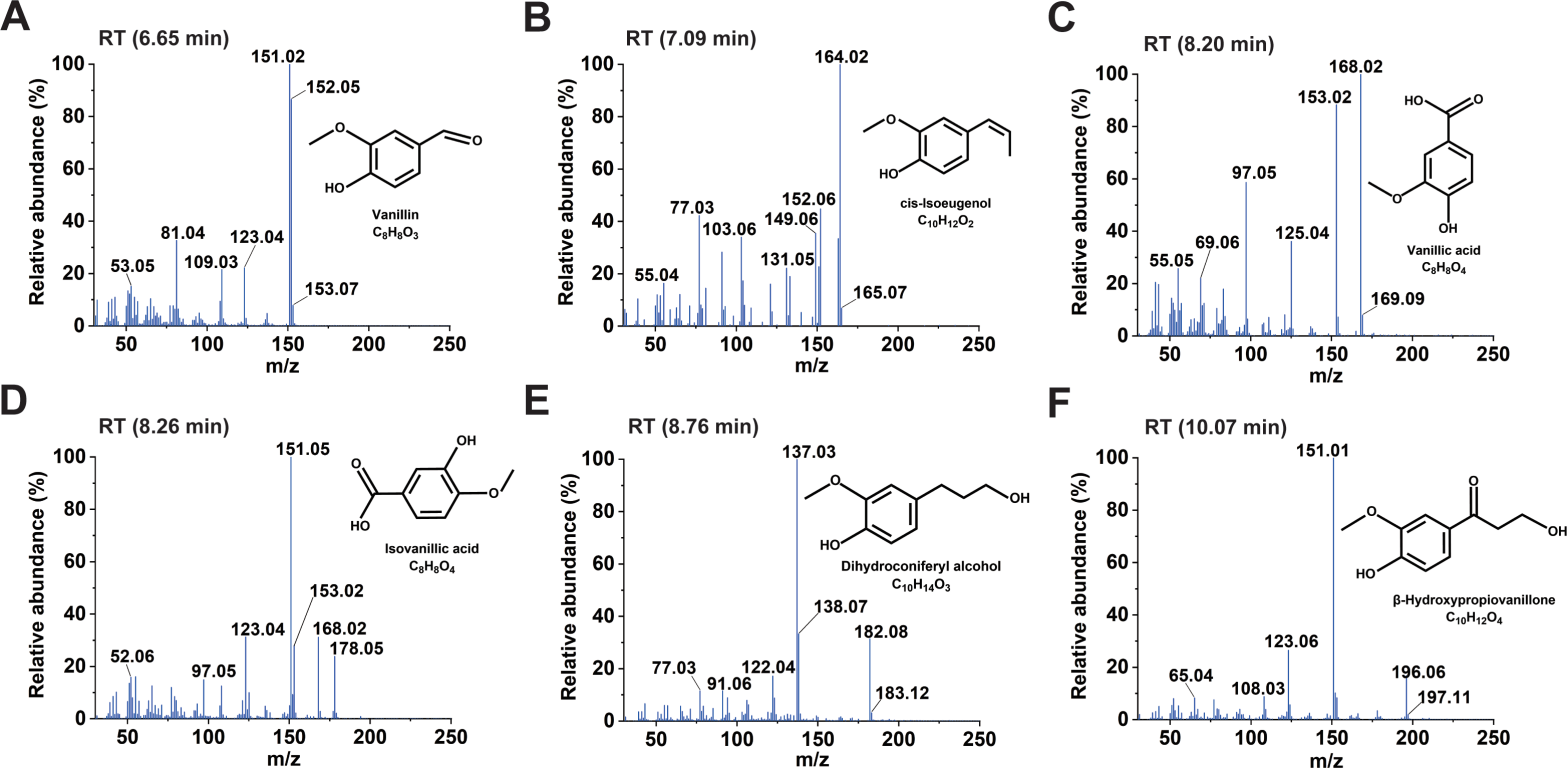
Mass spectrum analysis of larch lignin degradation products generated by laccase rLacF. (**A**) Vanillin; (**B**) *cis*-isoeugenol; (**C**) vanillic acid; (**D**) isovanillic acid; (**E**) dihydroconiferylalcohol; and (**F**) *β*-hydroxypropiovanillone.

**Fig 4:**
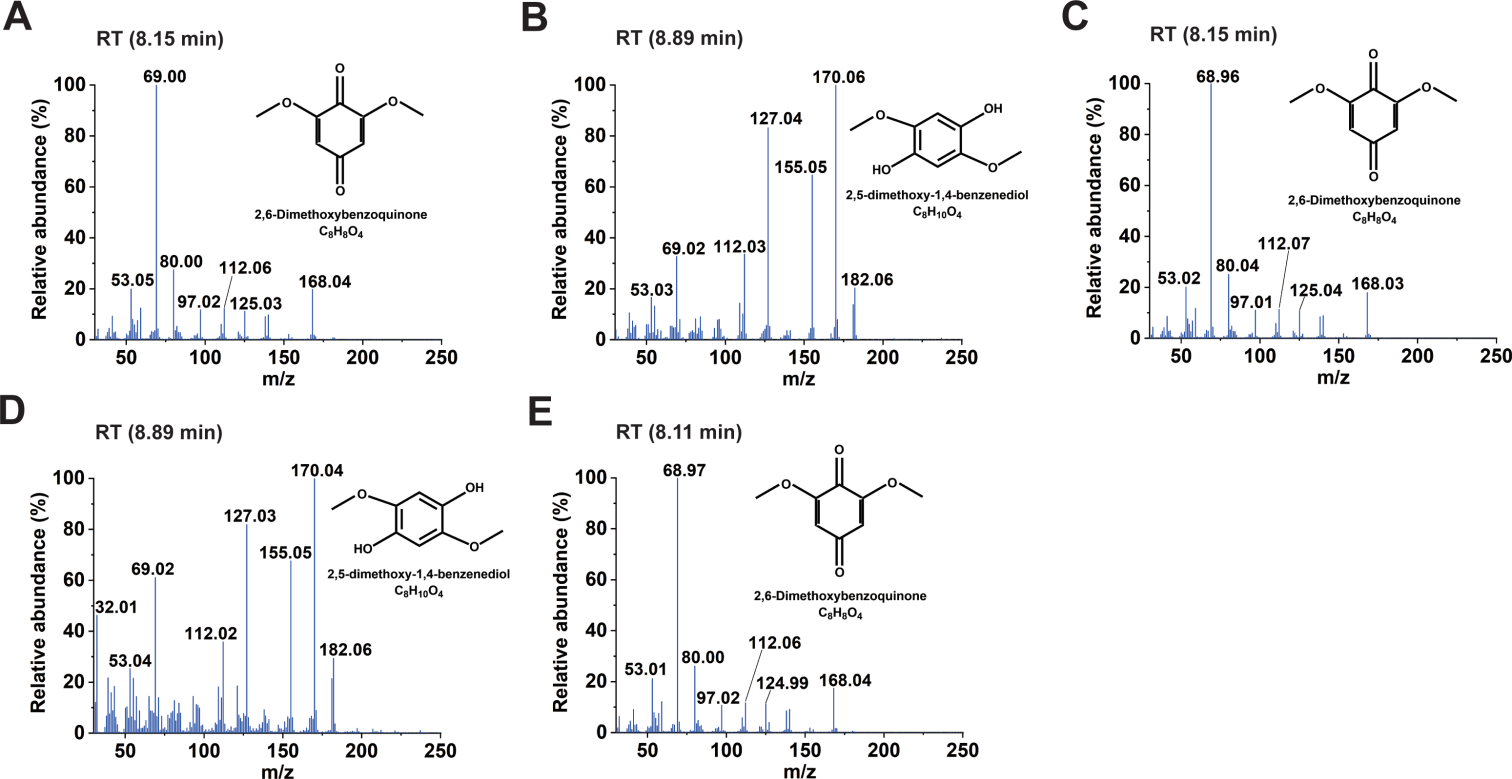
Mass spectrum analysis of bamboo lignin degradation products generated by recombinant laccases (rLacA, rLacB, and rLacF). (**A**) 2,6-Dimethoxybenzoquinone and (**B**) 2,5-dimethoxy-1,4-benzenediol were degradation products of rLacA; (**C**) 2,6-dimethoxybenzoquinone and (**D**) 2,5-dimethoxy-1,4-benzenediol were degradation products of rLacB; (**E**) 2,6-dimethoxybenzoquinone was degradation product of rLacF.

Lignin degradation products from different types of wood, catalyzed by three laccase isozymes (rLacA, rLacB, and rLacF), were identified using gas chromatography-mass spectrometer (GC-MS) analysis. When larch wood was used as the substrate, the degradation products predominantly consisted of guaiacyl derivatives. rLacF exhibited the highest catalytic performance, yielding six compounds: vanillin, *cis*-isoeugenol, vanillic acid, isovanillic acid, dihydroconiferyl alcohol, and *β*-hydroxypropiovanillone (Fig. 3). rLacA generated four products: 2-methoxy-4-vinylphenol, vanillin, isoeugenol, and dihydroconiferyl alcohol (Fig. S6). In contrast, rLacB demonstrated the weakest activity, producing only vanillin, dihydroconiferyl alcohol, and *β*-hydroxypropiovanillone (Fig. S7). In the case of bamboo wood, the product profile was dominated by syringyl derivatives. Both 2,6-dimethoxybenzoquinone and 2,5-dimethoxy-1,4-benzenediol were detected in the rLacA and rLacB groups, whereas only 2,6-dimethoxybenzoquinone was found in the rLacF group (Fig. 4). A similar pattern was observed with poplar: both compounds were detected in the rLacA group, while only 2,6-dimethoxybenzoquinone was present in the rLacB and rLacF groups (Fig. S8). These results demonstrated that laccases could contribute to oxidizing lignin to release distinct phenolic compounds. Specifically, rLacF was more proficient in generating G-type derivatives from larch lignin, whereas rLacA tended to produce more S-type derivatives from poplar and bamboo lignin.

### Lignin-degrading preferences in *T. hirsuta* AH28-2 *lacA*, *lacB*, and *lacF* silencing transformants

**Fig 5:**
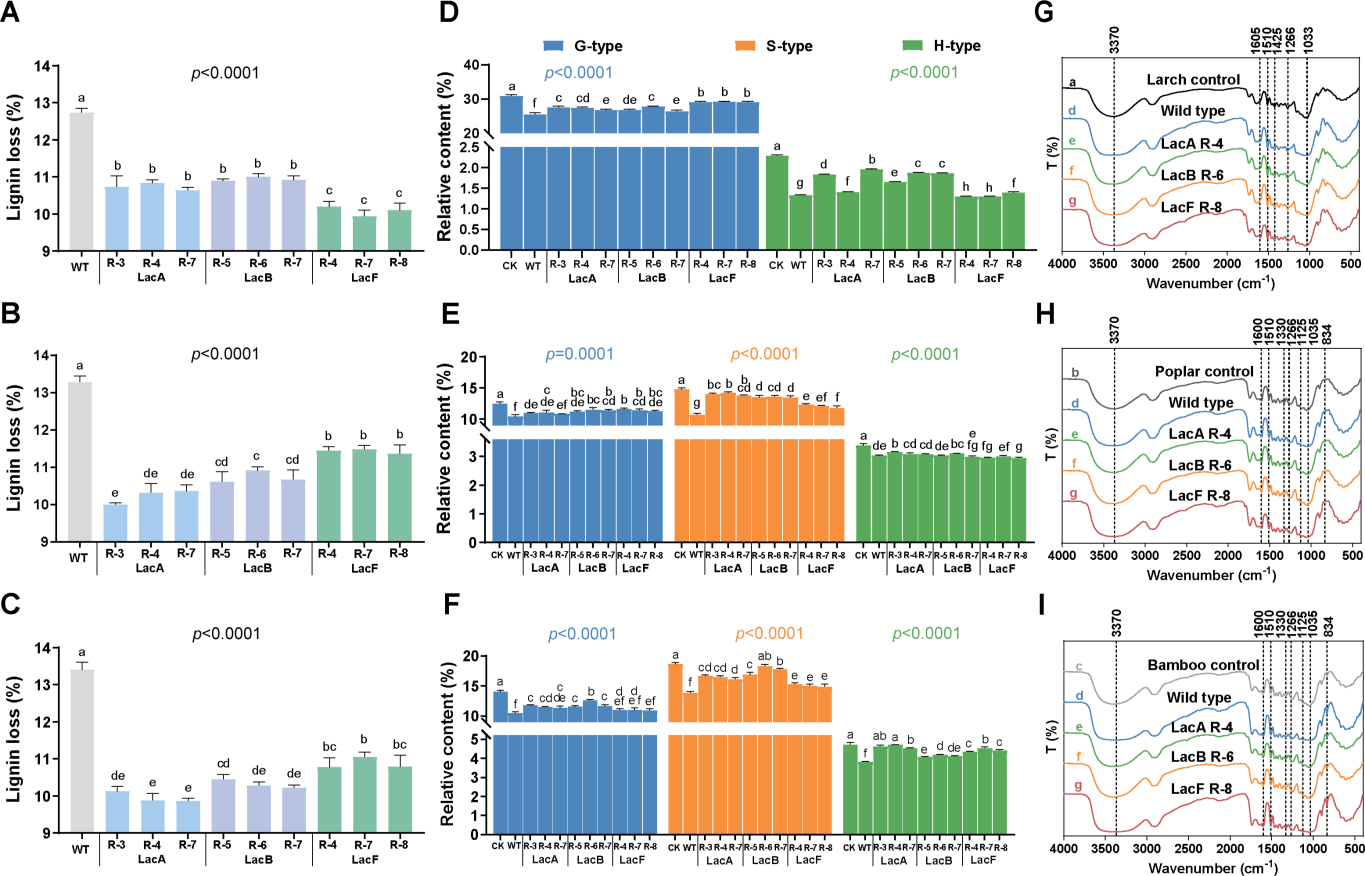
Analysis of lignin degradation preference of gene-silenced transformants. Analysis of lignin loss after degradation of larch (**A**), poplar (**B**), and bamboo (**C**) by gene-silenced transformants. Analysis of pyrolysis products after degradation of larch (**D**), poplar (**E**), and bamboo (**F**) by gene-silenced transformants. Analysis of FTIR spectra after degradation of larch (**G**), poplar (**H**), and bamboo (**I**) by gene-silenced transformants. Data are presented as means ± standard deviation (*n* = 3). Different letters indicate a significant difference at *P* < 0.05 according to Duncan’s multiple comparison.

Nine silencing transformants were successfully obtained, as confirmed by qRT-PCR and laccase activity analysis. These included LacA R-3, LacA R-4, and LacA R-7 for *lacA* silencing; LacB R-5, LacB R-6, and LacB R-7 for *lacB* silencing; and LacF R-4, LacF R-7, and LacF R-8 for *lacF* silencing (Fig. S9). To further investigate the lignin-degrading preferences in *T. hirsuta* AH28-2 *lacA*, *lacB*, and *lacF* silencing transformants, both the wild-type (WT) strain and silencing transformants were cultivated on various lignocellulosic substrates. In terms of lignin degradation in wood, the observed lignin loss (Fig. 5A through C) was significantly lower in all silencing transformants compared with WT, suggesting that the silencing transformants exhibited a reduced capacity for lignin degradation in wood relative to WT. The lignin loss in larch for *lacA*- and *lacB*-silenced transformants was higher than that observed for *lacF*-silenced transformants (Fig. 5A). Conversely, when poplar or bamboo were used as substrates, the lignin loss associated with *lacA* and *lacB* silencing was less than that observed with *lacF* silencing (Fig. 5B and C). These results suggested that *lacF* silencing markedly diminished the lignin-degrading ability of *T. hirsuta* AH28-2 on larch; meanwhile, both *lacA* and *lacB* silencing impaired its ability to degrade lignin from poplar or bamboo.

Among all the silencing transformants, inoculation with *lacF*-silenced transformants resulted in the highest total peak areas of G units within larch lignin (Fig. 5D; Table S8). Additionally, higher total peak areas of S units within poplar or bamboo lignin were identified specifically for both *lacA*- and *lacB*-silenced transformants (Fig. 5E and F). These results indicated a diminished degradation capability concerning G units within larch lignin after *lacF* silencing; conversely, the degradation abilities regarding S units within poplar or bamboo lignins were also reduced after *lacA* silencing or *lacB* silencing. Moreover, as expected, the S/G ratios for poplar and bamboo treated with *lacF*-silenced transformants were found to be lower than those of *lacA*- and *lacB*-silenced transformant treatment groups (Tables S9 and S10). The variation in the total peak areas of lignin units among the three *lacF* silencing transformants (LacF R-4, LacF R-7, and LacF R-8) during biodegradation clearly indicated a more reduced degradation of G-type than S-type units in polar-derived lignin. The observed decrease in the S/G ratio in the residue lignin supported this conclusion, which was recorded at 1.00, 0.95, and 0.96 for each respective transformant. Furthermore, when bamboo was used as a substrate, the *lacF*-silenced transformants also exhibited similar patterns of degradation.

FTIR analysis revealed that the intensity of the absorption peak at 1,033/1,035 cm^−1^ for wood samples treated with *lacA*-silenced transformants was significantly diminished following fungal degradation (Fig. 5G through I). Tables S11 to S13 present the data on the relative transmittance of each peak observed in treated larch, poplar, and bamboo woods. The broad peak at 3,370 cm^−1^ corresponded to O-H stretching vibrations. Compared with control samples, both WT and all silencing transformant-treated woods exhibited higher relative transmittance for O-H stretching vibration, aromatic skeletal vibrations plus C=O stretching, and C-H in-plane deformation of -OCH_3_. In the case of larch lignin, treatment groups with LacA R-4 and LacB R-6 demonstrated higher relative transmittance than the treatment group with LacF R-8 in these functional groups. Conversely, for poplar and bamboo lignin, the treatment group with LacF R-8 showed higher relative transmittance than those treated with LacA R-4 or LacB R-6, specifically within the functional groups associated with syringyl units. These results indicated that the capacity of LacA R-4 and LacB R-6 to degrade G-type units in larch lignin was superior to that of LacF R-8. In contrast, LacF R-8 exhibited a greater ability to degrade S-type units in poplar and bamboo lignin compared to both LacA R-4 and LacB R-6.

To determine whether the diminished lignin-degrading capability was attributed to a reduction in the production of extracellular enzymes, the fungal biomass and extracellular ligninolytic enzyme activity were examined. As illustrated in Fig. S10, the ergosterol contents in *lacA*-, *lacB*-, and *lacF*-silenced transformants were significantly lower compared to those of the WT strain. This suggested that the silencing transformants colonized natural lignocellulose at a slow rate, which was consistent with their reduced lignin-degrading ability observed on larch, poplar, and bamboo. In terms of ligninolytic enzymes, laccase activities in the silencing transformants were markedly lower than those of WT (Fig. S11A through C). Native PAGE analysis further revealed a pronounced decrease in LacA and LacB expression levels within *lacA*- and *lacB*-silenced transformants, respectively (Fig. S11D through F). Notably, *lacF* silencing resulted in a significant downregulation of *lacF* transcription. Concurrently, both *lacA* and *lacB* silencing also led to decreased expression levels of LacA and LacB (Fig. S11G through I). Additionally, two other types of enzyme activity, including MnP and LiP, were assessed (Fig. S12). They exhibited similar trends to laccase activity, which may be related to the observed decline in fungal biomass among the silencing transformants.

The micromorphological characteristics of degraded woods after inoculation with WT and silencing transformants exhibited significant differences (Fig. S13). Notably, a higher number of broken fragments and pores were observed in the wood samples inoculated with *T. hirsuta* AH28-2 WT strain (Fig. S13A, E, and I). Larch samples inoculated with silencing transformants such as LacA R-4 or LacB R-6 displayed similar degradation morphology; however, this was less pronounced compared to those treated with WT (Fig. S13B and C). In contrast, the larch sample colonized by LacF R-8 maintained a relatively flat plant cell wall structure while containing some larger fragments (Fig. S13D). When comparing the effects of LacF R-8 on poplar or bamboo samples to those affected by LacA R-4 and LacB R-6, it was evident that the former exhibited a collapsed structure characterized by smaller fragments (Fig. S13F through H and J through L). The surface alterations observed in the wood samples further corroborated the varying preferences of different silencing transformants for degrading larch, poplar, and bamboo.

### Evolution of the laccase genes from the fungi of the Agaricomycetes class

**Fig 6:**
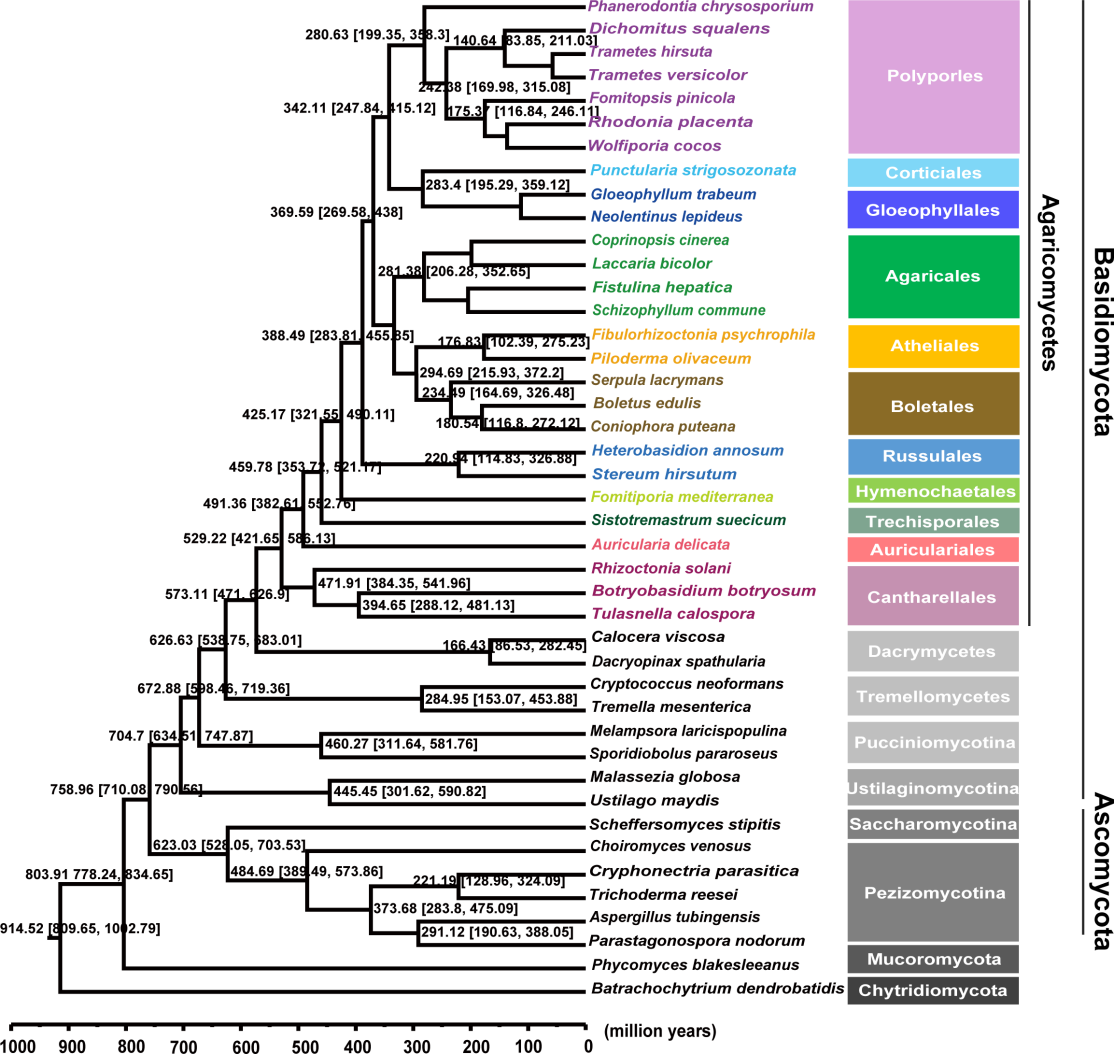
Evolutionary tree with divergent evolutionary time. Mean ages for the selected nodes are provided along with their 95% highest posterior density ranges.

The evolutionary history of laccase genes from the diverse fungi was further analyzed to investigate the universality of laccase evolution. A total of 43 fungal species were selected and categorized into four distinct ecological habitats. Molecular clock analysis indicates that Agaricomycetes emerged approximately 250 million years ago during the Early Triassic period and subsequently underwent independent evolution across various taxa (Fig. 6). Furthermore, a rapid diversification event occurred within Agaricomycetes during the early Cretaceous period, around 140 million years ago. This diversification persisted until the Late Cretaceous period, approximately 60 million years ago, primarily involving members of the Polyporales group. To elucidate the evolutionary history, a phylogenetic tree of laccase genes among these fungi was constructed using the maximum likelihood method based solely on the alignment of the laccase amino acid sequences (Fig. S14). The reconciliation analysis between gene tree and species tree indicated that phylogenetic relationships among these laccase genes within subclades mirrored those observed among corresponding fungal species; however, they did not strictly conform to species evolution patterns.

### Analysis of lignin oxidation through laccase evolution

**Fig 7:**
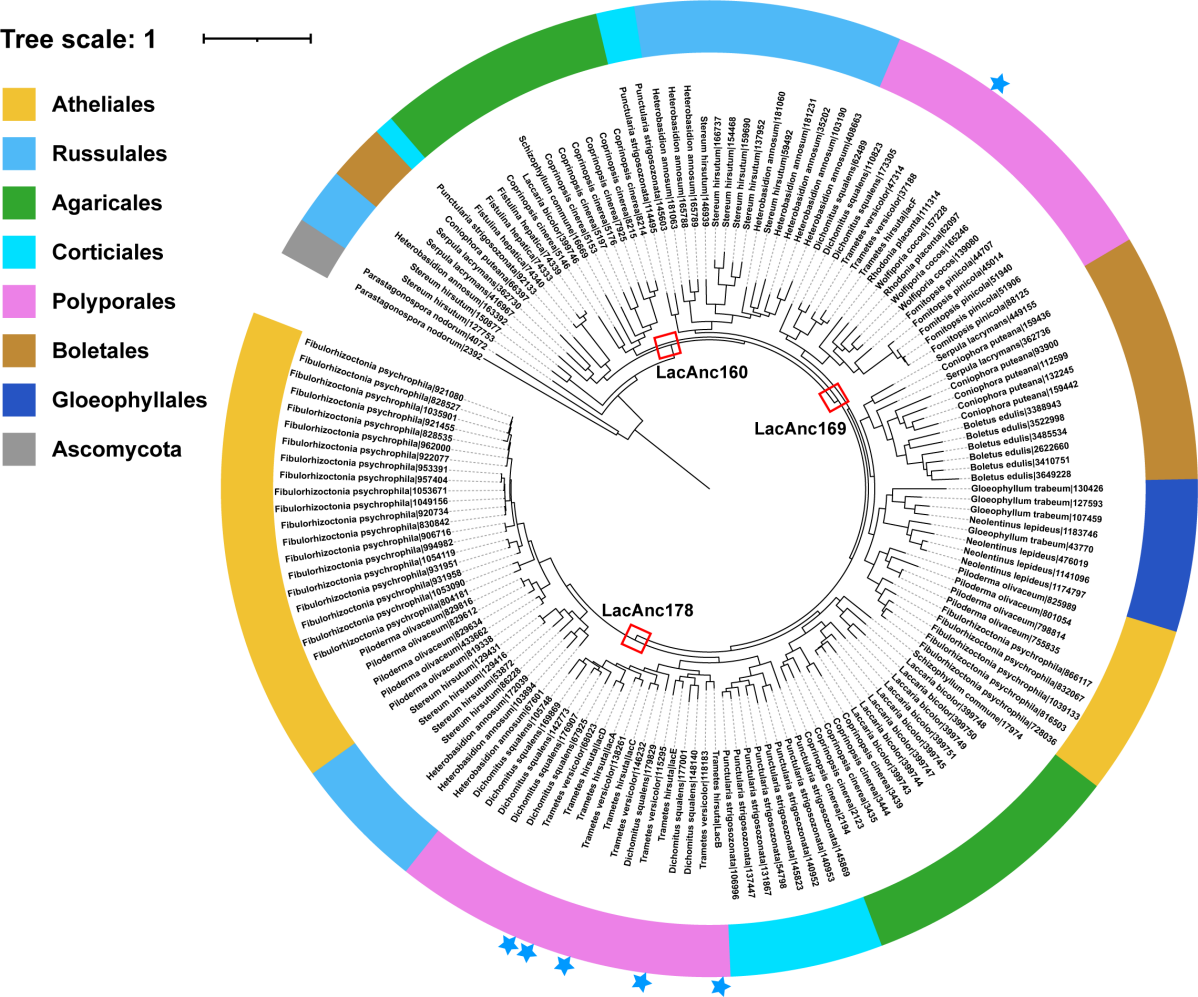
The phylogenetic tree based on the amino acid sequence of laccase from the fungi of the Agaricomycetes class. The nodes whose sequences were selected for resurrection are depicted as red squares.

**Fig 8:**
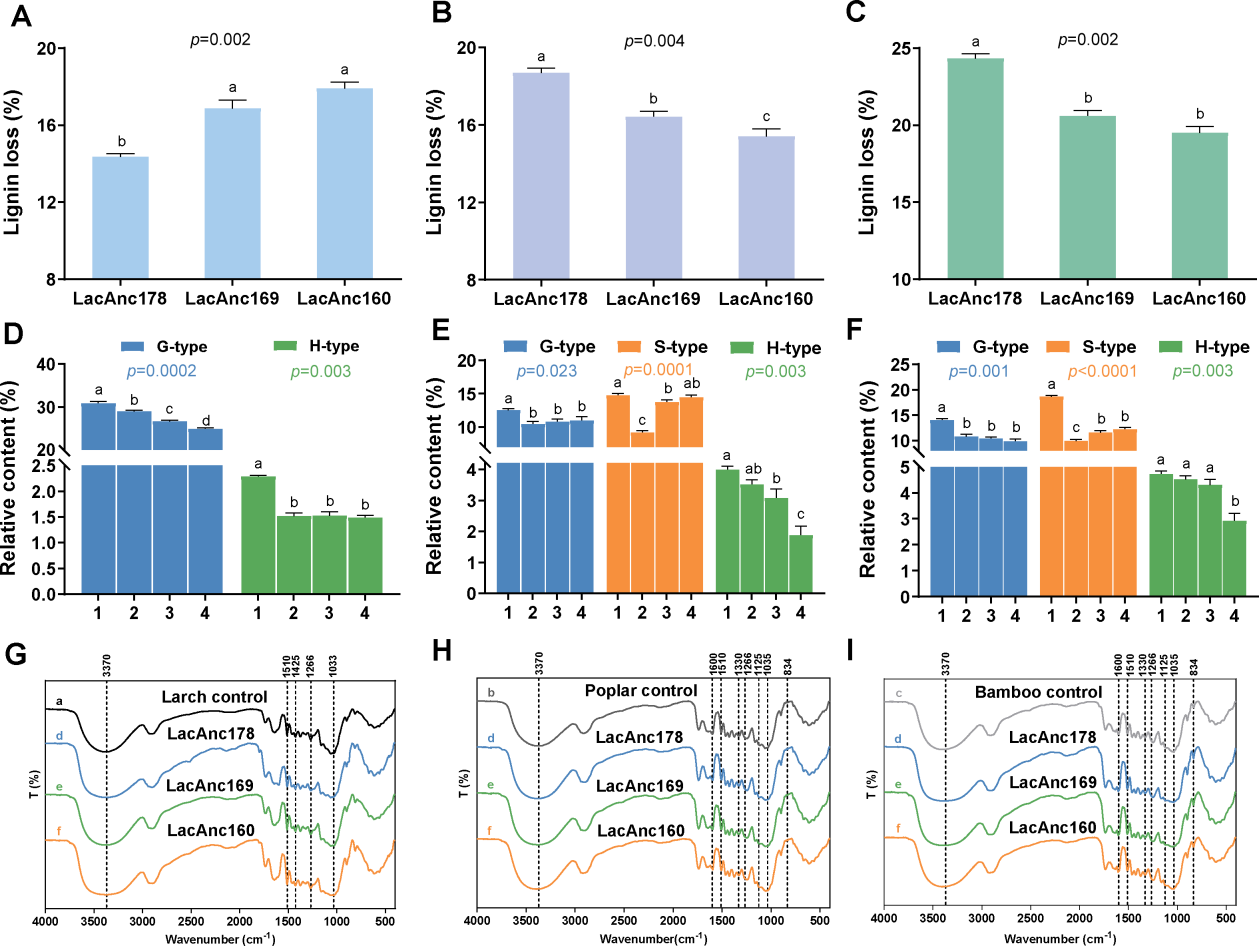
Analysis of lignin oxidation preference of ancestral laccases. Efficiency of larch (**A**), poplar (**B**), and bamboo (**C**) lignin oxidation by ancestral laccases. Relative contents of pyrolysis products of larch (**D**), poplar (**E**), and bamboo (**F**) lignin after ancestral laccase treatment. FTIR spectra of larch (**G**), poplar (**H**), and bamboo (**I**) oxidized by ancestral laccases. Lanes: 1: control; 2: LacAnc178; 3: LacAnc169; and 4: LacAnc160. Data are presented as means ± standard deviation (*n* = 3). Different letters indicate a significant difference at *P* < 0.05 according to Duncan’s multiple comparison.

The laccase genes among these fungi in the Class Agaricomycetes were further analyzed. Three ancestral laccases were reconstructed using PAML, and three of them, including LacAnc160, LacAnc169, and LacAnc178, were subsequently resurrected through expression in *P. pastoris* (Fig. 7). Proteins were purified via ion-exchange chromatography, as shown by SDS-PAGE analysis (Fig. S15). The delignification ability of these ancestral laccases was evaluated using larch, poplar, and bamboo wood as substrates. The addition of LacAnc160 resulted in a 17.91% reduction in lignin content in larch, which was significantly higher than the reductions observed with LacAnc178 and LacAnc169 (*P* < 0.05) (Fig. 8A). In contrast, the lignin degradation efficiencies for poplar treated with LacAnc178 and LacAnc169 were recorded at 18.69% and 16.43%, respectively; both values exceeded that of LacAnc160 (15.42%) (*P* < 0.05) (Fig. 8B). A similar trend in oxidation efficiency was noted for bamboo, paralleling the results obtained from poplar treatment (Fig. 8C). Furthermore, the wood surfaces exhibited distinct fragmentation changes across various samples (Fig. S16). These alterations could be attributed to the differential oxidative performance of lignin mediated by ancestral laccases. Notably, the intensity of these morphological changes corresponded closely to trends observed in lignin loss rates.

A quantitative analysis of the pyrolysis products derived from lignin in the wood samples indicated a reduction in lignin content relative to the control sample that was not treated by enzymes, as well as an assessment of the S/G ratio (Tables S14 to S16; Fig. 8D through F). The treatment of softwood and hardwood with the ancestral laccases revealed similar pyrolysis products between these samples and those from the enzyme-free control. However, notable differences were observed in the relative contents of aromatic pyrolysis products. The performance of larch wood oxidized by ancestral laccases was ranked as LacAnc178 < LacAnc169 < LacAnc160, with relative contents of G-type units recorded at 27.94%, 26.62%, and 26.14%, respectively (Fig. 8D). In poplar wood, integrating the aromatic compounds showed a decrease over evolutionary time, with LacAnc178 producing maximal changes characterized by 9.19% S-type units and 10.45% G-type units (Fig. 8E). Similar results were noted when ancestral laccases catalyzed the oxidation of bamboo (Fig. 8F). Concurrently, there was an overall decrease in the S/G ratio in enzyme-treated lignin throughout laccase evolution (Tables S15 and S16), indicating that during later stages, LacAnc178 exhibited a preference for oxidizing S-type units within angiosperm lignin.

In addition, the FTIR spectra of untreated and ancestral laccase-treated biomasses are shown in Fig. 8G through I. The larch wood treated with LacAnc160 exhibited a higher relative transmittance than LacAnc169 and LacAnc178 at the peaks corresponding to 3,370, 1,425, 1,266, and 1,033 cm^–1^ (Table S17). This observation suggested that LacAnc160 demonstrated a superior preference for oxidizing larch lignin when compared to LacAnc169 and LacAnc178. Conversely, the poplar and bamboo lignin treated with LacAnc169 and LacAnc178 displayed higher relative transmittance than those treated with LacAnc160 at peaks of 1,330, 1,125, and 834 cm^–1^ (Tables S18 and S19). The progressive increase in relative transmittance within the functional groups of syringyl units in hardwood lignin through laccase evolution supported the trend observed in pyrolysis-gas chromatography/mass spectrometry (Py-GC/MS) measurements.

## DISCUSSION

Currently, evolutionary analysis shows that the laccase genes underwent multiple duplications with the Polyporales, proposing that the rapid expansion of laccase genes overlapped with the evolution of plants from gymnosperms to angiosperms (18). However, currently, no research has demonstrated a direct relationship between the evolution of lignin and the replication and evolution of fungal laccase. In this study, both *in vitro* and *in vivo* analyses showed that the earlier-emerging laccase isozyme LacF from *T. hirsuta* AH28-2 preferentially oxidized G-type units present in softwood lignin. In contrast, the later-emerging laccase isozymes LacA and LacB exhibited a preference for S-type units found in hardwood lignin. Furthermore, laccases from various evolutionary nodes exhibited distinct preferences for lignin oxidation, indicating that the evolution of fungal laccase may be associated with the emergence of S-type units in angiosperm lignin.

Fungi have likely evolved sophisticated mechanisms to sense aromatic compounds and subsequently respond in a timely manner by differentially secreting laccase isozymes (31). Extensive studies indicated that lignin-derived phenolic monomers can efficiently trigger the expression of several specific isozymes under both liquid and solid cultivation conditions (31, 34–36). The structurally closely related aromatic compounds appeared to have different effects on both laccase activity levels and gene transcription in the ligninolytic basidiomycete *Trametes* sp. I-62 (37). In this study, the secretomes derived from *T. hirsuta* AH28-2 treated with syringyl compounds demonstrated enhanced enzymatic activity and an increased abundance of laccase protein (Fig. 1B and C). Furthermore, notable differences were observed in the transcriptional patterns of six laccase genes in *T. hirsuta* AH28-2 following exposure to various monolignols (Fig. 1D and E). Among them, *lacA* can be distinguished as the predominant constitutively expressed laccase gene (34). For the non-constitutive genes, although syringyl compounds did influence the changes in *lacF* expression, the fluctuations in gene expression were significantly less pronounced compared to those observed for other non-constitutively expressed laccase genes (*lacB*, *lacC*, *lacD*, and *lacE*). Similar results were reported by Moiseenko et al. (34), whose study revealed that the expression response of the laccase multigene family exhibited a mosaic pattern, emphasizing the subfunctionalization of expression within this gene family. In addition, the comparison of substrate specificity tests for the laccase isozymes of *T. hirsuta* AH28-2 (Table S2) confirmed the correlation between the variations in the properties of different isozymes and the phylogenetic proximity of their corresponding laccase genes. The laccase isozymes LacA, LacC, and LacD, which were classified within the same evolutionary clade, exhibited highly similar substrate specificities. Furthermore, both the isozymes LacB and LacE from another evolutionary group demonstrated a limited range of substrate oxidation. It could be inferred that laccase isozymes residing on the same evolutionary branch may perform analogous functions within the fungus (18). As reported by Moiseenko et al. (38), the multigenicity of laccases should be explained by the broad spectrum of their physiological functions rather than the utilization of all of the genes in one particular process.

In nature, microorganisms have evolved different enzymatic and non-enzymatic strategies to utilize the plentiful plant material (39). In the context of wood biodegradation, iron-driven Fenton reactions are considered to contribute to the non-enzymatic degradation processes (40). Such non-enzymatic oxidative and reductive reactions are particularly important during the initial stages of decay because fungal enzymes are too large to penetrate the intact cell wall of wood (41). Generally, it was thought that the ability of white-rot fungi to degrade lignin primarily relied on a range of LMEs, including laccase, MnP, and LiP (13, 39). *Trametes* species, as a typical representative of white-rot fungi, can grow on various genera of hardwood trees (oak and prunus) and some conifers (fir and larch) and have been observed to degrade lignin in wood at a faster rate than cellulose (42). Here, compared with the WT strain, the constructed gene-silenced *T. hirsuta* AH28-2 transformants exhibited a reduced lignin-degrading capability (Fig. 5). Through the lignin degradation process, the activities of MnP and LiP were sustained at a low level, which may be attributed to the relatively low fungal biomass observed in the silenced transformant treatment group (Fig. S10 and S12). Interestingly, *lacF*-silenced transformants could still effectively degrade the lignin of poplar and bamboo (Fig. 5), indicating that laccases were likely involved in the degradation of lignin by *T. hirsuta*. Thus, this study presents evidence that the LMEs, especially laccases, might contribute to the actual degradation of natural lignin in plant cell walls. In addition, the lignin loss rate observed in *lacA*- and *lacB*-silenced transformants was higher than that of *lacF*-silenced transformants during larch degradation (Fig. 5A). Considering that *lacA* and *lacB* emerged from the gene *lacF*, it was plausible that each laccase isozyme in distinct subclades did not play a unique role. On the contrary, their function in lignin degradation appeared to be redundant.

In this study, the compounds generated from laccase-mediated lignin degradation were found to be predominantly monophenolic and polyphenolic in nature. The absence of downstream small-molecular-weight compounds indicated that laccase alone was insufficient to cleave the aromatic C-C bonds within the benzene ring to enable subsequent degradation steps. The primary degradation pathway involved laccase-mediated oxidation of lignin to form phenoxy radicals, which facilitated the cleavage of C*α*-C*β* bonds, leading to lignin depolymerization and the release of phenolic units (43, 44). This was consistent with the work of Zhang et al. (45), who demonstrated that a novel laccase, LacZ1, could break the *β*-O-4 bond, *β*-5 bond, and *β-β* bond in lignin structure, as evidenced by GC-MS analysis of the products. Additionally, the laccase-generated radicals may trigger non-enzymatic chain reactions. For example, some researchers have confirmed that the Fenton-like reaction can generate highly reactive hydroxyl radicals (·OH), which are known to cause surface oxidation and attack the lignin polymer (46, 47). These intense oxidative conditions could indirectly contribute to degradation by cleaving side chains from lignin precursors, resulting in the formation of phenolic compounds.

Based on Py-GC/MS assays, it could be seen that the laccase rLacF exhibited a higher affinity for the oxidation of G-type lignin in gymnosperms. In contrast, rLacA and rLacB treatment resulted in an increased loss of S/G ratio within the poplar and bamboo lignin, indicating that both rLacA and rLacB showed a marked preference for the degradation of S-type lignin in angiosperm (Tables S4 to S6). Furthermore, FTIR analysis demonstrated the alterations of functional groups in lignin following laccase treatment (Table 1). Similar phenotypes have also been reported (48, 49). These results suggest that laccase plays a crucial role in oxidizing phenolic hydroxyl groups in lignin, leading to the formation of new unconjugated carbonyl groups and facilitating the demethylation of methoxyl groups (49). These findings related to hardwood lignin indicate that the preference for lignin oxidation has evolved to be more pronounced in laccase isozymes from specific evolutionary branches.

The gene-silenced transformants exhibited disparate effects on the overall composition of lignin subunits, which indicated that all lignin units were targeted in the lignin degradation process. After *lacF* silencing, *T. hirsuta* AH28-2 exhibited a decreased capacity for degrading G-type units within softwood and a more reduced capacity for degrading G-type than S-type units within hardwood (Fig. 5; Tables S8 to S10). Nevertheless, the preferential degradation of S- over G-type units was decreased when treated with *lacA*- or *lacB*-silenced transformants (Tables S9 and S10). Preferential degradation of S-type units demonstrated that the fungi degrade mainly *β*-O-4-ether linkages, which are the predominant linkages found in S-type units (50). It has been shown that the white-rot fungi *Pleurotus eryngii* and *Lentinula edodes* exhibit a preference for the oxidative cleavage of S-type substructure in wheat straw (50, 51). Collectively, the observed differences in the degradation of various types of lignin support the finding that LacF primarily contributes to oxidizing G-type units found in gymnosperm lignin, whereas LacA and LacB primarily contribute to oxidizing S-type units present in angiosperm lignin. While the data strongly suggest a direct role for these laccases, the observed effects could be partially influenced by variations in fungal biomass and the activity of other peroxidases (e.g., MnP/LiP) in the silenced strains. The observed changes in substrate preference may reflect functional adaptations of the laccase isozyme to rapidly changing environmental conditions (18).

A gene-tree/species-tree reconciliation analysis was performed to gain insight into the evolutionary history of the laccase genes from the wood-decaying fungi. Molecular clock analyses suggested that Agaricomycetes originated approximately 220–300 million years ago during the Early Triassic period (Fig. 6). This predicted emergence aligns well with a previous study, which showed that the mean age of the Agaricomycetes was ~290 million years ago in both BEAST and PhyloBayes analyses (95% highest posterior density interval = 222–372 million years) (52). Interestingly, the evolutionary timeline of vascular plants indicates that the diversification of angiosperms also commenced approximately 250–140 million years (53), suggesting that the evolution of angiosperms overlapped with the evolution of Agaricomycetes. Therefore, the phylogenetic tree of laccase genes among these fungi in the Class Agaricomycetes was further analyzed. The clustering of sequences in the phylogenetic tree did not strictly adhere to the taxonomic relationships of the species from which they were derived (Fig. 7). Specifically, the laccases present in the upper part of the tree, as exemplified by the *D. squalens* fungus, were typically associated with wood-decaying fungi. In contrast, in the lower part of the tree, some sequences from *D. squalens* were observed within different subclusters. This phenomenon could be explained by the variability in oxidase requirements within the same species, which may lead to the differentiation of homologous copies of laccase (22). Recent works have resurrected some protein sequences from extinct organisms to investigate enzyme evolution through ancestral sequence reconstruction—a methodology involving the functional expression of inferred ancestral proteins in modern microbial systems (30, 54). Notably, Catania et al. (55) employed this approach to elucidate the evolutionary progression of substrate preference shifts in the salicylic acid methyltransferase lineage of flowering plants. Herein, LacAnc160, LacAnc169, and LacAnc178 were constructed at different ancestral nodes, expressed, and purified to homogeneity (Fig. S15). The oxidation efficiency of softwood lignin oxidized by ancestral laccases LacAnc160 and LacAnc169 was found to be higher than that of LacAnc178. However, when hardwood lignin was utilized as the substrate, the oxidation efficiency exhibited an opposite trend. This observation aligned with the oxidation pattern observed in *T. hirsuta* AH28-2. Moreover, the enzymatic modifications of lignin in both softwood and hardwood treated with ancestral laccases were observed through Py-GC/MS and FTIR analyses (Fig. 8). These analyses revealed a switch from better oxidation of softwood lignin by the oldest ancestral laccase LacAnc160 to better oxidation of hardwood lignin by the later ancestral laccase LacAnc178. With regard to hardwood lignin, in particular, these findings indicate that the capacity of laccase to oxidize the S-type units increased as the evolutionary process advanced.

### Conclusion

**Fig 9:**
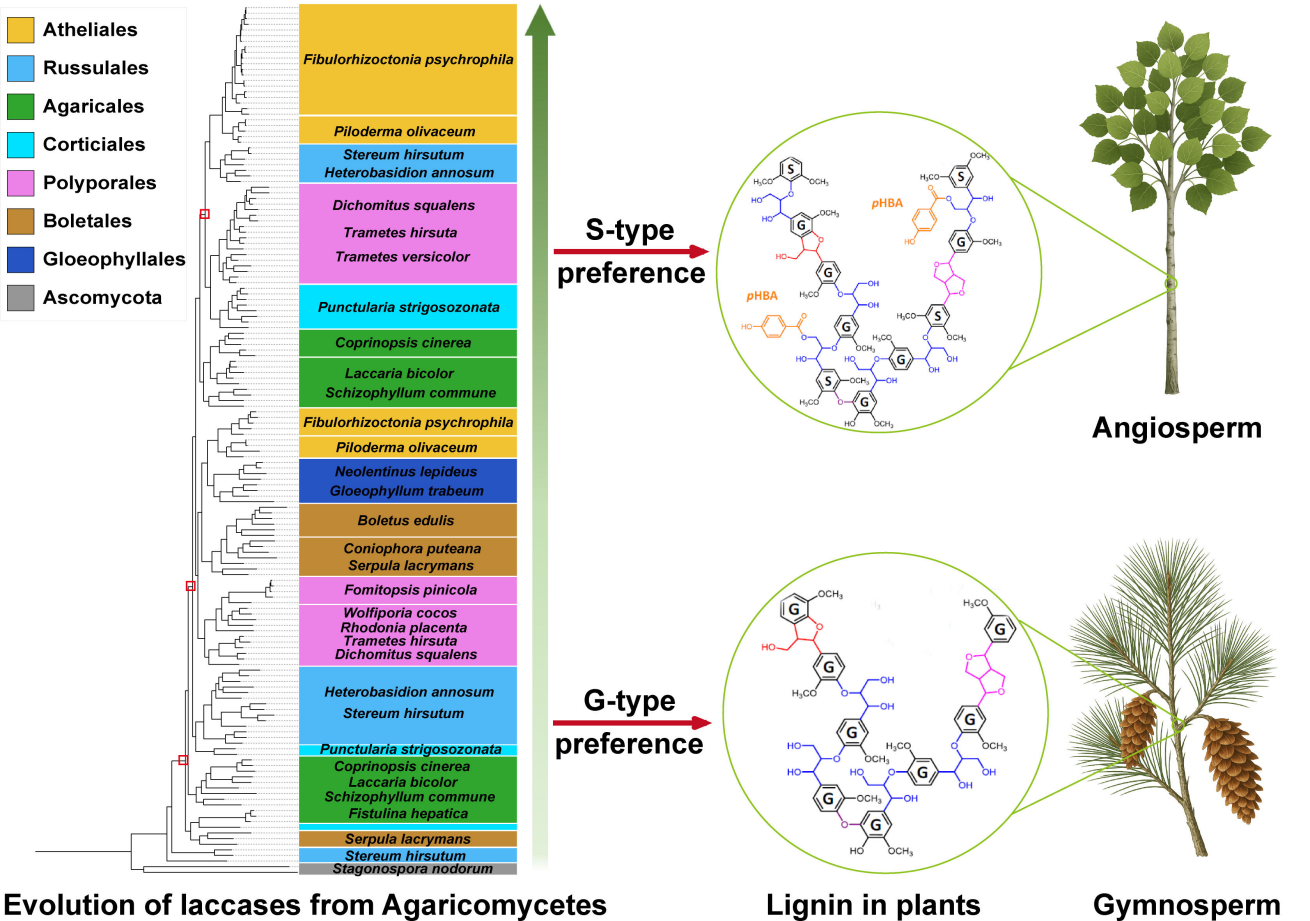
The adaptive evolution of Agaricomycetes fungi laccases parallels lignin diversification of wood from gymnosperm to angiosperm.

The six laccase isozymes identified in *T. hirsuta* AH28-2 are classified into three distinct evolutionary branches. Notably, LacF occupies an independent early evolutionary branch and primarily contributes to oxidizing G-type units found in gymnosperm lignin. In contrast, LacA and LacB represent later-emerging isozymes that primarily contribute to oxidizing S-type units present in angiosperm lignin. Ancestral laccases at various evolutionary nodes also make distinct contributions to lignin oxidation, suggesting that the diversification of fungal laccases is closely linked to the emergence of S-type units in angiosperm lignin. The study demonstrates that the adaptive evolution of Agaricomycetes laccases follows the diversification of wood lignin in plants. Furthermore, laccases at different evolutionary stages exhibit distinct oxidative performance for different types of lignin (Fig. 9).

## MATERIALS AND METHODS

### Strains, chemicals, and culture media

*T. hirsuta* strain AH28-2 (China Center for Type Culture Collection No. AF2015027) was maintained on compound potato dextrose agar (PDA, containing 200 g/L boiled potato, 20 g/L glucose, 3 g/L KH_2_PO_4_, 1.5 g/L MgSO_4_·7H_2_O, 0.05 g/L vitamin B1, and 15 g/L agar) plates at 4°C. *Escherichia coli* JM109, *Pichia pastoris* GS115, and the plasmid pPIC9K were purchased from Invitrogen (Carlsbad, CA, USA). Chemicals, including ABTS, guaiacol, 5-nitroguaiacol, guaiacol glyceryl ether, vanillic acid, isovanillin, *p*-hydroxybenzoic acid, 4-hydroxybenzaldehyde, 4-hydroxyphenylacetic acid, 2-(4-hydroxyphenyl) ethanol, 4-hydroxyacetophenone, *p*-methylphenol, syringic acid, syringaldehyde, and acetosyringone were obtained from Sigma-Aldrich (St. Louis, MO, USA). All other chemicals and reagents were of analytical grade. Three wood species, including larch (*Larix gmelinii*), poplar (*Populus tomentosa*), and bamboo (*Phyllostachys heterocycla*), were purchased from the local market. Taxonomically, larch belongs to gymnosperms, poplar to dicotyledons, and bamboo to monocotyledons. All woods, free of bark, were air-dried and ground to pass through a 20-mesh screen and then oven-dried at 50°C for 3 days. The XH medium (containing 15.0 g/L cellobiose, 1.0 g/L peptone, 1.5 g/L DL-asparagine, 0.1 g/L Na_2_HPO_4_, 1.0 g/L KH_2_PO_4_, 0.5 g/L MgSO_4_·7H_2_O, 0.01 g/L CaCl_2_, 1.0 mg/L FeSO_4_·7H_2_O, 27.5 mg/L adenine, 0.05 mg/L vitamin B1, and 2 mg/L CuSO_4_·7H_2_O) was used for the liquid culture of *T. hirsuta* AH28-2, as previously described (56).

### Fungal submerged cultures

The liquid cultures of *T. hirsuta* AH28-2 were performed according to the methods described previously (57). *T. hirsuta* AH28-2 cells stored in 20% glycerol (vol/vol) at −80°C were activated and grown on PDA plates at 28°C for 9 days. Six plugs (diameter 5 mm) were inoculated into XH medium. After incubation at 28°C and 120 rpm for 4 days, the liquid culture was homogenized using a sterile blender at 3,000 rpm for 10 s, and 5 mL of the culture was inoculated into a new 50 mL XH medium. After incubation at 28°C and 120 rpm for 3 days, 13 lignin model compounds [5-nitroguaiacol, guaiacol, guaiacol glyceryl ether, vanillic acid, 4-hydroxybenzaldehyde, 2-(4-hydroxyphenyl) ethanol, *p*-methylphenol, 4-hydroxyphenylacetic acid, 4-hydroxyacetophenone, *p*-hydroxybenzoic acid, syringic acid, syringaldehyde, and acetosyringone] were added to the culture medium at a final concentration of 1 mM as required, respectively. The time point of aromatic compound addition was designated as time zero (0 h). Samples were collected thereafter at 12-h intervals. Cultures without agent addition were used as controls. All experiments were performed three times.

### Laccase activity assay and native PAGE analysis

Laccase activity was tested using guaiacol as the substrate, as previously described (32). Native PAGE was performed using 12% polyacrylamide gels according to the standard protocol. The gels were incubated in citrate-Na_2_HPO_4_ (50 mM, pH 4.5) containing different laccase substrates, including ABTS, guaiacol, 5-nitroguaiacol, guaiacol glyceryl ether, vanillic acid, isovanillin, *p*-hydroxybenzoic acid, 4-hydroxybenzaldehyde, 4-hydroxyphenylacetic acid, 2-(4-hydroxyphenyl) ethanol, 4-hydroxyacetophenone, *p*-methylphenol, syringic acid, syringaldehyde, and acetosyringone. Subsequently, they were photographed using a digital camera. The concentrations of laccase isozymes were calculated using ImageJ software (version 1.8.0).

### qRT-PCR analysis of genes at the transcriptional level

The mycelia of *T. hirsuta* AH28-2 from different cultures, treated or untreated with lignin model compounds for different time points, were collected for total RNA extraction. Then, 1 μg of total RNA was used as the template for cDNA synthesis following the PrimeScript RT kit (TaKaRa) instructions (58). The transcriptional levels of laccase isozyme genes were analyzed by a LightCycler 96 real-time PCR system (Roche, Basel, Switzerland). Real-time PCR was performed using a SYBR green kit (TaKaRa). The gene *gapdh* was used as a reference gene to normalize the qRT-PCR data (59). The 2*^−△△CT^* method was used to calculate the relative expression levels of each gene (60). According to genomic sequences, seven primers of laccase isozyme genes for qRT-PCR were designed (Table 2).

**TABLE 2:** Primers used in this study

Name	Primer sequence	Purpose
qRT*-lacA*-F	TCCTTCGTGTTGAATGCCGA	qRT-PCR of *lacA*
qRT*-lacA*-R	GTTGATACCGCCCGCAAATC	qRT-PCR of *lacA*
qRT*-lacB*-F	CGTTCTGGTACCACAGCCAT	qRT-PCR of *lacB*
qRT*-lacB*-R	TCGTACATGGACGCATACGG	qRT-PCR of *lacB*
qRT*-lacC*-F	AAGTCGACCAGCATCCACTG	qRT-PCR of *lacC*
qRT*-lacC*-R	TCGTAAAGGAACGAGTGCCC	qRT-PCR of *lacC*
qRT*-lacD*-F	TTCGCTGGTGGGATCAACTC	qRT-PCR of *lacD*
qRT*-lacD*-R	CTTTCCGGGCACAGTCATCT	qRT-PCR of *lacD*
qRT*-lacE*-F	CGTTCTGGTACCACAGCCAT	qRT-PCR of *lacE*
qRT*-lacE*-R	CAACGTCGTAGAGATCCGCA	qRT-PCR of *lacE*
qRT*-lacF*-F	CATTACCTTGGCCGACTGGT	qRT-PCR of *lacF*
qRT*-lacF*-R	TCGACTTCAATGACCGCCAA	qRT-PCR of *lacF*
qRT*-gapdh*-F	GCCGCTTCAAGGGCAAAGTC	qRT-PCR of *gapdh*
qRT*-gapdh*-R	TGTAGTCGGCACCAACGGA	qRT-PCR of *gapdh*
*lacA*-F	AAATATTACGTAATTGGGCCCACCGCTGACCT	Cloning of *lacA* fragment
*lacA*-R	AAATATCCTAGGCTACTGGTCGTTGACGTCGAG	Cloning of *lacA* fragment
*lacB*-F	GTCAGTTACGTAATTGGCCCAGTCACCGACCT	Cloning of *lacB* fragment
*lacB*-R	GTCAGTCCTAGGCTAGTGGTCAGACGGGTCGA	Cloning of *lacB* fragment
*lacC*-F	AAATATTACGTAATCGGCCCCGTGACCGACCT	Cloning of *lacC* fragment
*lacC*-R	AAATATCCTAGGTCACAGGTCGTTCTCGTCGAGC	Cloning of *lacC* fragment
*lacD*-F	ATTATTTACGTATAGCCTGGCTTCCGCTTGGA	Cloning of *lacD* fragment
*lacD*-R	GAATTCTCACAAGTCGCCCTCGCCCA	Cloning of *lacD* fragment
*lacE*-F	ATTATTTACGTAGCTATCGGTCCCGTAGCCGA	Cloning of *lacE* fragment
*lacE*-R	ACTATCCCTAGGTCAGAGGTCAGACGAGTCCAAG	Cloning of *lacE* fragment
*lacF*-F	GTCAGTTACGTAGCGATCGGCCCAGTCACTGAGCT	Cloning of *lacF* fragment
*lacF*-R	AAATATGCGGCCGCTTAAGGGTTGATGTGCATCGACTG	Cloning of *lacF* fragment
An-*lacA*-F	ATCTACACACAACAAGCTTATCGCCTTCGCTCGTCAGGCCGTGGTTGTCA	DNA template of *lacA* silencing
An-*lacA*-R	GCCCTCTGGTCAACTATAATATTATGGTACCAATCCGCGAGTGTGATCAC	DNA template of *lacA* silencing
An-*lacB*-F	ATCTACACACAACAAGCTTATCGCCATGACCATCATTGAGGTGGACGGTG	DNA template of *lacB* silencing
An-*lacB*-R	GCCCTCTGGTCAACTATAATATTATCGGGCACGGTGGGCGGGATGAATGT	DNA template of *lacB* silencing
An-*lacF*-F	ATCTACACACAACAAGCTTATCGCCGATGATGAGAGCACCGTCATTACCT	DNA template of *lacF* silencing
An-*lacF*-R	GCCCTCTGGTCAACTATAATATTATCGTTAGAGAAGCCGGTGGTGTTAGC	DNA template of *lacF* silencing

### Sequence and phylogenetic analysis

The amino acid sequences of laccase isozymes derived from *Trametes* sp. were retrieved from the NCBI database, with those of *T. hirsuta* AH28-2 provided in the supplemental material. Multiple Expectation Maximization for Motif Elicitation (http://meme-suite.org/) and Simple Modular Architecture Research Tool (http://smart.embl.de) were used to identify and annotate laccase domains (61). Clustal W program was used for sequence alignment (62). MEGA 11 (http://www.megasoftware.net/) was used for phylogenetic analysis.

### Substrate spectrum screening and redox-potential determination of the recombinant laccases

The full-length cDNA of each laccase isozyme was amplified using specific primers (Table 2). Laccase expression in *P. pastoris* and purification were conducted according to the protocols reported previously (63, 64). The substrate range of the recombinant laccases was determined in 96-well plates, as described in reference 65. Briefly, reactions were performed in 96-well plates in 50 mM citrate/phosphate buffer in a total volume of 200 μL at pH 6.0 and 30°C, with shaking at 200 rpm. The reaction was initiated by adding 2 μL of properly diluted laccase. A UV-Vis spectrum was recorded in the range of 250–750 nm prior to enzyme addition and after a 24-h reaction time. The redox potential of laccases was measured using cyclic voltammetry at pH 5.0 according to the method described previously (64).

### Laccase treatment of three types of native woods

Three natural lignocellulosic materials—larch, poplar, and bamboo—were used as substrates for this study. Their chemical compositions were as follows: larch wood contained 46.7% cellulose, 18.3% hemicellulose, and 34.6% lignin; poplar wood comprised 45.1% cellulose, 19.2% hemicellulose, and 29.5% lignin; and bamboo wood consisted of 42.3% cellulose, 24.8% hemicellulose, and 26.8% lignin. The recombinant laccases were used for lignin degradation. Assays were carried out in 250 mL Erlenmeyer flasks containing 2.5 g ground wood (larch, poplar, or bamboo) in a total volume of 50 mL and incubated at 50°C in a water bath shaker (200 rpm) for 24 h. The enzyme load of 5 U/g dry substrate was added. The experiment was repeated three times. Laccase activity was determined at 50°C and pH 5.0, 4.5, or 4.5 for rLacA, rLacB, or rLacF, respectively, using 5 mM guaiacol as the substrate to ensure that the same amount of laccase activity was added (26). Control assays were performed under the same conditions without laccase addition. The residual lignin of samples was quantified according to the method described by Templeton et al. (66).

### Analysis of lignin oxidation products by GC-MS

Extractives-free lignocellulosic materials were used as substrates in this section. The substrates were first obtained by sequentially extracting the ground wood powder (larch, poplar, and bamboo) with ethanol (>99.5%), followed by distilled water, with each extraction step lasting 1 h. The resulting extractives-free wood powder was dried at 45°C for 24 h under vacuum and subsequently stored in a desiccator prior to use. After incubation at 50°C for 24 h, the reaction mixture was extracted with ethyl acetate, concentrated, redissolved in methanol, and analyzed by a GC-MS system (THERMO TRACE 1310 equipped with a TSQ 8000 Evo mass spectrometer). The oven temperature was programmed as described by Senthilvelan et al. (67). The NIST 05a.L mass spectrometry library was used for compound identification. Detailed protocols are described in the supplemental material.

### Fungal biodegradation in native wood media

Six mycelial plugs (5 mm in diameter) of each strain (*T. hirsuta* AH28-2 WT and *lacA*-silenced, *lacB*-silenced, and *lacF*-silenced transformants) from the actively grown plates were inoculated into liquid XH medium and continuously shaken at 120 rpm in the dark for 4 days. After homogenization, the mycelial suspension was used to inoculate a 150 mL medium containing 2 g of native wood sawdust (Larch, Poplar, or Moso-bamboo) (9). The agitated (120 rpm) cultivations were performed as three biological replicate cultures in 250 mL baffled Erlenmeyer flasks and incubated at 28°C for 20 days. For extracellular enzyme activity analysis, fungal culture liquid was collected via suction filtration. The wood residue was freeze-dried and ground into a powder for later use. Laccase activity was determined by 2, 2′-azino-bis (3-ethylbenzothiazoline-6-sulfonic acid) (68). MnP activity was determined by the 2, 6-dimethylphenol method (69). The veratryl alcohol was used to assess Lip activity (70). The mycelial biomass of fungi was determined by measuring the content of ergosterol. Ergosterol was extracted and quantified using the method described by Wu et al. (71). The lignin content was determined using the method mentioned above.

### Ancestral laccase reconstruction

The genomes of 43 diverse fungi, already available at the DOE JGI MycoCosm database (https://mycocosm.jgi.doe.gov/mycocosm/home), were used in the present study. These species represent four different ecological habits (Table S20). *T. hirsuta* LacB (PDB: 3KW7) was used to blast against the genomes of 43 species. Amino acid sequences that did not include the four laccase signature sequence regions identified by Kumar and coworkers (72) were deleted. These regions correspond to those that differentiate them from the broader class of multicopper oxidases. The alignment was manually adjusted with MUSCLE as implemented in MEGA 11 (73). The phylogenetic tree was then constructed via maximum likelihood phylogenetic reconstruction with RAxML (version 8.2.12) (74), with the PROTGAMMABLOSUM62 model and 1,000 bootstrap replicates. PAML X was used for ancestral sequence reconstruction (75). The reconstructed ancestral laccase genes were synthesized by General Biol (Chuzhou, Anhui, China) and expressed in *P. pastoris* as described in the supplemental material. The expression, purification, and biological treatment of ancestral laccases (sequences in the supplemental material) were performed according to the methods mentioned above.

### Structure characterization of lignin from pretreated biomass

#### Py-GC/MS analysis

Pyrolysis of control and treated samples was performed with a CDS analytical Pyro probe 5200 connected to a GC-MS (Agilent Technologies Inc., Palo Alto, CA, USA). The pyrolysis was performed at 500°C at a 20°C ms^−1^ heating rate and held for 30 s. The GC temperature was initially set at 50°C and maintained for 1 min, then increased to 280°C at a rate of 5°C/min and held for 5 min. Helium was used as a carrier gas (1 mL/min). The compounds were identified by searching in the National Institute of Standards and Technology mass spectra library. They were classified and summed according to their structural features. Relative abundances of lignin-derived pyrolysis products were calculated based on areas, as previously described by del Río et al. (76). All samples were prepared and analyzed in triplicate.

#### FTIR analysis

Both treated and untreated wood samples were analyzed using FTIR (Bruker, Vertex 80) to investigate the modifications in the chemical moieties of lignin during degradation. A representative sample, with weight loss equivalent to the group average, was selected for FTIR analysis from the enzyme-treated specimens. A vacuum was used to dry the samples into fine powder. Two milligrams of dried wood samples was mixed with dry KBr (200 mg), and then, the homogenized samples were compressed to make thin pellets under continuous pressure of 40 MPa. The spectral range was taken from 400 to 4,000 cm^−1^ at a resolution of 4 cm^−1^. The attribution of each absorption peak was based on literature and is detailed in Table S7 (77–80). The relative transmittance of each peak was calculated with the transmittance of the aromatic skeletal vibration at 1,510 cm^−1^ as the standard (49, 81).

#### Scanning electron microscopy analysis

Both treated and untreated wood samples were examined through scanning electron microscope (SEM) (Hitachi S-4800) as described by Nazar et al. (25). A representative sample, with weight loss equivalent to the group average, was selected for SEM analysis from the enzyme-treated specimens. A vacuum was used to dry the samples into fine powder and then mounted on carbon tape. In addition, they were sputter-coated with gold for viewing and imaging. SEM analysis was performed at a magnification of ×10,000, and micrographs from multiple regions were acquired to thoroughly evaluate the effect of laccases or fungi on the morphological and physical characteristics of wood samples. Representative images are shown.

### Statistical analyses

Statistical analyses were performed using SPSS 27.0 (SPSS Inc., Chicago, IL, USA). The mean values among different treatment groups were compared by one-way analysis of variance with Duncan’s *post hoc* test. The significance level was set at *P* < 0.05.

## Data Availability

All data supporting the findings of this study are presented in the paper or the supplemental material. The species phylogenetic tree is available at https://itol.embl.de/tree/21045209228129681668436078. The laccase phylogenetic tree is available at https://itol.embl.de/tree/6016815519402261669078862.

## SUPPLEMENTAL MATERIAL

Supplemental material aem.01971-25-s0001.docx Supplemental methods, Fig. S1 to S16, and Tables S1 to S20.

ASM does not own the copyrights to Supplemental Material that may be linked to, or accessed through, an article. The authors have granted ASM a non-exclusive, world-wide license to publish the Supplemental Material files. Please contact the corresponding author directly for reuse.
