# Supplementary material for: Adaptive evolution of Agaricomycetes laccases follows wood lignin diversification in plants
Source: Appl Environ Microbiol. 2026 Jan 13;92(2):e01971-25. doi: 10.1128/aem.01971-25 (PMC12915358; doi:10.1128/aem.01971-25)
Supplement: Supplemental material — Supplemental methods, Fig. S1 to S16, and Tables S1 to S20. [file aem.01971-25-s0001.docx]

**Supplemental Material**

**Adaptive evolution of Agaricomycetes laccases** **follows wood lignin diversification in plants**

Shenglong Liu^1,2,3^, Qinbiao Yu^1,2,3^, Tian Yin^1,2,3^, Xinlei Zhang^1,2,3^, Rongrong Zhou^1,2,3^, Chenkai Wang^1,2,3^, Yazhong Xiao^1,2,3*^, Juanjuan Liu^1,2,3*^, Zemin Fang^1,2,3*^

1 School of Life Sciences and Medical Engineering, Anhui University, Hefei, Anhui 230601, China;

2 Anhui Key Laboratory of Biocatalysis and Modern Biomanufacturing, Hefei, Anhui 230601, China;

3 Anhui Provincial Engineering Technology Research Center of Microorganisms and Biocatalysis, Hefei, Anhui 230601, China;

*Corresponding authors: Yazhong Xiao, Juanjuan Liu, and Zemin Fang

Phone/Fax: +86-551-63861063

Email: yzxiao@ahu.edu.cn (to XY); liu_juan825@ahu.edu.cn (to LJ); zemin_fang@ahu.edu.cn (to FZ)

**Supporting materials and methods**

**Gas chromatography-mass spectrometry (GC-MS) analysis of degradation products**

After the degradation reaction, the mixture was extracted three times with an equal volume of ethyl acetate. The combined organic phases were concentrated using a rotary evaporator and dried under a gentle stream of nitrogen gas. The residue was redissolved in 1.0 mL of methanol and filtered through a 0.22 μm membrane filter before analysis. GC-MS analysis was performed on a THERMO TRACE 1310 gas chromatograph coupled to a TSQ 8000 Evo mass spectrometer. Separation was achieved using an HP-5 silica capillary column (30 m × 0.25 mm × 0.25 μm, Thermo Scientific). Helium was used as the carrier gas at a constant flow rate of 1.0 mL/min. The GC oven temperature program was as follows: 100 °C held for 2 min, increased to 250 °C at a rate of 15 °C/min, then increased to 300 °C at a rate of 30 °C/min.

**Construction of** ***lacA*-, *lacB*-, and *lacF*-silenced *T. hirsuta* AH28-2 transformants**

The *laccases* (*lacA*, *lacB*, and *lacF*) silencing plasmids were constructed based on the recombinant plasmid pYSK7, as previously described (1, 2). Briefly, a 400-bp partial cDNA (bp +844 to +1243 bp) of *lacA*, a 400-bp partial cDNA (bp +773 to +1172 bp) of *lacB* and a 402-bp partial cDNA (bp +183 to +584 bp) of *lacF* were amplified using the primer pairs of An-*lacA*-F and An-*lacA*-R, An-*lacB*-F and An-*lacB*-R, and An-*lacF*-F and An-*lacF*-R, respectively (Table S1). Then, they were inserted into pYSK7 to generate three pYSK7-*An* plasmids through homologous recombination in the yeast *S. cerevisiae* Y1H. *T. hirsuta* AH28-2 oidia were collected, treated with an enzyme solution to form protoplasts, and co-transformed with each pYSK7-*An* plasmid and pCRII-*hph* vector mediated by the PEG/CaCl_2_ method (3, 4). Positive transformants were screened and further validated via genomic PCR amplification of the antisense fragments of laccases (*lacA*, *lacB*, and *lacF*) using primers PF and PR listed in Table S1 (3). Three *lacA-*silenced positive transformants, three *lacB-*silenced positive transformants, and three *lacF-*silenced positive transformants were randomly selected for qRT-PCR analysis to quantify the transcriptional levels of *lacA*, *lacB*, and *lacF*, respectively. Meanwhile, their laccase activity was analyzed.

**Phylogenetic analysis**

The fungi used in the present study were shown in Table S20. These species included 27 Agaricomycetes and 16 other fungi. Single-copy homologous genes were selected for multiple sequence alignment, which was conducted using MAFFT software (5), followed by a quality control assessment of the alignment. The alignment was further refined with Gblocks to eliminate poorly aligned regions (6). A maximum likelihood (ML) phylogenetic tree was reconstructed (7), and branch support was evaluated with 1000 bootstrap replicates.

For divergence time estimation, fossil age records from specific taxonomic groups were used to calibrate the minimum ages of different clades in the Dikarya. *Paleopyrenomycites devonicus* was a 400 my old ascomycete fossil from the early Devonian that was associated with the extinct vascular plant *Asteroxylon mackiei* (8). The split between the subphyla Saccharomycotina and Pezizomycotina in the Ascomycota was calibrated with *P*. *devonicus* (9). The divergence times of the remaining nodes were inferred based on branch lengths. Bayesian divergence time estimation was performed using the mcmctree program within the PAML package, supplemented by additional calibration utilizing published divergence times from the TimeTree database (http://www.timetree.org/).

**Ancestral laccase gene expression in *Pichia pastoris* and purification**

After laccase gene synthesis, the recombinant plasmids pPIC9K-*LacAnc160*, pPIC9K- *LacAnc169*, and pPIC9K- *LacAnc178* and the control vector pPIC9K were linearized by using *Sac* I and transformed into *P. pastoris* GS115 by electroporation according to the instruction of the Multi-Copy *Pichia* Expression Kit (Invitrogen). The active enzymes were purified according to Xu et al.(10).


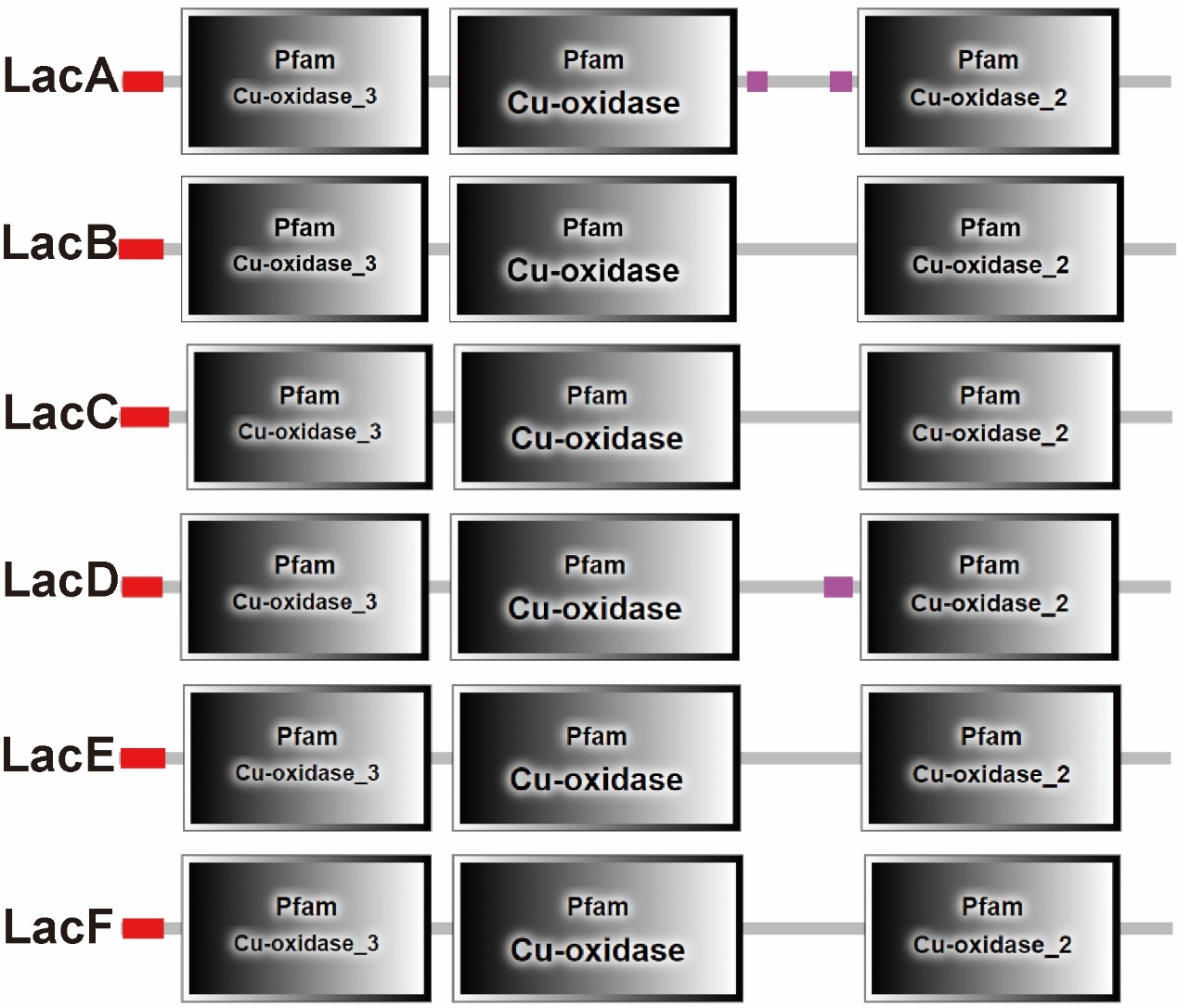


**Figure S1 Analysis of the amino acid sequence domain of laccase isoenzymes from *T. hirsuta* AH28-2.**


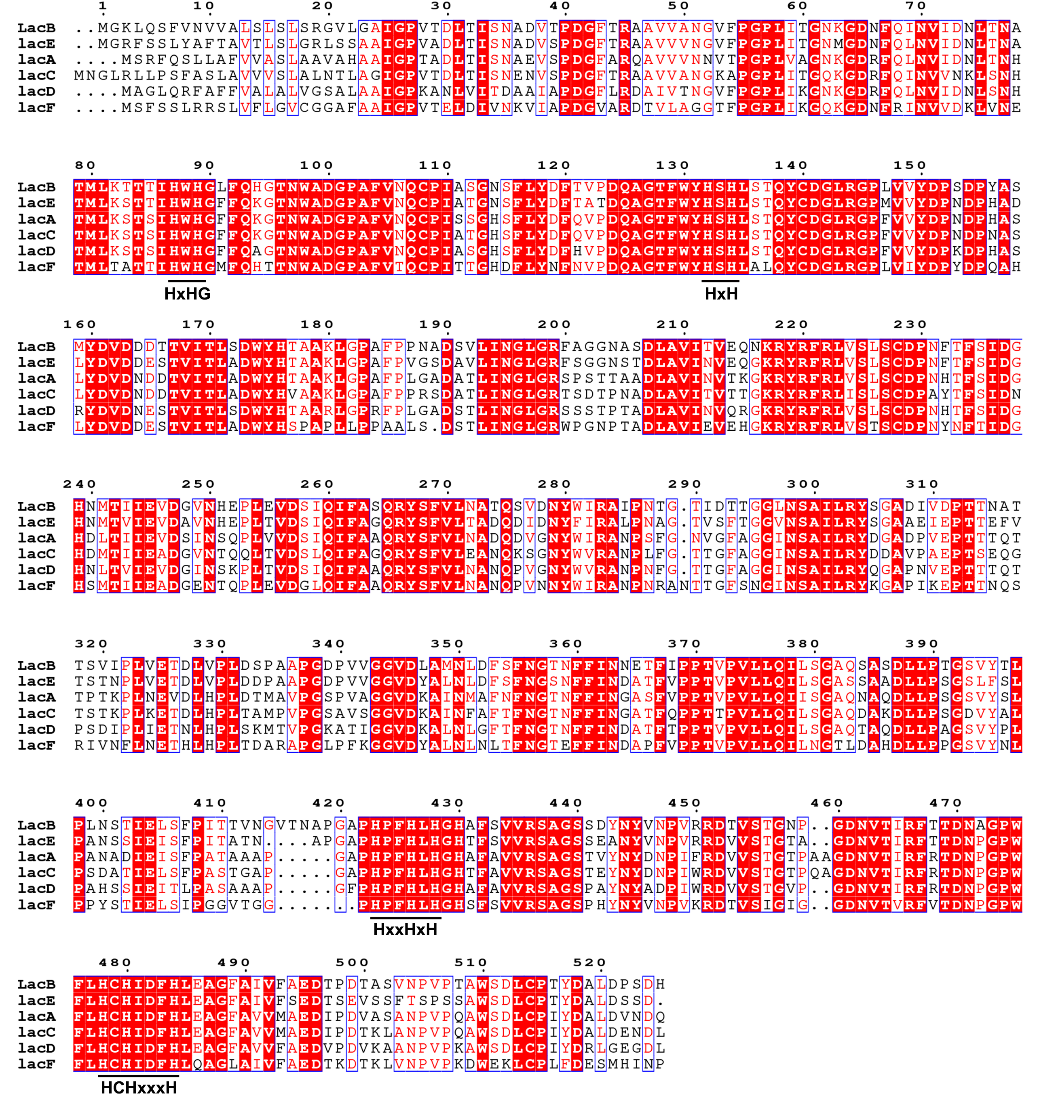


**Figure S2 Amino acid sequence homology analysis of laccase from *T. hirsuta* AH28-2.**


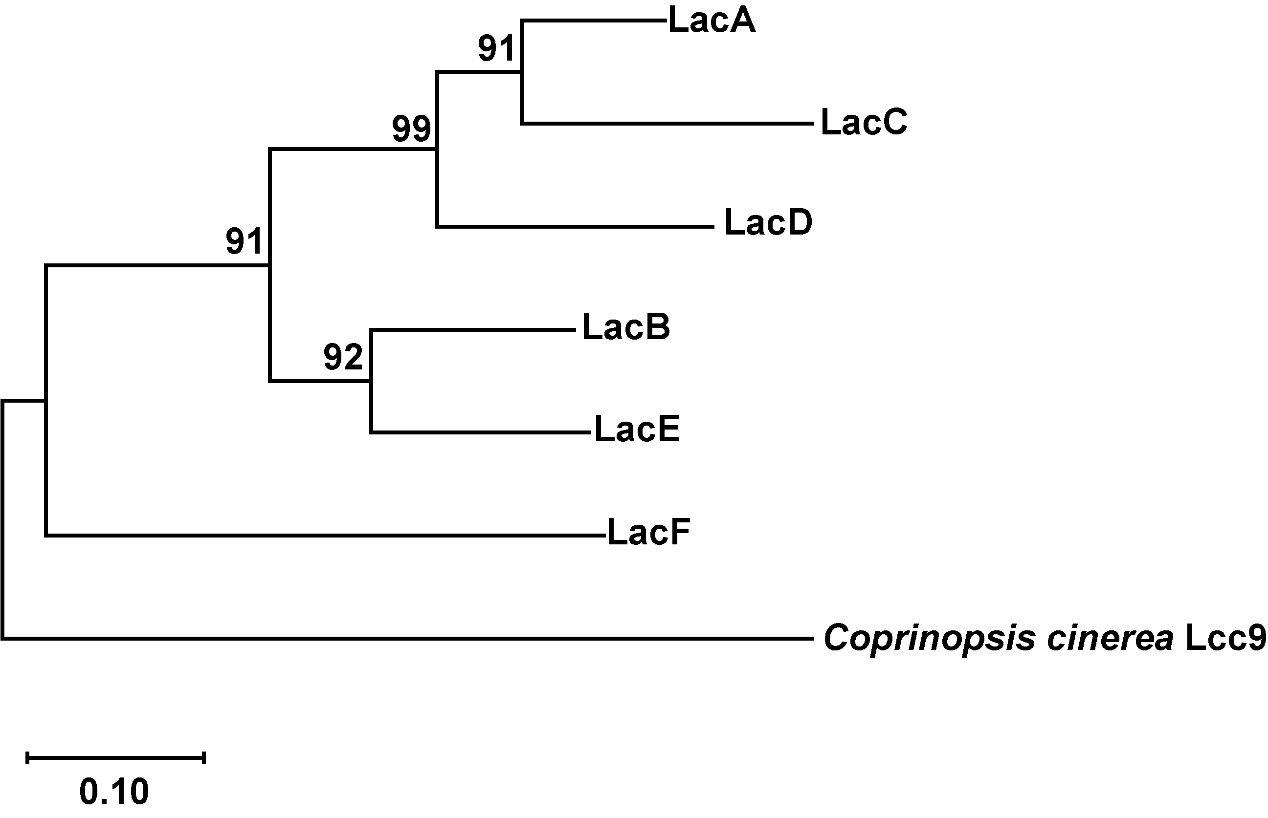


**Figure S3 Phylogenetic analysis of the laccase gene multigene family of *T. hirsuta* AH28-2.**


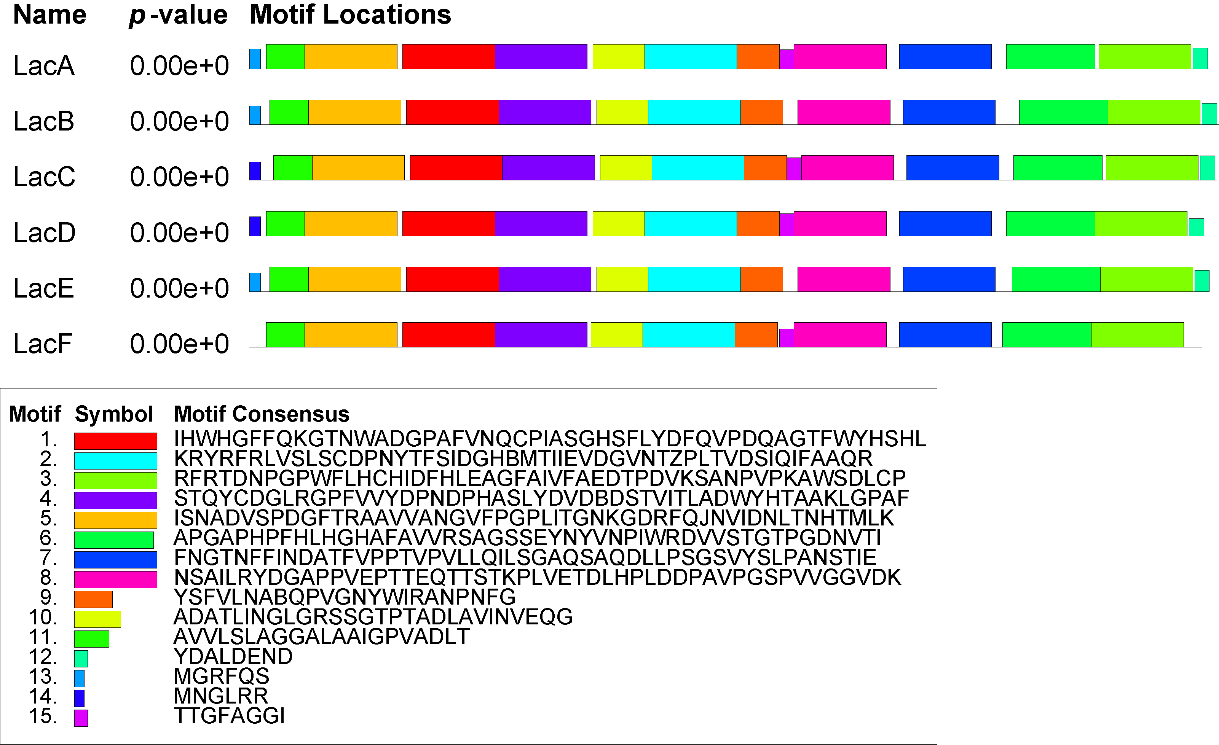


**Figure S4 Conserved motifs of the laccase gene multigene family of *T. hirsuta* AH28-2.**


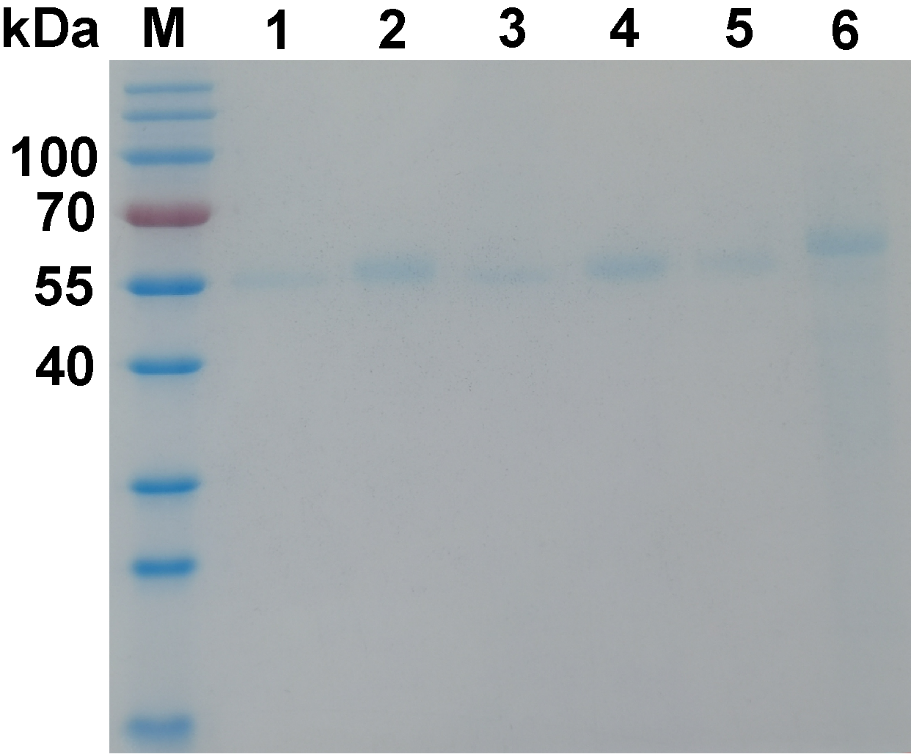


**Figure S5 SDS-PAGE of purified laccases.** Lanes: M: protein marker; 1: rLacA; 2: rLacB; 3: rLacC; 4: rLacD; 5: rLacE; 6: rLacF.


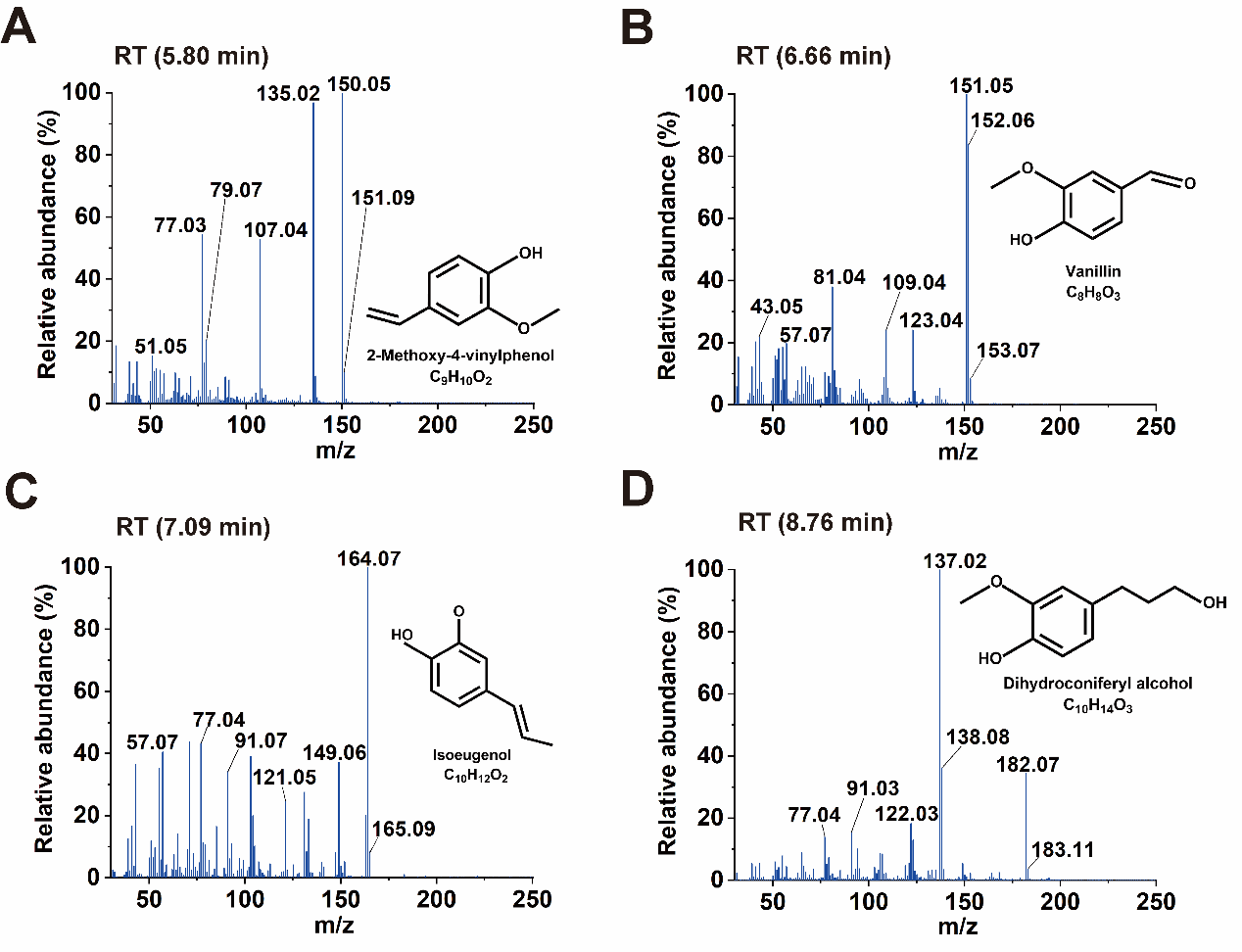


**Figure S6 Mass spectrum of larch lignin degradation products by laccase rLacA.** (A) 2-Methoxy-4-vinylphenol; (B) Vanillin; (C) Isoeugenol; (D) Dihydroconiferyl alcohol.


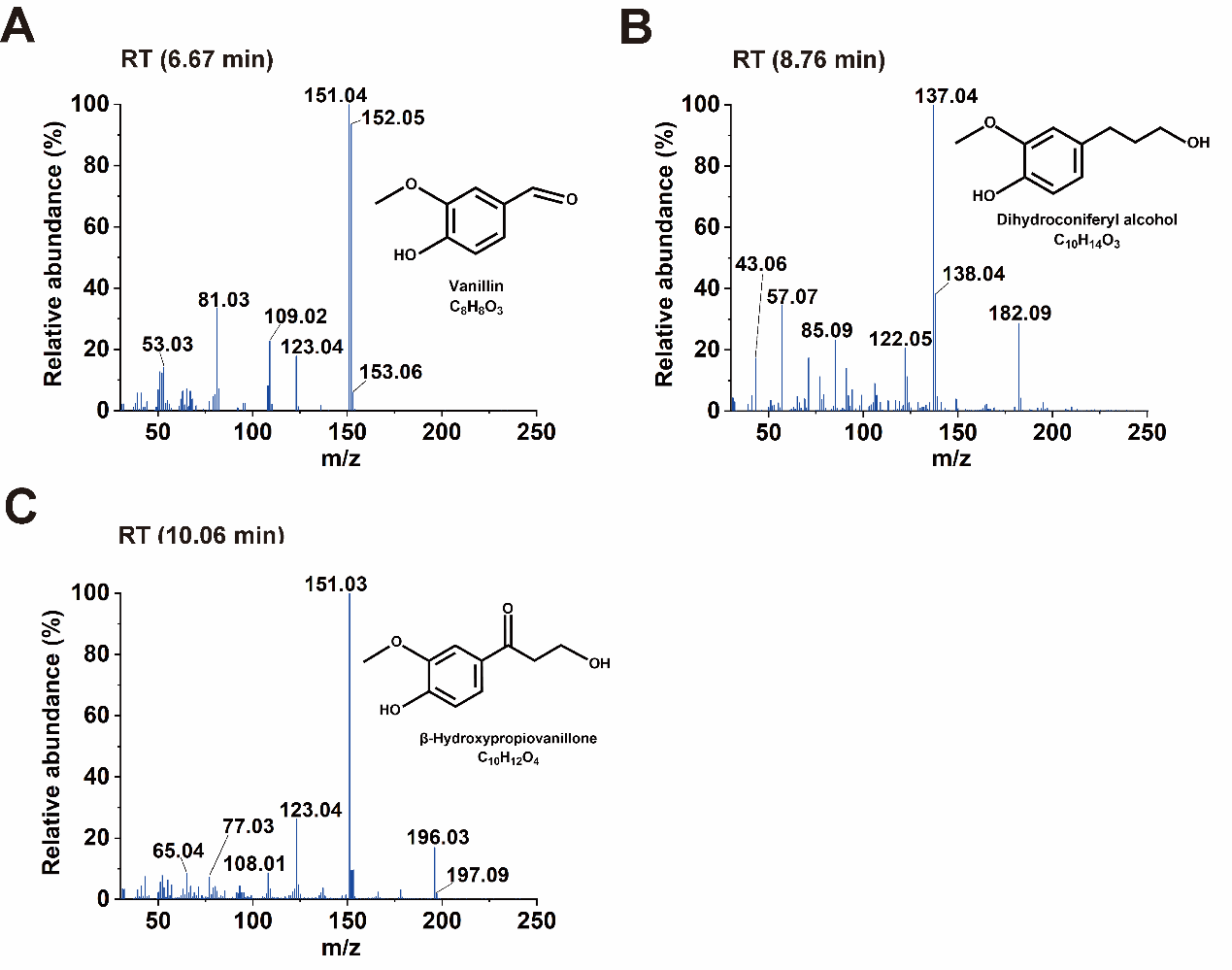


**Figure S7 Mass spectrum of larch lignin degradation products by laccase rLacB.** (A) Vanillin; (B) Dihydroconiferyl alcohol; (C) *β*-Hydroxypropiovanillone.


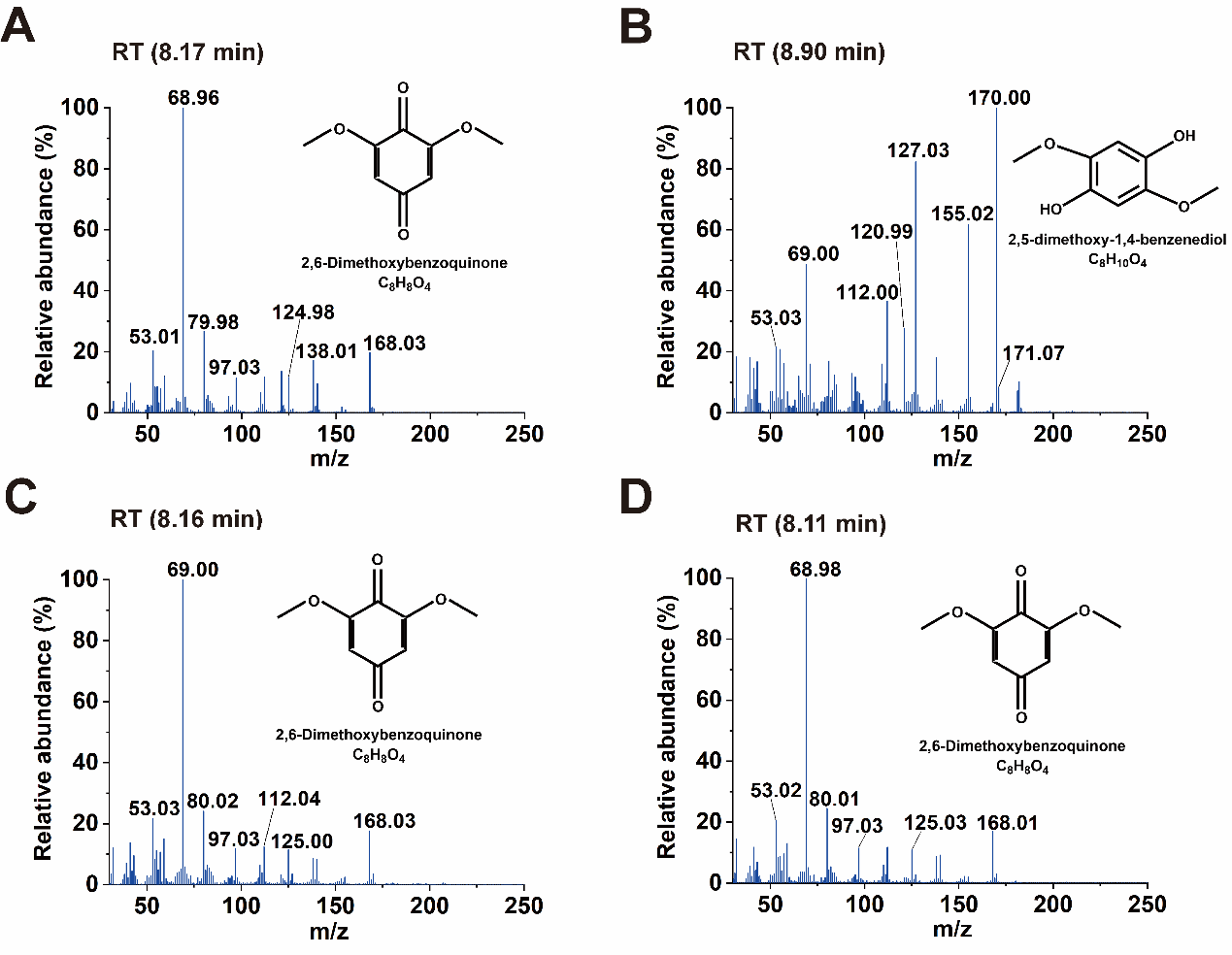


**Figure S8 Mass spectrum of poplar lignin degradation products by laccases.** (A) 2,6-Dimethoxybenzoquinone and (B) 2,5-Dimethoxy-1,4-benzenediol were degradation products of rLacA; (C) 2,6-Dimethoxybenzoquinone was the degradation product of rLacB; (D) 2,6-Dimethoxybenzoquinone was degradation product of rLacF.


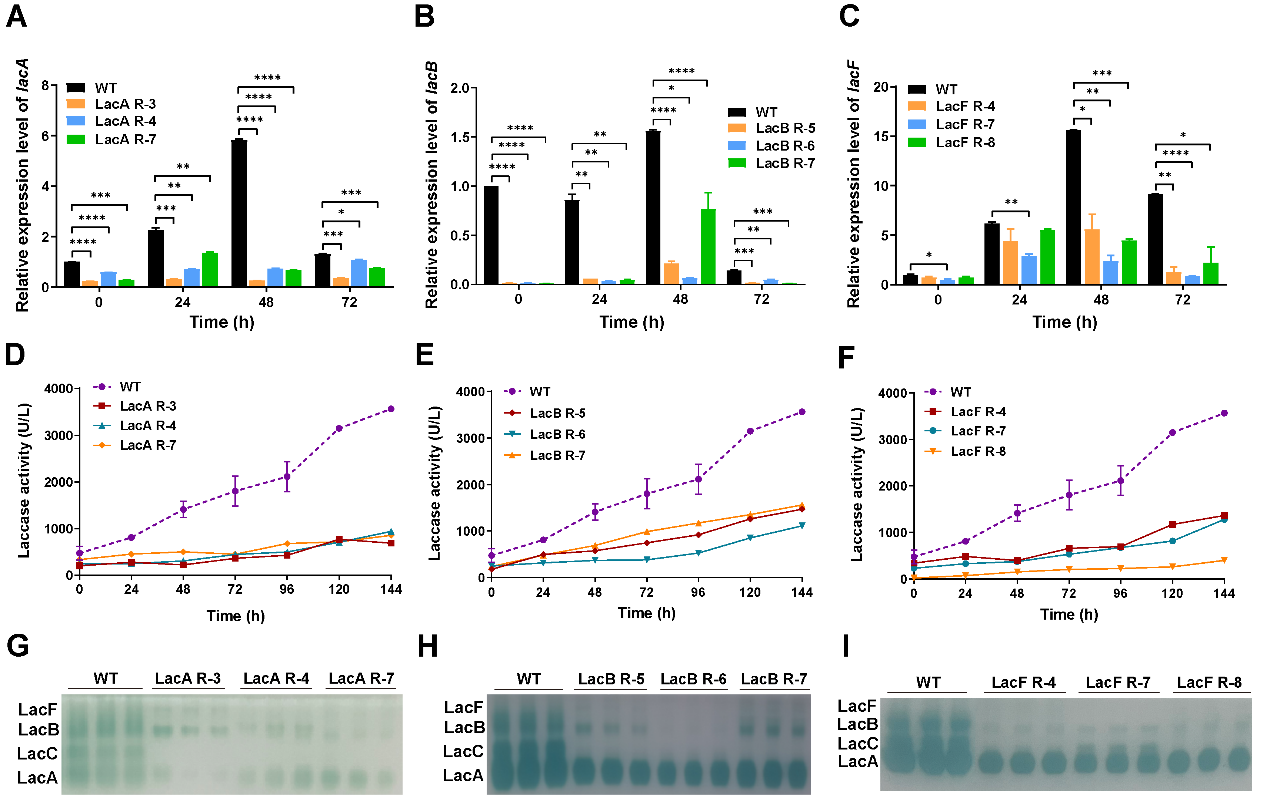


**Figure S9 Construction and screening of *T. hirsuta* AH28-2 *lacA-*, *lacB-*, and *lacF-* silenced transformants.** (A) The transcription levels of *lacA* in *lacA-*silenced transformants exhibited 18% to 96% downregulation; (B) The transcription levels of *lacB* in *lacB-*silenced transformants exhibited 51% to 99% downregulation; (C) The transcription levels of *lacF* in *lacF-*silenced transformants exhibited 11% to 90% downregulation; (D-F) Determination of laccase activity in *lacA-*silenced transformants (74% reduction) (D), *lacB-*silenced transformants (56% reduction) (E), and *lacF-*silenced transformants (62% reduction) (F). (G-I) Native-PAGE analysis of laccase isozymes in *lacA-* (G), *lacB-* (H), and *lacF-* (I) silenced transformants. Data are presented as means ± standard deviation (n = 3). The data are analyzed using Student’s test (**p* < 0.05, ***p* < 0.01, ****p* < 0.001, or *****p* < 0.001).


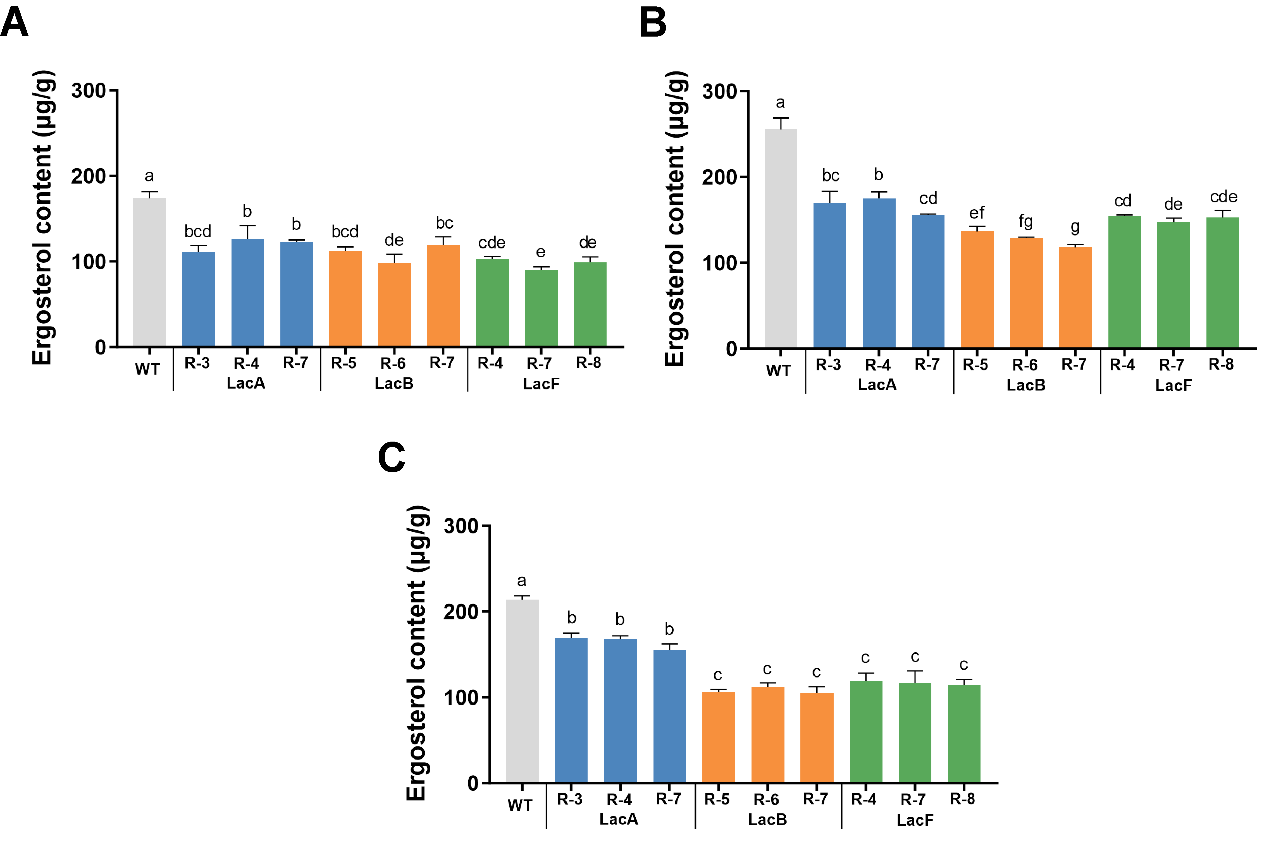


**Figure S10 Analysis of ergosterol content during wood degradation by gene-silenced transformants.** Ergosterol content of gene-silenced transformants grown on larch (A), poplar (B), and bamboo (C) for 20 days. Data are presented as means ± standard deviation (n = 3). Different letters indicate a significant difference at *p* < 0.05 according to Duncan’s multiple comparison.


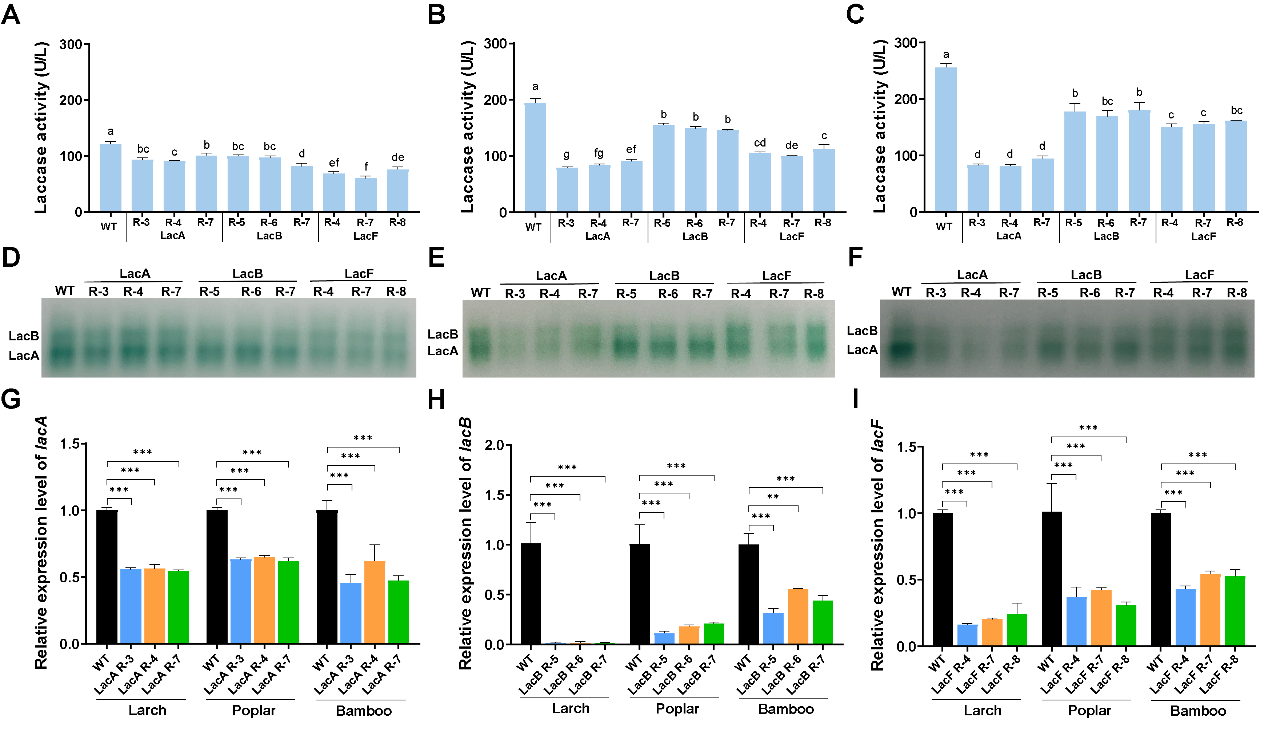


**Figure S11** **Analysis of laccase activity and expression levels during wood degradation by gene-silenced transformants.** Laccase activity of gene-silenced transformants grown on larch (A), poplar (B), and bamboo (C) for 20 days. Data are presented as means ± standard deviation (n = 3). Different letters indicate a significant difference at *p* < 0.05 according to Duncan’s multiple comparison. Native-PAGE analysis of laccase isozymes of gene-silenced transformants grown on the larch (D), poplar (E), and bamboo (F) for 20 days. Effects of the silencing transformants on transcript accumulation of *lacA* (G), *lacB* (H), and *lacF* (I) on wood medium at 20 days culture period using qRT-PCR. The data are analyzed using Student’s test (**p* < 0.05, ***p* < 0.01, ****p* < 0.001, or *****p* < 0.001).


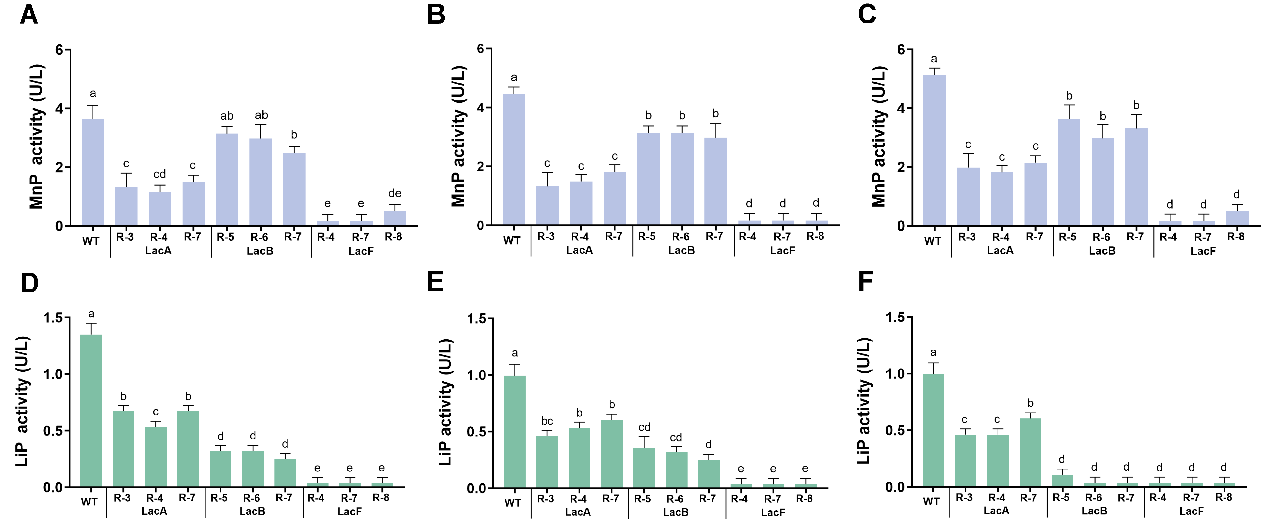


**Figure S12 Analysis of MnP and LiP activities during wood degradation by gene-silenced transformants.** MnP activity of gene-silenced transformants grown on larch (A), poplar (B), and bamboo (C) for 20 days. LiP activity of gene-silenced transformants grown on larch (D), poplar (E), and bamboo (F) for 20 days. Data are presented as means ± standard deviation (n = 3). Different letters indicate a significant difference at *p* < 0.05 according to Duncan’s multiple comparison.


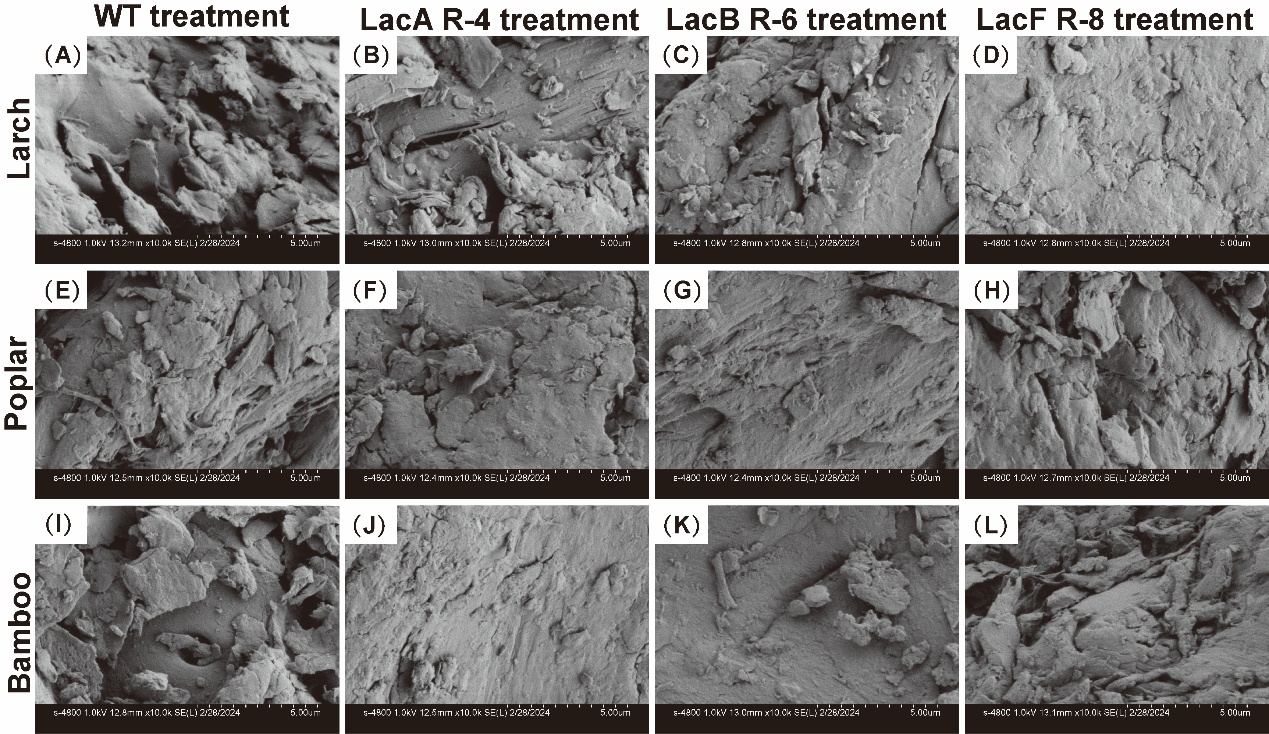


**Figure S13 Scanning electron microscopy photomicrograph of the surface of wood samples treated by wild type and gene-silenced transformants.**


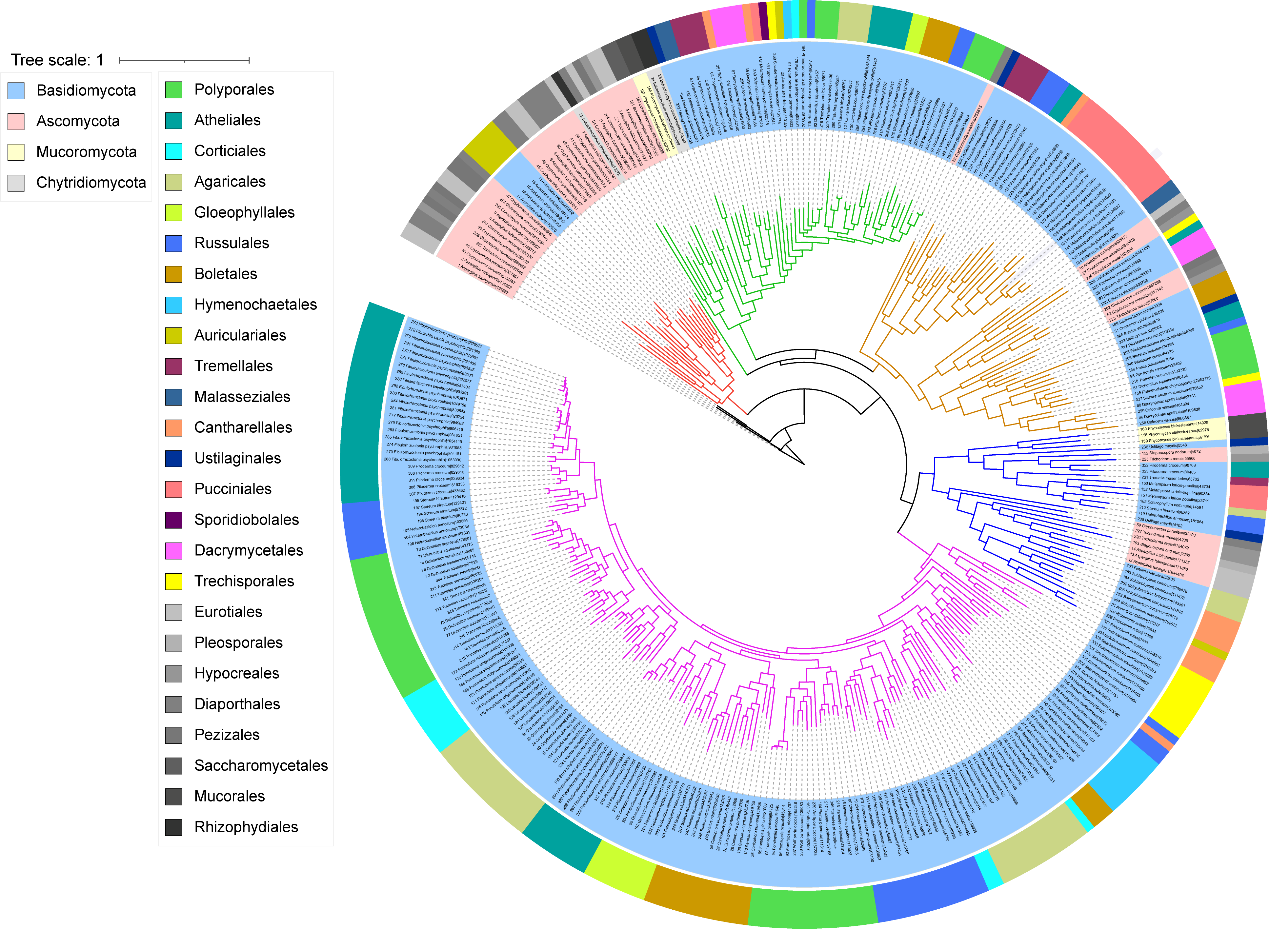


**Figure S14 The phylogenetic tree based on the amino acid sequence of laccase from 43 fungi.**


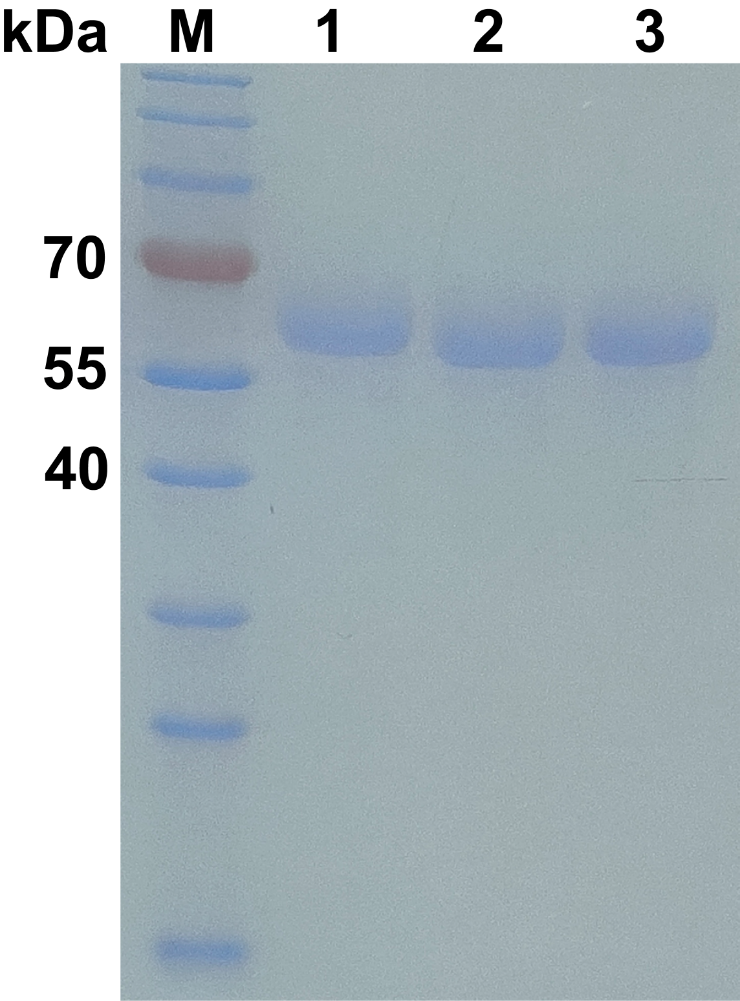


**Figure S15 SDS-PAGE of purified laccases.** Lanes: M: protein marker; 1: LacAnc178; 2: LacAnc169; 3: LacAnc160.


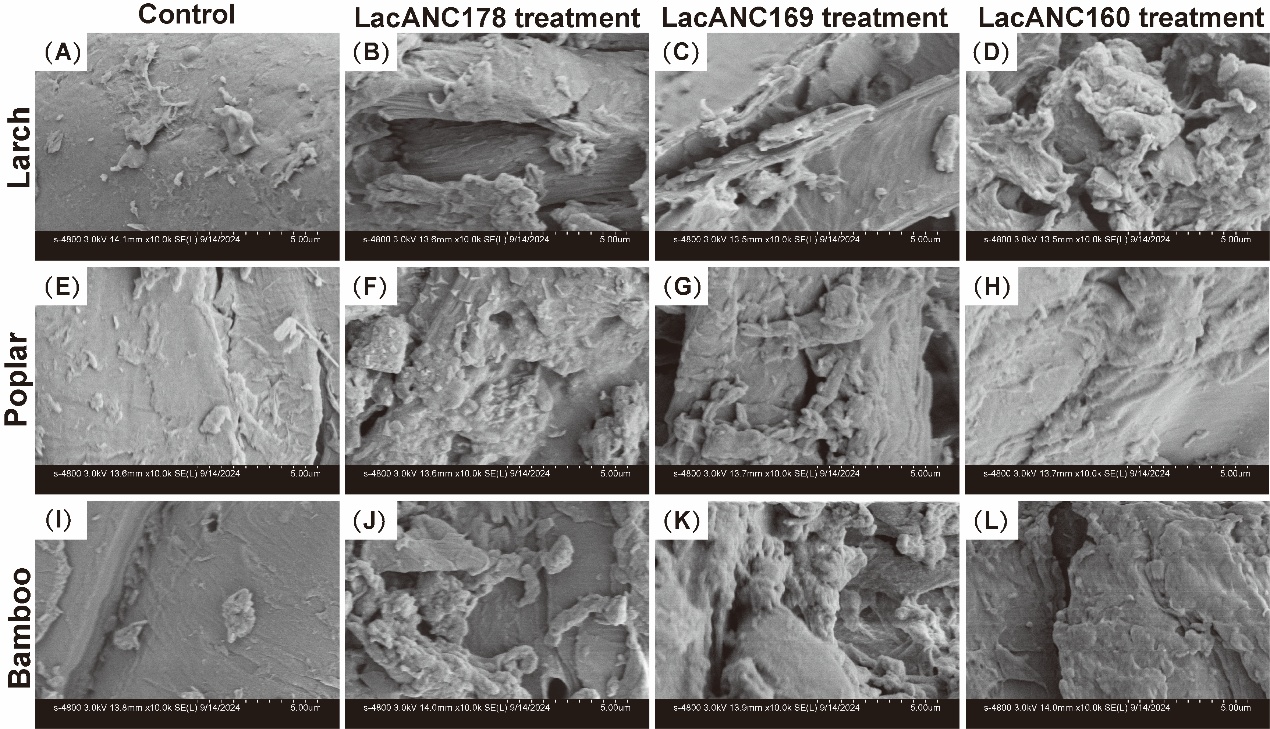


**Figure S16 Scanning electron microscopy photomicrograph of the surface of wood samples treated by recombinant ancestral laccases.**

**Table S1 The enzyme activity and protein content during purification.**

| **Laccase** | **Laccase activity（U/L）** | **Protein content (mg/L)** | **Specific activity (****U/mg)** |
| --- | --- | --- | --- |
| rLacA | 100500 | 430 | 233 |
| rLacB | 73778 | 478 | 154 |
| rLacC | 32073 | 435 | 73 |
| rLacD | 1010 | 176 | 5.7 |
| rLacE | 75349 | 287 | 262 |
| rLacF | 27524 | 200 | 137 |

**Table S2 Substrate specificity of the *T. hirsuta* AH28-2 laccases recombinantly expressed in *P. pastoris*.**

| **No.** | **Substrates** | **Recombinant laccases** | | | | | |
| --- | --- | --- | --- | --- | --- | --- | --- |
|  |  | **rLacA** | **rLacB** | **rLacC** | **rLacD** | **rLacE** | **rLac F** |
| 1 | Guaiacol | + | + | + | + | - | + |
| 2 | 5-Nitroguaiacol | +/- | - | - | - | - | + |
| 3 | Guaiacol glyceryl ether | - | - | - | - | - | - |
| 4 | Vanillic acid | + | + | + | + | + | + |
| 5 | *p*-Hydroxybenzoic acid | +/- | - | - | - | - | +/- |
| 6 | 4-Hydroxyphenylacetic acid | + | + | + | +/- | + | + |
| 7 | 4-Hydroxybenzaldehyde | + | - | + | + | - | + |
| 8 | 4-Hydroxyacetophenone | - | - | - | - | - | - |
| 9 | 2-(4-Hydroxyphenyl) ethanol | + | - | + | + | - | + |
| 10 | *p*-Methylphenol | + | + | + | - | - | + |
| 11 | Syringaldehyde | + | - | - | - | - | + |
| 12 | Syringic acid | + | + | + | + | + | + |
| 13 | Acetosyringone | + | + | + | + | + | + |

**Table S3 Redox potential of the purified laccases expressed in *P. pastoris*.**

| **Laccase** | **rLacA** | **rLacB** | **rLacC** | **rLacD** | **rLacE** | **rLacF** |
| --- | --- | --- | --- | --- | --- | --- |
| Redox potential (mV) | 680 | 500 | 620 | 600 | 560 | 730 |

**Table S4 Identification, structural classification, and relative abundance of larch lignin-derived pyrolysis products analyzed based on Py-GC/MS.** **The data represent the mean of three independent experiments.**

| **Compound** | **Retention time (min)** | **Structural feature** | **Relative content (%)** | | | |
| --- | --- | --- | --- | --- | --- | --- |
|  |  |  | **Control** | **rLacA** | **rLacB** | **rLacF** |
| Phenol, 2-methyl- | 9.286 | H | 0.40 | 0.56 | 0.64 | 0.58 |
| Phenol, 3-methyl- | 9.885 | H | 1.37 | 0.97 | 0.87 | 1.01 |
| Phenol, 2-methoxy- | 10.324 | G | 5.59 | 3.98 | 4.42 | 3.58 |
| Phenol, 2,4-dimethyl- | 11.934 | H | 0.52 | 0.40 | 0.47 | 0.47 |
| Creosol | 13.311 | G | 5.18 | 4.62 | 5.01 | 4.25 |
| Phenol, 4-ethyl-2-methoxy- | 15.725 | G | 2.13 | 1.53 | 1.51 | 1.49 |
| 2-Methoxy-4-vinyl phenol | 16.768 | G | 5.65 | 5.00 | 5.02 | 4.58 |
| Eugenol | 17.918 | G | 2.37 | 1.61 | 1.55 | 1.52 |
| Vanillin | 19.109 | G | 1.62 | 1.59 | 1.55 | 1.57 |
| Isoeugenol | 19.273 | G | 1.37 | 0.94 | 1.00 | 0.92 |
| trans-Isoeugenol | 20.428 | G | 5.69 | 5.20 | 5.27 | 4.66 |
| 2-methoxy-4-propyl-phenol | 20.655 | G | 1.27 | 1.02 | 0.98 | 0.98 |
| Total | | G | 30.88 | 25.48 | 26.31 | 23.55 |
|  |  | H | 2.29 | 1.93 | 1.98 | 2.07 |

**Table S5** **Identification, structural classification, and relative abundance of poplar lignin-derived pyrolysis products analyzed based on Py-GC/MS. The data represent the mean of three independent experiments.**

| **Compound** | **Retention time (min)** | **Structural feature** | **Relative content (%)** | | | |
| --- | --- | --- | --- | --- | --- | --- |
|  |  |  | **Control** | **rLacA** | **rLacB** | **rLacF** |
| Phenol | 7.295 | H | 2.95 | 2.77 | 2.72 | 2.69 |
| Phenol, 2-methyl- | 9.259 | H | 0.25 | 0.22 | 0.25 | 0.16 |
| Phenol, 2-methoxy- | 10.281 | G | 2.22 | 2.07 | 2.07 | 2.06 |
| Phenol, 2,4-dimethyl- | 11.912 | H | 0.19 | 0.18 | 0.15 | 0.18 |
| Creosol | 13.252 | G | 2.72 | 2.54 | 2.58 | 2.17 |
| Phenol, 4-ethyl-2-methoxy- | 15.704 | G | 1.17 | 0.89 | 0.86 | 0.91 |
| 2-Methoxy-4-vinylphenol | 16.715 | G | 3.12 | 2.40 | 2.53 | 2.37 |
| Phenol, 2,6-dimethoxy- | 17.780 | S | 4.90 | 3.41 | 3.93 | 4.07 |
| Eugenol | 17.901 | G | 0.99 | 0.82 | 0.81 | 1.04 |
| Vanillin | 19.199 | G | 1.24 | 0.92 | 0.98 | 1.05 |
| Isoeugenol | 19.252 | G | 0.62 | 0.48 | 0.55 | 0.61 |
| 2,6-Dimethoxy-4-methylphenol | 20.327 | S | 5.50 | 3.02 | 2.62 | 4.23 |
| Phenol, 2-methoxy-4-propyl- | 20.639 | G | 0.43 | 0.31 | 0.31 | 0.44 |
| Phenol, 2,6-dimethoxy-4-(2-propenyl)- | 24.208 | S | 1.62 | 1.21 | 1.26 | 1.31 |
| Benzaldehyde, 4-hydroxy-3,5-dimethoxy- | 25.569 | S | 0.42 | 0.38 | 0.44 | 0.37 |
| (E)-2,6-Dimethoxy-4-(prop-1-en-1-yl) phenol | 26.527 | S | 2.35 | 1.89 | 2.12 | 2.08 |
| Total | | G | 12.52 | 10.43 | 10.69 | 10.65 |
|  |  | S | 14.79 | 9.91 | 10.37 | 12.06 |
|  |  | H | 3.39 | 3.17 | 3.12 | 3.03 |
|  |  | S/G | 1.18 | 0.95 | 0.97 | 1.13 |

**Table S6 Identification, structural classification and relative abundance of bamboo lignin-derived pyrolysis products by Py-GC/MS. The data represent the mean of three independent experiments.**

| **Compound** | **Retention time (min)** | **Structural feature** | **Relative content (%)** | | | |
| --- | --- | --- | --- | --- | --- | --- |
|  |  |  | **Control** | **rLacA** | **rLacB** | **rLacF** |
| Phenol | 7.385 | H | 1.51 | 1.16 | 1.18 | 1.16 |
| Phenol, 2-methyl- | 9.328 | H | 0.36 | 0.45 | 0.54 | 0.76 |
| Phenol, 3-methyl- | 9.921 | H | 2.53 | 1.19 | 1.44 | 1.59 |
| Phenol, 2-methoxy- | 10.345 | G | 2.73 | 1.93 | 2.25 | 2.62 |
| Phenol, 2,4-dimethyl- | 11.965 | H | 0.34 | 0.28 | 0.31 | 0.59 |
| Creosol | 13.310 | G | 3.24 | 2.39 | 2.83 | 2.83 |
| Phenol, 4-ethyl-2-methoxy- | 15.752 | G | 1.03 | 0.91 | 1.03 | 0.86 |
| 2-Methoxy-4-vinylphenol | 16.789 | G | 3.18 | 3.57 | 2.96 | 3.02 |
| Phenol, 2,6-dimethoxy- | 17.838 | S | 8.61 | 6.09 | 6.07 | 7.26 |
| Eugenol | 17.938 | G | 0.71 | 0.62 | 0.71 | 0.57 |
| Vanillin | 19.119 | G | 0.92 | 0.91 | 0.89 | 0.57 |
| 2,6-Dimethoxy-4-methylphenol | 20.369 | S | 6.27 | 4.09 | 4.27 | 5.91 |
| Isoeugenol | 20.427 | G | 1.72 | 1.61 | 1.66 | 1.29 |
| Phenol, 2-methoxy-4-propyl- | 20.671 | G | 0.54 | 0.41 | 0.52 | 0.36 |
| Phenol, 2,6-dimethoxy-4-(2-propenyl)- | 24.229 | S | 1.33 | 1.03 | 1.11 | 1.10 |
| Benzaldehyde, 4-hydroxy-3,5-dimethoxy- | 25.590 | S | 0.42 | 0.33 | 0.36 | 0.38 |
| (E)-2,6-Dimethoxy-4-(prop-1-en-1-yl) phenol | 26.543 | S | 2.02 | 1.77 | 1.71 | 1.02 |
| Total | | G | 14.06 | 12.35 | 12.85 | 12.13 |
|  |  | S | 18.64 | 13.31 | 13.52 | 15.66 |
|  |  | H | 4.73 | 3.08 | 3.46 | 4.10 |
|  |  | S/G | 1.33 | 1.08 | 1.05 | 1.29 |

**Table S7 Characteristic peak location and signal assignment in FTIR spectra of wood.**

| **Wavenumber（cm^-1^）** | **Assignment** |
| --- | --- |
| 3350-3450 | O-H stretch |
| 1595-1605 | Aromatic skeletal vibrations plus C=O stretch |
| 1505-1515 | Aromatic skeletal vibrations |
| 1420-1430 | Aromatic skeletal vibrations combined with C-H in -plane deformation |
| 1325-1330 | S ring breathing with C-O stretching vibration |
| 1266-1270 | G ring plus C=O stretch |
| 1140 | Aromatic C-H in-plane deformation; typical for G units |
| 1120-1125 | Aromatic C-H in-plane deformation, typical for S units |
| 1030-1040 | Aromatic C-H in-plane deformation |
| 834-835 | C-H out-of-plane in positions 2 and 6 of S and in all positions of H units |

**Table S8 Identification, structural classification and relative abundance of lignin-derived pyrolysis products by Py-GC/MS. WT and gene-silenced transformants-treated larch samples after 20 days of treatment were used. The data represent the mean of three independent experiments.**

| **Compound** | **Structural feature** | **WT** | **LacA** | | | **LacB** | | | **LacF** | | |
| --- | --- | --- | --- | --- | --- | --- | --- | --- | --- | --- | --- |
|  |  |  | **R-3** | **R-4** | **R-7** | **R-5** | **R-6** | **R-7** | **R-4** | **R-7** | **R-8** |
| Phenol, 2-methyl- | H | 0.28 | 0.46 | 0.14 | 0.58 | 0.46 | 0.69 | 0.53 | 0.41 | 0.39 | 0.41 |
| Phenol, 3-methyl- | H | 0.87 | 1.12 | 1.01 | 1.12 | 1.01 | 0.72 | 0.88 | 0.76 | 0.72 | 0.78 |
| Phenol, 2-methoxy- | G | 3.47 | 3.99 | 4.15 | 5.23 | 4.37 | 5.55 | 4.62 | 3.69 | 3.76 | 3.74 |
| Phenol, 2,4-dimethyl- | H | 0.19 | 0.26 | 0.26 | 0.25 | 0.19 | 0.47 | 0.46 | 0.13 | 0.19 | 0.20 |
| Creosol | G | 5.79 | 7.51 | 7.34 | 6.22 | 5.73 | 6.29 | 5.83 | 6.69 | 6.55 | 6.65 |
| Phenol, 4-ethyl-2-methoxy- | G | 1.76 | 3.03 | 2.79 | 1.61 | 1.75 | 1.88 | 1.71 | 2.00 | 1.99 | 1.70 |
| 2-Methoxy-4-vinyl phenol | G | 5.08 | 5.48 | 5.39 | 5.26 | 5.73 | 5.56 | 5.57 | 5.50 | 5.55 | 5.67 |
| Eugenol | G | 1.24 | 1.28 | 0.81 | 1.68 | 1.51 | 1.33 | 1.41 | 1.42 | 1.87 | 2.04 |
| Vanillin | G | 1.53 | 1.22 | 1.81 | 1.71 | 1.39 | 1.13 | 1.21 | 2.14 | 2.01 | 1.97 |
| Isoeugenol | G | 1.19 | 0.65 | 0.29 | 0.69 | 0.90 | 0.69 | 0.72 | 1.83 | 1.49 | 1.33 |
| trans-Isoeugenol | G | 4.26 | 3.25 | 2.56 | 2.86 | 4.50 | 4.17 | 4.21 | 4.48 | 4.75 | 4.75 |
| 2-methoxy-4-propyl-phenol | G | 1.23 | 1.17 | 2.30 | 1.47 | 0.95 | 1.16 | 1.15 | 1.33 | 1.19 | 1.25 |
| Total | G | 25.55 | 27.58 | 27.44 | 26.73 | 26.83 | 27.76 | 26.43 | 29.08 | 29.16 | 29.10 |
|  | H | 1.34 | 1.84 | 1.41 | 1.95 | 1.66 | 1.88 | 1.87 | 1.30 | 1.30 | 1.39 |

**Table S9 Identification, structural classification and relative abundance of lignin-derived pyrolysis products by Py-GC/MS. WT and gene-silenced transformants-treated poplar samples after 20 days of treatment were used. The data represent the mean of three independent experiments.**

| **Compound** | **Structural feature** | **WT** | **LacA** | | | **LacB** | | | **LacF** | | |
| --- | --- | --- | --- | --- | --- | --- | --- | --- | --- | --- | --- |
|  |  |  | **R-3** | **R-4** | **R-7** | **R-5** | **R-6** | **R-7** | **R-4** | **R-7** | **R-8** |
| Phenol | H | 2.51 | 2.50 | 2.57 | 2.61 | 2.55 | 2.50 | 2.25 | 2.34 | 2.17 | 2.20 |
| Phenol, 2-methyl- | H | 0.44 | 0.57 | 0.43 | 0.39 | 0.34 | 0.45 | 0.56 | 0.34 | 0.59 | 0.53 |
| Phenol, 2-methoxy- | G | 2.36 | 2.23 | 2.32 | 2.30 | 2.56 | 2.11 | 2.78 | 2.61 | 2.66 | 2.82 |
| Phenol, 2,4-dimethyl- | H | 0.08 | 0.10 | 0.09 | 0.09 | 0.14 | 0.17 | 0.19 | 0.27 | 0.26 | 0.22 |
| Creosol | G | 1.94 | 3.07 | 2.94 | 2.96 | 2.88 | 3.14 | 3.14 | 2.99 | 2.77 | 2.91 |
| Phenol, 4-ethyl-2-methoxy- | G | 0.68 | 1.27 | 1.25 | 1.23 | 1.37 | 1.43 | 1.08 | 1.16 | 1.33 | 1.06 |
| 2-Methoxy-4-vinylphenol | G | 2.99 | 3.17 | 3.04 | 2.98 | 2.65 | 2.53 | 2.49 | 2.90 | 2.78 | 2.66 |
| Phenol, 2,6-dimethoxy- | S | 3.36 | 4.81 | 4.98 | 4.85 | 5.52 | 5.75 | 5.84 | 4.22 | 4.26 | 4.32 |
| Eugenol | G | 0.50 | 0.38 | 0.39 | 0.38 | 0.22 | 0.37 | 0.28 | 0.19 | 0.16 | 0.17 |
| Vanillin | G | 0.80 | 0.33 | 0.52 | 0.49 | 0.86 | 0.96 | 0.95 | 0.60 | 0.58 | 0.64 |
| Isoeugenol | G | 0.41 | 0.32 | 0.41 | 0.37 | 0.42 | 0.55 | 0.37 | 0.36 | 0.46 | 0.49 |
| 2,6-Dimethoxy-4-methylphenol | S | 4.17 | 5.35 | 5.42 | 5.29 | 5.74 | 5.85 | 5.91 | 5.08 | 4.89 | 4.56 |
| 2-methoxy-4-propyl-Phenol | G | 0.77 | 0.23 | 0.22 | 0.16 | 0.27 | 0.47 | 0.33 | 0.82 | 0.68 | 0.55 |
| Phenol, 2,6-dimethoxy-4-(2-propenyl)- | S | 1.54 | 1.32 | 1.43 | 1.16 | 0.55 | 0.54 | 0.65 | 1.20 | 1.10 | 1.01 |
| Benzaldehyde, 4-hydroxy-3,5-dimethoxy- | S | 0.46 | 0.62 | 0.58 | 0.54 | 0.46 | 0.36 | 0.27 | 0.53 | 0.54 | 0.43 |
| (E)-2,6-Dimethoxy-4-(prop-1-en-1-yl) phenol | S | 1.22 | 1.94 | 1.83 | 1.99 | 1.25 | 1.13 | 0.84 | 1.31 | 1.36 | 1.47 |
| Total | G | 10.45 | 11.00 | 11.09 | 10.87 | 11.23 | 11.56 | 11.42 | 11.63 | 11.42 | 11.30 |
|  | S | 10.75 | 14.04 | 14.24 | 13.83 | 13.52 | 13.63 | 13.51 | 12.34 | 12.15 | 11.79 |
|  | H | 3.03 | 3.17 | 3.09 | 3.09 | 3.03 | 3.12 | 3.00 | 2.95 | 3.02 | 2.95 |
|  | S/G | 1.03 | 1.28 | 1.28 | 1.27 | 1.20 | 1.18 | 1.19 | 1.06 | 1.06 | 1.04 |

**Table S10 Identification, structural classification and relative abundance of lignin-derived pyrolysis products by Py-GC/MS. WT and gene-silenced transformants-treated bamboo samples after 20 days of treatment were used. The data represent the mean of three independent experiments.**

| **Compound** | **Structural feature** | **WT** | **LacA** | | | **LacB** | | | **LacF** | | |
| --- | --- | --- | --- | --- | --- | --- | --- | --- | --- | --- | --- |
|  |  |  | **R-3** | **R-4** | **R-7** | **R-5** | **R-6** | **R-7** | **R-4** | **R-7** | **R-8** |
| Phenol | H | 1.51 | 1.71 | 1.60 | 1.43 | 1.80 | 1.76 | 1.84 | 1.69 | 1.86 | 1.46 |
| Phenol, 2-methyl- | H | 0.45 | 0.62 | 0.62 | 0.60 | 0.50 | 0.48 | 0.49 | 0.64 | 0.63 | 0.63 |
| Phenol, 3-methyl- | H | 1.50 | 2.09 | 1.93 | 1.84 | 1.66 | 1.82 | 1.68 | 1.71 | 1.98 | 1.78 |
| Phenol, 2-methoxy- | G | 3.12 | 4.10 | 4.16 | 3.96 | 3.97 | 4.05 | 4.03 | 3.39 | 3.46 | 3.52 |
| Phenol, 2,4-dimethyl- | H | 0.37 | 0.67 | 0.67 | 0.67 | 0.11 | 0.13 | 0.10 | 0.31 | 0.42 | 0.55 |
| Creosol | G | 2.59 | 2.84 | 2.88 | 2.76 | 2.94 | 3.28 | 2.99 | 3.33 | 3.19 | 2.84 |
| Phenol, 4-ethyl-2-methoxy- | G | 1.27 | 1.11 | 1.16 | 1.06 | 1.13 | 1.34 | 1.19 | 1.18 | 1.25 | 1.30 |
| 2-Methoxy-4-vinylphenol | G | 3.27 | 3.54 | 3.01 | 3.29 | 3.29 | 3.75 | 3.25 | 3.00 | 2.87 | 3.05 |
| Phenol, 2,6-dimethoxy- | S | 6.29 | 7.48 | 7.14 | 6.62 | 7.80 | 8.46 | 8.24 | 7.12 | 6.86 | 6.75 |
| 2,6-Dimethoxy-4-methylphenol | S | 5.17 | 6.17 | 5.97 | 5.95 | 6.12 | 6.27 | 6.27 | 6.16 | 6.13 | 5.85 |
| 2-methoxy-4-propyl-phenol | G | 0.28 | 0.21 | 0.30 | 0.27 | 0.26 | 0.16 | 0.18 | 0.12 | 0.22 | 0.23 |
| Phenol, 2,6-dimethoxy-4-(2-propenyl)- | S | 0.83 | 0.88 | 0.95 | 1.09 | 0.83 | 1.03 | 0.96 | 0.88 | 0.86 | 0.89 |
| Benzaldehyde, 4-hydroxy-3,5-dimethoxy- | S | 0.42 | 0.50 | 0.62 | 0.58 | 0.40 | 0.66 | 0.54 | 0.30 | 0.25 | 0.31 |
| (E)-2,6-Dimethoxy-4-(prop-1-en-1-yl) phenol | S | 1.19 | 1.66 | 1.80 | 1.88 | 1.74 | 1.89 | 1.78 | 0.88 | 0.92 | 1.09 |
| Total | G | 10.53 | 11.80 | 11.51 | 11.34 | 11.59 | 12.58 | 11.64 | 11.02 | 10.99 | 10.94 |
|  | S | 13.90 | 16.69 | 16.48 | 16.12 | 16.89 | 18.31 | 17.79 | 15.34 | 15.02 | 14.89 |
|  | H | 3.83 | 5.09 | 4.82 | 4.54 | 4.07 | 4.19 | 4.11 | 4.35 | 4.89 | 4.42 |
|  | S/G | 1.32 | 1.41 | 1.43 | 1.42 | 1.46 | 1.46 | 1.53 | 1.39 | 1.37 | 1.36 |

**Table S11 Relative transmittance** ^a^ **of infrared spectra of larch lignin degraded by WT and silencing transformants.**

| **Wavenumber (cm^-1^)** | **Relative transmittance** | | | | |
| --- | --- | --- | --- | --- | --- |
|  | **Control** | **WT** | **LacA R-4** | **LacB R-6** | **LacF R-8** |
| 3370 | 0.09 | 0.46 | 0.23 | 0.21 | 0.14 |
| 1605 | 1.17 | 1.79 | 1.57 | 1.42 | 1.18 |
| 1510 | 1.00 | 1.00 | 1.00 | 1.00 | 1.00 |
| 1425 | 0.70 | 0.94 | 0.93 | 0.92 | 0.88 |
| 1266 | 0.52 | 0.86 | 0.80 | 0.74 | 0.71 |
| 1033 | 0.11 | 0.39 | 0.19 | 0.17 | 0.12 |

^a^ The relative transmittance of each peak was calculated with the transmittance of the aromatic skeletal vibration at 1510 cm^-1^ as the standard.

**Table S12 Relative transmittance** ^a^ **of infrared spectra of poplar lignin degraded by WT and silencing transformants.**

| **Wavenumber (cm^-1^)** | **Relative transmittance** | | | | |
| --- | --- | --- | --- | --- | --- |
|  | **Control** | **WT** | **LacA R-4** | **LacB R-6** | **LacF R-8** |
| 3370 | 0.09 | 0.29 | 0.19 | 0.25 | 0.27 |
| 1600 | 0.89 | 1.07 | 1.04 | 1.04 | 1.07 |
| 1510 | 1.00 | 1.00 | 1.00 | 1.00 | 1.00 |
| 1330 | 0.46 | 0.79 | 0.68 | 0.64 | 0.76 |
| 1266 | 0.33 | 0.67 | 0.54 | 0.57 | 0.63 |
| 1125 | 0.12 | 0.36 | 0.30 | 0.23 | 0.32 |
| 834 | 2.11 | 3.45 | 2.60 | 2.11 | 3.36 |

^a^ The relative transmittance of each peak was calculated with the transmittance of the aromatic skeletal vibration at 1510 cm^-1^ as the standard.

**Table S13 Relative transmittance** ^a^ **of infrared spectra of bamboo lignin degraded by WT and silencing transformants.**

| **Wavenumber (cm^-1^)** | **Relative transmittance** | | | | |
| --- | --- | --- | --- | --- | --- |
|  | **Control** | **WT** | **LacA R-4** | **LacB R-6** | **LacF R-8** |
| 3370 | 0.19 | 0.31 | 0.21 | 0.17 | 0.29 |
| 1600 | 0.74 | 0.99 | 0.79 | 0.78 | 0.96 |
| 1510 | 1.00 | 1.00 | 1.00 | 1.00 | 1.00 |
| 1330 | 0.60 | 0.83 | 0.63 | 0.61 | 0.81 |
| 1266 | 0.45 | 0.70 | 0.36 | 0.42 | 0.65 |
| 1125 | 0.20 | 0.42 | 0.26 | 0.21 | 0.37 |
| 834 | 2.87 | 4.52 | 2.27 | 1.97 | 3.64 |

^a^ The relative transmittance of each peak was calculated with the transmittance of the aromatic skeletal vibration at 1510 cm^-1^ as the standard.

**Table S14 Identification, structural classification and relative abundance of larch lignin-derived pyrolysis products by Py-GC/MS. The data represent the mean of three independent experiments.**

| **Compound** | **Structural feature** | **Relative content (%)** | | |
| --- | --- | --- | --- | --- |
|  |  | **LacAnc178** | **LacAnc169** | **LacAnc160** |
| Phenol, 2-methyl- | H | 0.81 | 0.81 | 0.87 |
| Phenol, 3-methyl- | H | 0.50 | 0.50 | 0.45 |
| Phenol, 2-methoxy- | G | 5.97 | 5.62 | 5.41 |
| Phenol, 2,4-dimethyl- | H | 0.21 | 0.22 | 0.17 |
| Creosol | G | 5.09 | 4.69 | 4.64 |
| Phenol, 4-ethyl-2-methoxy- | G | 4.08 | 3.57 | 3.28 |
| 2-Methoxy-4-vinyl phenol | G | 5.71 | 5.4 | 4.91 |
| Eugenol | G | 0.97 | 0.91 | 0.69 |
| Vanillin | G | 0.79 | 0.72 | 0.58 |
| Isoeugenol | G | 0.86 | 0.85 | 0.61 |
| trans-Isoeugenol | G | 3.98 | 3.85 | 3.69 |
| Phenol, 2-methoxy-4-propyl- | G | 1.50 | 1.01 | 1.10 |
| Total | G | 28.95 | 26.62 | 24.91 |
|  | H | 1.52 | 1.53 | 1.49 |

**Table S15 Identification, structural classification and relative abundance of poplar lignin-derived pyrolysis products by Py-GC/MS. The data represent the mean of three independent experiments.**

| **Compound** | **Structural feature** | **Relative content (%)** | | |
| --- | --- | --- | --- | --- |
|  |  | **LacAnc178** | **LacAnc169** | **LacAnc160** |
| Phenol | H | 3.12 | 2.87 | 1.66 |
| Phenol, 2-methyl- | H | 0.22 | 0.11 | 0.12 |
| Phenol, 2-methoxy- | G | 2.67 | 2.75 | 2.95 |
| Phenol, 2,4-dimethyl- | H | 0.18 | 0.10 | 0.10 |
| Creosol | G | 1.82 | 1.82 | 1.94 |
| Phenol, 4-ethyl-2-methoxy- | G | 1.59 | 1.16 | 1.67 |
| 2-Methoxy-4-vinylphenol | G | 3.03 | 3.60 | 3.25 |
| Phenol, 2,6-dimethoxy- | S | 4.70 | 6.41 | 7.38 |
| Eugenol | G | 0.30 | 0.24 | 0.32 |
| Vanillin | G | 0.32 | 0.35 | 0.31 |
| Isoeugenol | G | 0.52 | 0.60 | 0.33 |
| 2,6-Dimethoxy-4-methylphenol | S | 1.76 | 3.93 | 4.47 |
| Phenol, 2-methoxy-4-propyl- | G | 0.20 | 0.30 | 0.23 |
| Phenol, 2,6-dimethoxy-4-(2-propenyl)- | S | 0.99 | 1.41 | 0.82 |
| Benzaldehyde, 4-hydroxy-3,5-dimethoxy- | S | 0.18 | 0.37 | 0.33 |
| (E)-2,6-Dimethoxy-4-(prop-1-en-1-yl) phenol | S | 1.56 | 1.63 | 1.46 |
| Total | G | 10.45 | 10.82 | 11.00 |
|  | S | 9.19 | 13.75 | 14.46 |
|  | H | 3.52 | 3.08 | 1.88 |
|  | S/G | 0.88 | 1.27 | 1.32 |

**Table S16 Identification, structural classification and relative abundance of bamboo lignin-derived pyrolysis products by Py-GC/MS. The data represent the mean of three independent experiments.**

| **Compound** | **Structural feature** | **Relative content (%)** | | |
| --- | --- | --- | --- | --- |
|  |  | **LacAnc178** | **LacAnc169** | **LacAnc160** |
| Phenol | H | 1.54 | 1.35 | 0.92 |
| Phenol, 2-methyl- | H | 1.40 | 1.30 | 0.85 |
| Phenol, 3-methyl- | H | 1.30 | 1.38 | 0.99 |
| Phenol, 2-methoxy- | G | 3.31 | 3.09 | 1.93 |
| Phenol, 2,4-dimethyl- | H | 0.28 | 0.28 | 0.17 |
| Creosol | G | 2.15 | 2.00 | 2.02 |
| Phenol, 4-ethyl-2-methoxy- | G | 0.94 | 1.07 | 0.59 |
| 2-Methoxy-4-vinylphenol | G | 2.95 | 3.02 | 3.01 |
| Phenol, 2,6-dimethoxy- | S | 5.64 | 6.61 | 6.21 |
| Vanillin | G | 0.17 | 0.16 | 0.67 |
| 2,6-Dimethoxy-4-methyl phenol | S | 2.62 | 2.78 | 3.45 |
| Isoeugenol | G | 1.06 | 1.01 | 1.43 |
| Phenol, 2-methoxy-4-propyl- | G | 0.21 | 0.10 | 0.28 |
| Phenol, 2,6-dimethoxy-4-(2-propenyl)- | S | 0.57 | 0.61 | 0.95 |
| Benzaldehyde, 4-hydroxy-3,5-dimethoxy- | S | 0.36 | 0.36 | 0.56 |
| (E)-2,6-Dimethoxy-4-(prop-1-en-1-yl) phenol | S | 0.76 | 1.21 | 1.07 |
| Total | G | 10.79 | 10.45 | 9.93 |
|  | S | 9.95 | 11.57 | 12.24 |
|  | H | 4.52 | 4.31 | 2.93 |
|  | S/G | 0.92 | 1.11 | 1.23 |

**Table S17 Infrared spectral relative transmittance** ^a^ **of larch lignin in control and ancestral laccase treatment groups.**

| **Wavenumber (cm^-1^)** | **Relative transmittance** | | | |
| --- | --- | --- | --- | --- |
|  | **Control** | **LacAnc178** | **LacAnc169** | **LacAnc160** |
| 3370 | 0.09 | 0.19 | 0.21 | 0.29 |
| 1510 | 1.00 | 1.00 | 1.00 | 1.00 |
| 1425 | 0.70 | 0.83 | 0.89 | 0.92 |
| 1266 | 0.52 | 0.70 | 0.70 | 0.78 |
| 1033 | 0.11 | 0.15 | 0.22 | 0.26 |

^a^ The relative transmittance of each peak was calculated with the transmittance of the aromatic skeletal vibration at 1510 cm^-1^ as the standard.

**Table S18 Infrared spectral relative transmittance** ^a^ **of poplar lignin in control and ancestral laccase treatment groups.**

| **Wavenumber (cm^-1^)** | **Relative transmittance** | | | |
| --- | --- | --- | --- | --- |
|  | **Control** | **LacAnc178** | **LacAnc169** | **LacAnc160** |
| 1600 | 0.89 | 1.05 | 1.04 | 1.00 |
| 1510 | 1.00 | 1.00 | 1.00 | 1.00 |
| 1330 | 0.46 | 0.79 | 0.76 | 0.74 |
| 1266 | 0.33 | 0.60 | 0.63 | 0.67 |
| 1125 | 0.12 | 0.35 | 0.32 | 0.30 |
| 834 | 2.11 | 2.18 | 2.60 | 2.11 |

^a^ The relative transmittance of each peak was calculated with the transmittance of the aromatic skeletal vibration at 1510 cm^-1^ as the standard.

**Table S19 Infrared spectral relative transmittance** ^a^ **of bamboo lignin in control and ancestral laccase treatment groups.**

| **Wavenumber (cm^-1^)** | **Relative transmittance** | | | |
| --- | --- | --- | --- | --- |
|  | **Control** | **LacAnc178** | **LacAnc169** | **LacAnc160** |
| 1600 | 0.74 | 0.94 | 0.93 | 0.85 |
| 1510 | 1.00 | 1.00 | 1.00 | 1.00 |
| 1330 | 0.60 | 0.74 | 0.81 | 0.69 |
| 1266 | 0.45 | 0.57 | 0.65 | 0.50 |
| 1125 | 0.20 | 0.27 | 0.37 | 0.21 |
| 834 | 2.87 | 3.03 | 2.91 | 1.98 |

^a^ The relative transmittance of each peak was calculated with the transmittance of the aromatic skeletal vibration at 1510 cm^-1^ as the standard.

**Table S20 Classification and ecological habits of 43 fungi.**

| **No.** | **Phylum** | **Class** | **Genus/Species** | **Ecology** | **Availability** |
| --- | --- | --- | --- | --- | --- |
| 1 | Basidiomycota | Agaricomycetes | *Trametes versicolor* | White rot | http:// mycocosm.jgi.doe.gov/Trave1/Trave1.home.html |
| 2 |  |  | *Trametes hirsuta* | White rot | https://www.ncbi.nlm.nih.gov/datasets/genome/GCA_001304625.1/ |
| 3 |  |  | *Dichomitus squalens* | White rot | http:// mycocosm.jgi.doe.gov/Dicsq1/Dicsq1.home.html |
| 4 |  |  | *Wolfiporia cocos* | Brown rot | http:// mycocosm.jgi.doe.gov/Wolco1/Wolco1.home.html |
| 5 |  |  | *Fomitopsis pinicola* | Brown rot | http:// mycocosm.jgi.doe.gov/Fompi3/Fompi3.home.html |
| 6 |  |  | *Phanerodontia chrysosporium* | White rot | http:// mycocosm.jgi.doe.gov/Phchr2/Phchr2.home.html |
| 7 |  |  | *Rhodonia placenta* | Brown rot | http:// mycocosm.jgi.doe.gov/Pospl1/Pospl1.home.html |
| 8 |  |  | *Coprinopsis cinerea* | Straw rot | https://mycocosm.jgi.doe.gov/Copci1/Copci1.home.html |
| 9 |  |  | *Laccaria bicolor* | Symbiotic | https://mycocosm.jgi.doe.gov/Lacbi2/Lacbi2.home.html |
| 10 |  |  | *Schizophyllum commune* | White rot | https://mycocosm.jgi.doe.gov/Schco3/Schco3.home.html |
| 11 |  |  | *Fistulina hepatica* | Brown rot | https://mycocosm.jgi.doe.gov/Fishe1/Fishe1.home.html |
| 12 |  |  | *Coniophora puteana* | Brown rot | https://mycocosm.jgi.doe.gov/Conpu1/Conpu1.home.html |
| 13 |  |  | *Serpula lacrymans* | Brown rot | https://mycocosm.jgi.doe.gov/SerlaS7_3_2/SerlaS7_3_2.home.html |
| 14 |  |  | *Boletus edulis* | Symbiotic | https://mycocosm.jgi.doe.gov/Boled5/Boled5.home.html |
| 15 |  |  | *Stereum hirsutum* | White rot | https://mycocosm.jgi.doe.gov/Stehi1/Stehi1.home.html |
| 16 |  |  | *Heterobasidion annosum* | White rot | https://mycocosm.jgi.doe.gov/Hetan2/Hetan2.home.html |
| 17 |  |  | *Auricularia delicata* | White rot | https://mycocosm.jgi.doe.gov/Aurde3_1/Aurde3_1.home.html |
| 18 |  |  | *Fomitiporia mediterranea* | White rot | https://mycocosm.jgi.doe.gov/Fomme1/Fomme1.home.html |
| 19 |  |  | *Gloeophyllum trabeum* | Brown rot | https://mycocosm.jgi.doe.gov/Glotr1_1/Glotr1_1.home.html |
| 20 |  |  | *Neolentinus lepideus* | Brown rot | https://mycocosm.jgi.doe.gov/Neole1/Neole1.home.html |
| 21 |  |  | *Punctularia strigosozonata* | Brown rot | https://mycocosm.jgi.doe.gov/Punst1/Punst1.home.html |
| 22 |  |  | *Piloderma olivaceum* | Brown rot | https://mycocosm.jgi.doe.gov/Pilcr1/Pilcr1.home.html |
| 23 |  |  | *Fibulorhizoctonia psychrophila* | Symbiotic | https://mycocosm.jgi.doe.gov/Fibsp1/Fibsp1.home.html |
| 24 |  |  | *Sistotremastrum suecicum* | White rot | https://mycocosm.jgi.doe.gov/Sissu1/Sissu1.home.html |
| 25 |  |  | *Tulasnella calospora* | Symbiotic | https://mycocosm.jgi.doe.gov/Tulca1/Tulca1.home.html |
| 26 |  |  | *Rhizoctonia solani* |  | https://mycocosm.jgi.doe.gov/Rhisola1/Rhisola1.home.html |
| 27 |  |  | *Botryobasidium botryosum* | White rot | https://mycocosm.jgi.doe.gov/Botbo1/Botbo1.home.html |
| 28 |  | Dacrymycetes | *Dacryopinax spathularia* | White rot | https://mycocosm.jgi.doe.gov/Dacsp1/ Dacsp1.home.html |
| 29 |  |  | *Calocera viscosa* | Brown rot | https://mycocosm.jgi.doe.gov/Calvi1/Calvi1.home.html |
| 30 |  | Tremellomycetes | *Tremella mesenterica* |  | https://mycocosm.jgi.doe.gov/Treme1/Treme1.home.html |
| 31 |  |  | *Cryptococcus neoformans* |  | https://mycocosm.jgi.doe.gov/Cryne_H99_1/Cryne_H99_1.home.html |
| 32 |  | Pucciniomycetes | *Melampsora laricis-populina* |  | https://mycocosm.jgi.doe.gov/Mellp2_3/Mellp2_3.home.html |
| 33 |  | Microbotryomycetes | *Sporobolomyces roseus* |  | https://mycocosm.jgi.doe.gov/Sporo1/Sporo1.home.html |
| 34 |  | Malasseziomycetes | *Malassezia globosa* | Symbiotic | https://mycocosm.jgi.doe.gov/Malgl1/Malgl1.home.html |
| 35 |  | Ustilaginomycetes | *Ustilago maydis* |  | https://mycocosm.jgi.doe.gov/Ustma2_2/Ustma2_2.home.html |
| 36 | Ascomycota | Sordariomycetes | *Cryphonectria parasitica* |  | https://mycocosm.jgi.doe.gov/Crypa2/Crypa2.home.html |
| 37 |  | Eurotiomycetes | *Aspergillus tubingensis* |  | https://mycocosm.jgi.doe.gov/Asptu1/Asptu1.home.html |
| 38 |  | Dothideomycetes | *Parastagonospora nodorum* |  | https://mycocosm.jgi.doe.gov/Stano2/Stano2.home.html |
| 39 |  | Sordariomycetes | *Trichoderma reesei* |  | https://mycocosm.jgi.doe.gov/Trire2/Trire2.home.html |
| 40 |  | Pezizomycetes | *Choiromyces venosus* | Symbiotic | https://mycocosm.jgi.doe.gov/Chove1/Chove1.home.html |
| 41 |  | Saccharomycetes | *Scheffersomyces stipitis* |  | https://mycocosm.jgi.doe.gov/Picst3/Picst3.home.html |
| 42 | Mucoromycota | Mucoromycetes | *Phycomyces blakesleeanus* |  | https://mycocosm.jgi.doe.gov/Phybl2/Phybl2.home.html |
| 43 | Chytridiomycota | Chytridiomycetes | *Batrachochytrium dendrobatidis* |  | https://mycocosm.jgi.doe.gov/Batde5/Batde5.home.html |

**Supporting references**

1. Liu JJ, Peng C, Han QQ, Wang MY, Zhou G, Ye B, Xiao YZ, Fang ZM, Kües U. 2022. *Coprinopsis cinerea* uses laccase Lcc9 as a defense strategy to eliminate oxidative stress during fungal-fungal interactions. Appl Environ Microb 88(1):e0176021. https://doi.org/10.1128/aem.01760-21

2. Gan ZW, Zhang XP, Li MK, Li X, Zhang XL, Wang CK, Xiao YZ, Liu JJ, Fang ZM. 2023. Seryl-tRNA synthetase shows a noncanonical activity of upregulating laccase transcription in *Trametes hirsuta* AH28-2 exposed to copper ion. Microbiol Spectr 11(4):e0076823. https://doi.org/10.1128/spectrum.00768-23

3. Zhang YF, Zhang XL, Zhang XP, Zhao WM, Liu JJ, Wang XT, Xiao YZ, Fang ZM. 2022. ThhspA1 is involved in *lacA* transcriptional regulation of *Trametes hirsuta* AH28-2 exposed to *o*-toluidine. Fungal Genet Biol 161:103716. https://doi.org/10.1016/j.fgb.2022.103716

4. Wang JJ, Chen YY, Dong YQ, Fang W, Fang ZM, Xiao YZ. 2017. A simple and efficient method for successful gene silencing of *HspA1* in *Trametes hirsuta* AH28-2. Anton Leeuw Int J G 110(12):1527-1535. https://doi.org/10.1007/s10482-017-0904-9

5. Katoh K, Toh H. 2008. Recent developments in the MAFFT multiple sequence alignment program. Brief Bioinform 9(4):286-298. https://doi.org/10.1093/bib/bbn013

6. Castresana J. 2000. Selection of conserved blocks from multiple alignments for their use in phylogenetic analysis. Mol Biol Evol 17(4):540-552. https://doi.org/10.1093/oxfordjournals.molbev.a026334

7. Stamatakis A. 2014. RAxML version 8: a tool for phylogenetic analysis and post-analysis of large phylogenies. Bioinformatics 30(9):1312-1313. https://doi.org/10.1093/bioinformatics/btu033

8. Taylor TN, Hass H, Kerp H, Krings M, Hanlin RT. 2005. Perithecial ascomycetes from the 400 million year old Rhynie chert: an example of ancestral polymorphism. Mycologia 97(1):269-285. https://doi.org/ 10.3852/mycologia.97.1.269

9. Floudas D, Binder M, Riley R, et al. 2012. The paleozoic origin of enzymatic lignin decomposition reconstructed from 31 fungal genomes. Science 336(6089):1715-1719. https://doi.org/10.1126/science.1221748

10. Xu GF, Wang JJ, Yin Q, Fang W, Xiao YZ, Fang ZM. 2019. Expression of a thermo- and alkali-philic fungal laccase in Pichia pastoris and its application. Protein Expres Purif 154:16-24. https://doi.org/10.1016/j.pep.2018.09.015

**LacA amino acid sequence**

MSRFQSLLAFVVASLAAVAHAAIGPTADLTISNAEVSPDGFARQAVVVNNVTPGPLVAGNKGDRFQLNVIDNLTNHTMLKSTSIHWHGFFQKGTNWADGPAFVNQCPISSGHSFLYDFQVPDQAGTFWYHSHLSTQYCDGLRGPFVVYDPNDPHASLYDVDNDDTVITLADWYHTAAKLGPAFPLGADATLINGLGRSPSTTAADLAVINVTKGKRYRFRLVSLSCDPNHTFSIDGHDLTIIEVDSINSQPLVVDSIQIFAAQRYSFVLNADQDVGNYWIRANPSFGNVGFAGGINSAILRYDGADPVEPTTTQTTPTKPLNEVDLHPLDTMAVPGSPVAGGVDKAINMAFNFNGTNFFINGASFVPPTVPVLLQIISGAQNAQDLLPSGSVYSLPANADIEISFPATAAAPGAPHPFHLHGHAFAVVRSAGSTVYNYDNPIFRDVVSTGTPAAGDNVTIRFRTDNPGPWFLHCHIDFHLEAGFAVVMAEDIPDVASANPVPQAWSDLCPIYDALDVNDQ

**LacB amino acid sequence**

MGKLQSFVNVVALSLSLSRGVLGAIGPVTDLTISNADVTPDGFTRAAVVANGVFPGPLITGNKGDNFQINVIDNLTNATMLKTTTIHWHGLFQHGTNWADGPAFVNQCPIASGNSFLYDFTVPDQAGTFWYHSHLSTQYCDGLRGPLVVYDPSDPYASMYDVDDDTTVITLSDWYHTAAKLGPAFPPNADSVLINGLGRFAGGNASDLAVITVEQNKRYRFRLVSLSCDPNFTFSIDGHNMTIIEVDGVNHEPLEVDSIQIFASQRYSFVLNATQSVDNYWIRAIPNTGTIDTTGGLNSAILRYSGADIVDPTTNATTSVIPLVETDLVPLDSPAAPGDPVVGGVDLAMNLDFSFNGTNFFINNETFIPPTVPVLLQILSGAQSASDLLPTGSVYTLPLNSTIELSFPITTVNGVTNAPGAPHPFHLHGHAFSVVRSAGSSDYNYVNPVRRDTVSTGNPGDNVTIRFTTDNAGPWFLHCHIDFHLEAGFAIVFAEDTPDTASVNPVPTAWSDLCPTYDALDPSDH

**LacC amino acid sequence**

MNGLRLLPSFASLAVVVSLALNTLAGIGPVTDLTISNENVSPDGFTRAAVVANGKAPGPLITGQKGDRFQINVVNKLSNHTMLKSTSIHWHGFFQKGTNWADGPAFVNQCPIATGHSFLYDFQVPDQAGTFWYHSHLSTQYCDGLRGPFVVYDPNDPNASLYDVDNDDTVITLADWYHVAAKLGPAFPPRSDATLINGLGRTSDTPNADLAVITVTTGKRYRFRLISLSCDPAYTFSIDNHDMTIIEADGVNTQQLTVDSLQIFAGQRYSFVLEANQKSGNYWVRANPLFGTTGFAGGINSAILRYDDAVPAEPTSEQGTSTKPLKETDLHPLTAMPVPGSAVSGGVDKAINFAFTFNGTNFFINGATFQPPTTPVLLQILSGAQDAKDLLPSGDVYALPSDATIELSFPASTGAPGAPHPFHLHGHTFAVVRSAGSTEYNYDNPIWRDVVSTGTPQAGDNVTIRFRTDNPGPWFLHCHIDFHLEAGFAVVMAEDIPDTKLANPVPQAWSDLCPIYDALDENDL

**LacD amino acid sequence**

MAGLQRFAFFVALALVGSALAAIGPKANLVITDAAIAPDGFLRDAIVTNGVFPGPLIKGNKGDRFQLNVIDNLSNHTMLKSTSIHWHGFFQAGTNWADGPAFVNQCPIASGHSFLYDFHVPDQAGTFWYHSHLSTQYCDGLRGPFVVYDPKDPHASRYDVDNESTVITLSDWYHTAARLGPRFPLGADSTLINGLGRSSSTPTADLAVINVQRGKRYRFRLVSLSCDPNHTFSIDGHNLTVIEVDGINSKPLTVDSIQIFAAQRYSFVLNANQPVGNYWVRANPNFGTTGFAGGINSAILRYQGAPNVEPTTTQTPSDIPLIETNLHPLSKMTVPGKATIGGVDKALNLGFTFNGTNFFINDATFTPPTVPVLLQILSGAQTAQDLLPAGSVYPLPAHSSIEITLPASAAAPGFPHPFHLHGHAFAVVRSAGSPAYNYADPIWRDVVSTGVPGDNVTIRFRTDNPGPWFLHCHIDFHLEAGFAVVFAEDVPDVKAANPVPKAWSDLCPIYDRLGEGDL

**LacE amino acid sequence**

MGRFSSLYAFTAVTLSLGRLSSAAIGPVADLTISNADVSPDGFTRAAVVVNGVFPGPLITGNMGDNFQLNVIDNLTNATMLKSTTIHWHGFFQKGTNWADGPAFVNQCPIATGNSFLYDFTATDQAGTFWYHSHLSTQYCDGLRGPMVVYDPNDPHADLYDVDDESTVITLADWYHTAAKLGPAFPVGSDAVLINGLGRFSGGNSTDLAVINVEQGKRYRFRLVSLSCDPNFTFSIDGHNMTVIEVDAVNHEPLTVDSIQIFAGQRYSFVLTADQDIDNYFIRALPNAGTVSFTGGVNSAILRYSGAAEIEPTTEFVTSTNPLVETDLVPLDDPAAPGDPVVGGVDYALNLDFSFNGSNFFINDATFVPPTVPVLLQILSGASSAADLLPSGSLFSLPANSSIEISFPITATNAPGAPHPFHLHGHTFSVVRSAGSSEANYVNPVRRDVVSTGTAGDNVTIRFTTDNPGPWFLHCHIDFHLEAGFAIVFSEDTSEVSSFTSPSSAWSDLCPTYDALDSSDL

**LacF amino acid sequence**

MSFSSLRRSLVFLGVCGGAFAAIGPVTELDIVNKVIAPDGVARDTVLAGGTFPGPLIKGQKGDNFRINVVDKLVNETMLTATTIHWHGMFQHTTNWADGPAFVTQCPITTGHDFLYNFNVPDQAGTFWYHSHLALQYCDGLRGPLVIYDPYDPQAHLYDVDDESTVITLADWYHSPAPLLPPAALSDSTLINGLGRWPGNPTADLAVIEVEHGKRYRFRLVSTSCDPNYNFTIDGHSMTIIEADGENTQPLEVDGLQIFAAQRYSFVLNANQPVNNYWIRANPNRANTTGFSNGINSAILRYKGAPIKEPTTNQSRIVNFLNETHLHPLTDARAPGLPFKGGVDYALNLNLTFNGTEFFINDAPFVPPTVPVLLQILNGTLDAHDLLPPGSVYNLPPYSTIELSIPGGVTGGPHPFHLHGHSFSVVRSAGSPHYNYVNPVKRDTVSIGIGGDNVTVRFVTDNPGPWFLHCHIDFHLQAGLAIVFAEDTKDTKLVNPVPKDWEKLCPLFDESMHINP

**LacAnc160 amino acid sequence**

MMMMLLLLALLAALLAAVAAAAIGPVADLTIVNNKEVAPDGFNRSAVLANGTFPGPLIKANKGDNFQINVINQLDDTTMLRSTSIHWHGIFQHGTNSMDGPAFVTQCPIAPGHSFLYKFSVPDQAGTFWYHSHLSTQYCDGLRGPLVIYDPNLDPYKHLYDVDESTVITLADWYHTPAPQVPPPPTWPDSTLINGKGRYGGPQSPLAVINVEQGKRYRFRLINMSCDPNFTFSIDGHKMTVIEADGVNTEPLTVDSIQIFAGQRYSFILNANQPVDNYWIRALPNVTSCGGAAGGFDNGINSAILRYAGAPNAEPTTSQTTSTSRTLNESNLHPLVNPAAPGKPTPGGADNSLNLNISLNATSARFTVNGVSYVPPSVPVLLQILSGAGTNAQDGLLPKGSVYTLPRNKVIEISIPGAIGGGHPFHLHGHTFSVIRSAGSSTYNYVNPVRRDVVSIGSAGNVTIRFTTDNPGPWFLHCHIDWHLEAGLAVVFAEDPEDSTDPTVPEAWKDLCPIYNAAFDALPPSEQSPLLPSGRGASIV

**LacAnc169 amino acid sequence**

MMMMLLLLALLALLLAAVAAAAIGPVTDLHIVNNKEIAPDGFNRSAVLAGGTFPGPLIKANKGDNFQINVIDQLDDTTMLRSTSIHWHGIFQHGTNWADGPAFVTQCPIAPGHSFLYKFNVPDQAGTFWYHSHLSTQYCDGLRGPLVIYDPNLDPYKHLYDVDESTVITLADWYHTPAPQVPPPPTWPDSTLINGKGRYGGPASPLAVINVEQGKRYRFRLVSMSCDPNFTFSIDGHNMTIIEADGVNTEPLTVDSIQIFAGQRYSFVLNANQPVDNYWIRAQPNVTSCGGAAGGFDNGINSAILRYAGAPNTEPTTSQTTSTSRTLVETNLHPLENPAAPGKPTPGGADNNINLNLSFNATSARFTVNGVSFVPPSVPVLLQILSGAGKTAQDGLLPKGSVYTLPRNKVIEISIPGAIGGGHPFHLHGHTFSVVRSAGSSTYNYVNPVRRDVVSIGSAGNVTIRFTTDNPGPWFLHCHIDWHLEAGFAVVFAEDTEDATDPPVPEAWKDLCPIYNAAFDALPPSEQSLLLPSGRGASVA

**LacAnc178 amino acid sequence**

MMSMLSLLAALALLLAALAAAAIGPVTDLHIVSNAEIAPDGFNRSAVLAGGTFPGPLITGNKGDNFQINVIDQLTDTTMLKSTSIHWHGIFQHGTNWADGPAFVNQCPIAPGNSFLYDFNVPDQAGTFWYHSHLSTQYCDGLRGPLVIYDPNLDPHASLYDVDESTVITLADWYHTVAPSAAPPPTWPDSTLINGLGRYGGPASDLAVISVEQGKRYRFRLVSISCDPNFTFSIDGHNMTIIEADGVNTEPLTVDSIQIFAGQRYSFVLNANQPVDNYWIRANPNLTGCGGATTGFAGGINSAILRYSGADETEPTTTQTTSTSRTLVETNLHPLENPAAPGKPTVGGADVNINLDLAFNATSFNFTINGVTFVPPTVPVLLQILSGAGQSASDGLLPSGSVYTLPRNKTIEISMPGAAGGGHPFHLHGHTFDVVRSAGSSTYNYVNPVRRDVVSIGTAGNVTIRFTTDNPGPWFLHCHIDWHLEAGFAVVFAEDTADATDPPVPTAWNDLCPIYDAAFDALTPDELGLLLPSGRGGRVA
